# H3K27me3 is vital for fungal development and secondary metabolite gene silencing, and substitutes for the loss of H3K9me3 in the plant pathogen *Fusarium proliferatum*

**DOI:** 10.1371/journal.pgen.1011075

**Published:** 2024-01-02

**Authors:** Lena Studt-Reinhold, Anna K. Atanasoff-Kardjalieff, Harald Berger, Celine Petersen, Simone Bachleitner, Michael Sulyok, Alica Fischle, Hans-Ulrich Humpf, Svetlana Kalinina, Teis Esben Søndergaard

**Affiliations:** 1 University of Natural Resources and Life Sciences, Vienna, Department of Applied Genetics and Cell Biology, Institute of Microbial Genetics, Tulln an der Donau, Austria; 2 Aalborg University, Department of Chemistry and Bioscience, Aalborg, Denmark; 3 University of Natural Resources and Life Sciences, Vienna, Department of Agrobiotechnology, Institute of Bioanalytics and Agro-Metabolomics, Tulln an der Donau, Austria; 4 University of Münster, Institute of Food Chemistry, Münster, Germany; Australian National University Research School of Biology, AUSTRALIA

## Abstract

Facultative heterochromatin marked by histone H3 lysine 27 trimethylation (H3K27me3) is an important regulatory layer involved in secondary metabolite (SM) gene silencing and crucial for fungal development in the genus *Fusarium*. While this histone mark is essential in some (e.g., the rice pathogen *Fusarium fujikuroi*), it appears dispensable in other fusaria. Here, we show that deletion of *FpKMT6* is detrimental but not lethal in the plant pathogen *Fusarium proliferatum*, a member of the *Fusarium fujikuroi* species complex (FFSC). Loss of FpKmt6 results in aberrant growth, and expression of a large set of previously H3K27me3-silenced genes is accompanied by increased H3K27 acetylation (H3K27ac) and an altered H3K36me3 pattern. Next, H3K9me3 patterns are affected in Δ*fpkmt6*, indicating crosstalk between both heterochromatic marks that became even more obvious in a strain deleted for *FpKMT1* encoding the H3K9-specific histone methyltransferase. In Δ*fpkmt1*, all H3K9me3 marks present in the wild-type strain are replaced by H3K27me3, a finding that may explain the subtle phenotype of the Δ*fpkmt1* strain which stands in marked contrast to other filamentous fungi. A large proportion of SM-encoding genes is allocated with H3K27me3 in the wild-type strain and loss of H3K27me3 results in elevated expression of 49% of them. Interestingly, genes involved in the biosynthesis of the phytohormones gibberellins (GA) are among the most upregulated genes in Δ*fpkmt6*. Although several FFSC members harbor GA biosynthetic genes, its production is largely restricted to *F*. *fujikuroi*, possibly outlining the distinct lifestyles of these notorious plant pathogens. We show that H3K27me3 is involved in GA gene silencing in *F*. *proliferatum* and at least one additional FFSC member, and thus, may serve as a regulatory layer for gene silencing under non-favoring conditions.

## Author summary

H3K27me3 is a hallmark of facultative heterochromatin, and as such tightly connected with gene silencing. In the genus *Fusarium*, a large proportion of the genome is allocated with this histone mark. Not surprisingly its removal is detrimental and even lethal for some fusaria e.g., the plant pathogen *Fusarium fujikuroi* for still unknown reasons. Here, we show that Kmt6 is vital but not essential in the closely related *Fusarium proliferatum*, allowing for the first time a comprehensive analysis of Kmt6 loss-of-function in a member of the *Fusarium fujikuroi* species complex (FFSC). By an “omics”-oriented approach, we show the genome-wide distribution of activating and silencing histone marks in the wild-type as well as a Δ*fpkmt6* strain, mapped on a newly annotated genome assembled to near chromosome level. Loss of H3K27me3 and H3K9me3 involved in facultative- and constitutive heterochromatin, respectively, revealed a crucial crosstalk between both histone marks in this genus. These results may explain the subtle phenotype of *KMT1* deletion exhibited in this but also in other fusaria, which stands in marked contrast to other fungal species.

## Introduction

The genus *Fusarium* comprises over 400 phylogenetically distinct species [1], and many of them are important plant pathogens [2]. *Fusarium*-induced crop diseases cost the global agricultural economy multi-billion-euro losses each year [3, 4]. This is not least due to their extraordinary capacity to produce natural products of small molecular weight (so-called secondary metabolites, SMs) with often toxic properties i.e., mycotoxins [5, 6]. The *Fusarium fujikuroi* species complex (FFSC) comprises important members of these notorious plant pathogens, as *F*. *verticillioides*, *F*. *fujikuroi* and *F*. *proliferatum* are well-known to cause devastating cereal diseases along with the contamination of food and feed with potent mycotoxins including but not limited to fumonisins, moniliformin, fusaric acid or fusarins [7–9]. Published genome sequences suggest an even much higher chemical potential illustrated by the presence of many additional SM-related genes. Genes involved in the biosynthesis of individual SMs are often physically linked [10], also referred to as biosynthetic gene clusters (BGCs). Noteworthy, the genetic capacity often differs greatly from the portfolio of SMs produced by the fungus. SM biosynthesis is costly in energy and SMs *per se* are not essential for growth, but they may pose selective advantages to the producing organism under certain environmental conditions [11, 12], and as such may very well be vital for survival. Due to this, only a fraction of them is produced in axenic cultures, thereby explaining the discrepancies between the genetic and chemical SM portfolio. This context-specific gene expression requires a tight regulatory network functioning on several different levels involving signaling components, pathway-specific as well as global regulators, and histone posttranslational modifications (or histone marks) that modify the chromatin landscape [13].

Chromatin as determined by histone marks plays a pivotal role in fungal SM gene regulation shown for e.g., *Aspergillus nidulans* [14, 15], *Epichloë festucae* [16, 17], *Penicillium chrysogenum* [18], *Colletotrichum higginsianum* [19], and several *Fusarium* spp. [20–28]. A breakthrough towards understanding the chromatin-level regulation of *Fusarium* SM genes was the discovery that a large proportion of histones associated with silent BGCs is trimethylated at histone H3 lysine 27 (H3K27me3) [20, 23]. H3K27me3 is a repressive histone mark and is typically associated with chromosomal regions subjected to a dynamic regulation, also called facultative heterochromatin [29]. Consequently, removing H3K27me3 induced expression of otherwise silent BGCs in *Fusarium* [20, 23, 24, 30]. Although being widely conserved among Ascomycota, some genera even completely lack Kmt6 orthologs e.g., *Aspergillus* and *Penicillium* [31]. Noteworthy, Kmt6 appears to be essential in *F*. *fujikuroi* [23], but not in *F*. *graminearum* [20]. The reasons for this are still unknown. Next to H3K27me3, another heterochromatic mark i.e., histone H3 lysine 9 trimethylation (H3K9me3), appears to be required for wild type-like SM gene expression in several fungi including *Fusarium* spp. [22, 28, 32]. H3K9me3 is a hallmark for constitutive heterochromatin and typically associated with centromeric and gene-poor regions. Genes decorated with H3K9me3 are hardly ever expressed [29]. While genome-wide association studies for H3K9me3 are still scarce in filamentous fungi, it is noteworthy to mention, that none of the BGCs present in *F*. *fujikuroi* is decorated with this histone mark [7]. Recently, histone H3 lysine 36 trimethylation (H3K36me3), was shown to co-localize with H3K27me3 in *Neurospora crassa* [33]. This is also true in *F*. *fujikuroi* [23, 26] and *Magnaporthe oryzae* [34]. H3K36me3 is established by two proteins in *Fusarium* and *Neurospora* alike i.e.: the canonical (KMT) Set2 that is tightly associated with RNA PolII elongation and thus marks active genes, and the methyltransferase Ash1 involved in maintaining repression of silent genes [26, 33]. In *N*. *crassa*, loss of Ash1-catalyzed H3K36me3 depletes H3K27me2/3 levels concomitant with an accumulation of H3K27ac and de-repression of affected genes suggesting an interdependency between both histone marks [33]. Here, we describe the role of H3K27me3 in *F*. *proliferatum*, a pathogen that is mainly associated with maize infections causing maize stalk and cob rot [8]. By a combination of whole-genome sequencing and different “omics-” approaches, we show that H3K27me3 patterns in *F*. *proliferatum* are similar compared to other fusaria [20, 23]. Availability of a strain deleted for *FpKMT6* allowed, for the first time, a detailed characterization of this histone mark in a member of the FFSC. Depletion of H3K27me3 induced the expression of previously H3K27me3-methylated genes, which was accompanied by histone H3 lysine 27 acetylation (H3K27ac). We show that facultative heterochromatin (H3K27me3) substitutes for constitutive heterochromatin (H3K9me3) in Δ*fpkmt1*, and that H3K36me3 patterns in Δ*fpkmt6* are distinct from the wild type. Moreover, we provide evidence that heterochromatin is responsible for low to no production of the phytohormones gibberellins (GA) in *F*. *proliferatum* and *F*. *mangiferae*.

## Results and discussion

### FpKmt6 and H3K27me3 are not essential in *F*. *proliferatum* NRRL62905

**Fig 1:**
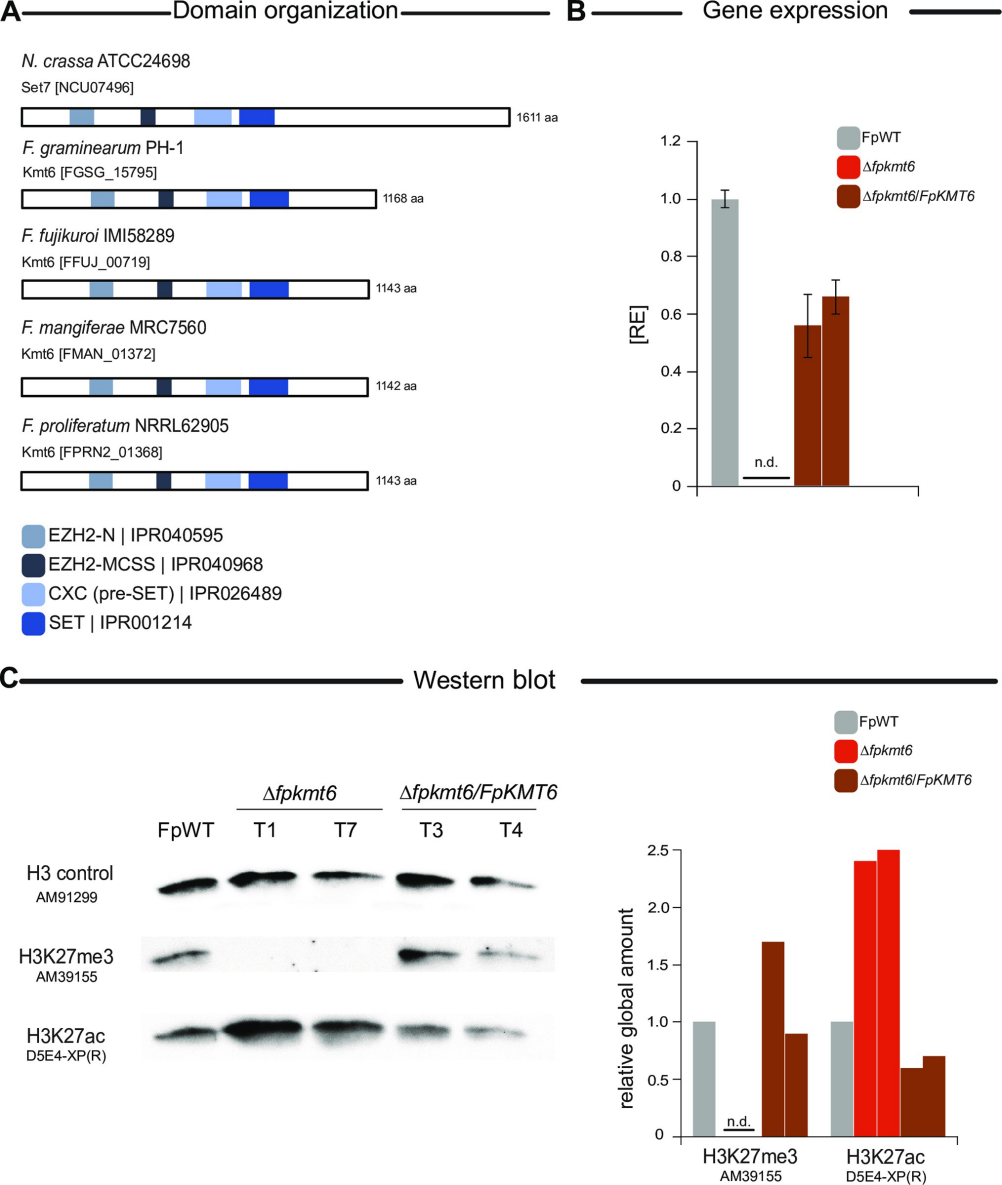
FpKmt6 is the sole H3K27-specific methyltransferase in *Fusarium proliferatum*. (A) Graphical representation of the domain structure of *Neurospora crassa* Set7 and orthologs in other fungal species including *F*. *proliferatum* NRRL62905. The conserved domains are indicated and InterPro accession numbers are given in the domain description (B) For *FpKMT6* expression analysis, FpWT as well as two independent Δ*fpkmt6* and Δ*fpkmt6*/*FpKMT6* strains each were grown on solid CM for 3 days at 30°C. For the determination of transcript levels, RNA was extracted and transcribed into cDNA before RT-qPCR. The FpWT *FpKMT6* expression was arbitrarily set as 1. Mean values and standard deviations are shown. n.d., not detected; RE, relative expression. **(C)** Western blot analysis of FpWT as well as two independent Δ*fpkmt6* and Δ*fpkmt6*/*FpKMT6* strains each using H3K27me3- and H3K27ac-specific antibodies. For referencing an H3pan-specific antibody was used. Roughly, 25 μg of total protein extracts were separated on a SDS gel before western blotting. For quantification, densitometric analysis was performed and the respective wild-type strain was arbitrarily set as 1. n.d., not detected *via* the Image J analysis tool.

Loss of Kmt6 appears lethal in *Fusarium fujikuroi* [23]. To determine whether this trait is unique for *F*. *fujikuroi* or is conserved also in other FFSC members, an attempt to remove the *KMT6* ortholog was approached in the two closely related fusaria, *Fusarium proliferatum* NRRL62905 and *Fusarium mangiferae* MRC7560, from now on referred to as FpWT and FmWT, respectively. The putative *KMT6* orthologs were identified by QuartetS [35]. Function- and phylogeny-based metrics based on large-scale comparison predicted FPRN2_01368 and FMAN_01372 as the true orthologs of the H3K27-specific methyltransferase FfKmt6 (FFUJ_00719) from *F*. *fujikuroi* [23]. Pairwise sequence alignment using ClustalW showed 99.04% and 98.95% identity of FPRN_01368 and FMAN_01372, respectively, with FfKmt6 on the protein level [36]. The typical domains i.e., EZH2-N, EZH2-MCSS, CXC (pre-SET), and SET domains are present in both species (**Fig 1A**) [37]. Deletion of FPRN2_01368 and FMAN_01372 was approached by homologous integration of a hygromycin resistance cassette into the respective wild-type strains, FpWT and FmWT. Several transformants were obtained that showed correct *in situ* integration of the resistance cassette for both transformation approaches (**S1 and S2 Figs**). While the wild-type gene was present and remained detectable even after several consecutive rounds of single spore isolation in the case of *F*. *mangiferae* (**S1C Fig**), several homokaryotic Δ*fpkmt6* mutants were obtained that showed correct *in situ* integration of the resistance cassette and absence of the native *FpKMT6* wild-type gene (**S2B Fig**). Three independent mutant strains, Δ*fpkmt6*_T1, T7, and T9, were selected for further analyses. Southern blot analyses verified the absence of multiple integrations (**S2C Fig**). All mutants had an identical phenotype. Hence, Δ*fpkmt6*_T1 was arbitrarily chosen for complementation. For this, *FpKMT6* driven by its native promoter and followed by the artificial glucanase terminator from *Botrytis cinerea*, BcTgluc, was targeted to the native locus (*in situ*, **S3A Fig**). Obtained mutants, Δ*fpkmt6*/*FpKMT6*, were analyzed by diagnostic PCR (**S3B Fig**), and reconstitution of *FpKMT6* expression was verified by real-time quantitative polymerase chain reaction (RT-qPCR, **Fig 1B**). Finally, to verify the involvement of FpKmt6 in H3K27 methylation, western blot analysis was performed using whole protein extracts of FpWT, Δ*fpkmt6*, and Δ*fpkmt6*/*FpKMT6* strains [28]. While abundant in FpWT and Δ*fpkmt6*/*FpKMT6*, H3K27me3 levels were not detectable in Δ*fpkmt6* (**Figs 1C and S4**). To analyze if H3K27me3 is replaced by H3K27ac in Δ*fpkmt6*, overall H3K27ac levels were determined by western blot analysis using an H3K27ac-specific antibody. H3K27ac levels were increased about 2.5-fold in Δ*fpkmt6* as compared to FpWT, and largely restored to wild-type level in Δ*fpkmt6*/*FpKMT6* (**Figs 1C and S4**). Thus, FpKmt6 is the sole H3K27-specific methyltransferase in *F*. *proliferatum* NRRL62905, and loss of H3K27me3 is not lethal in this strain.

### FpKmt6 is required for fungal growth and asexual development

**Fig 2:**
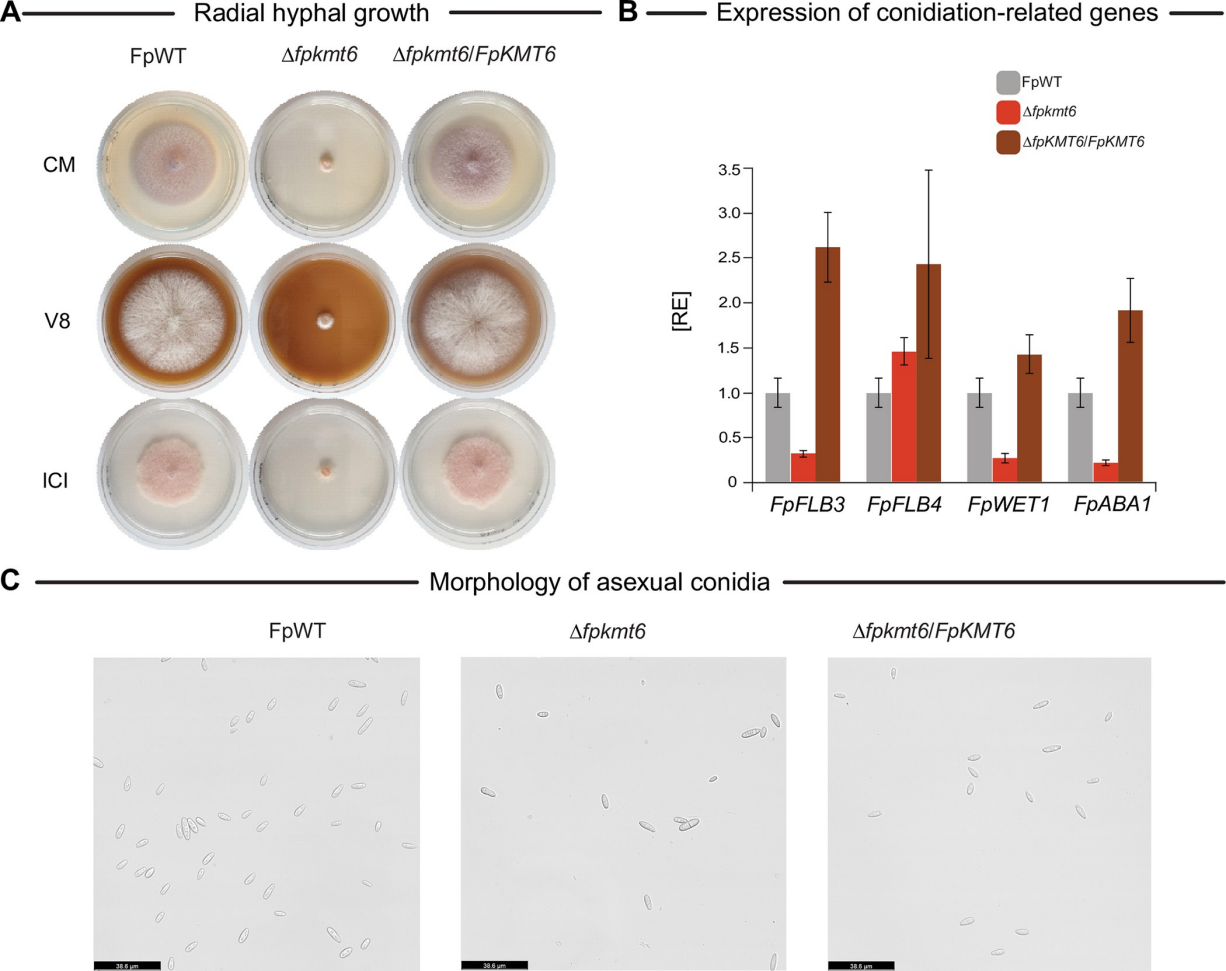
Impact of *FpKMT6* deletion on radial hyphal growth and asexual development. **(A)** Radial hyphal growth of *F*. *proliferatum* wild type (FpWT), Δ*fpkmt6*, and Δ*fpkmt6*/*FpKMT6* was assessed on complete media (FCM, V8) and minimal media (ICI supplemented with 6 mM Gln). For this, strains were grown for 5 days at 30°C in the dark. Experiments were performed in biological triplicates. (**B)** The asexual development of the different strains was assessed under dark conditions. For this, FpWT, Δ*fpkmt6*, and Δ*fpkmt6*/*FpKMT6* were grown on a solid V8 medium for seven days in the dark. Experiments were performed in triplicates. Conidia production of FpWT was arbitrarily set as 1. (C) Microscopic assessment of asexual conidia in FpWT, Δ*fpkmt6*, and Δ*fpkmt6*/*FpKMT6*. The bar in the right corner represents 10 μm in size.

The availability of a homokaryotic Δ*fpkmt6* strain allowed for a detailed functional characterization of FpKmt6 in a member of the FFSC. The impact of FpKmt6 on fungal growth was assessed on complete (vegetable V8-juice, V8, and complete medium, CM), and minimal (synthetic ICI) media supplemented with 6 mM glutamine as the sole nitrogen source. For this, plates were point-inoculated with agar plugs of FpWT, Δ*fpkmt6*, or Δ*fpkmt6*/*FpKMT6* and incubated at 30°C in the dark. Fungal growth was determined five days post-inoculation (dpi). Radial hyphal growth was severely impacted in Δ*fpkmt6* compared to FpWT and restored to wild-type level in the complemented strain Δ*fpkmt6*/*FpKMT6* (**Fig 2A)** on all tested media conditions. To analyze the effect of H3K27me3 depletion on asexual development, conidia formation of FpWT and Δ*fpkmt6* was assessed on V8 agar plates, and conidia were quantified seven dpi. The overall number of conidia is significantly reduced in Δ*fpkmt6* as compared to FpWT but when compared to the radial hyphal growth conidia count in FpWT and Δ*fpkmt6* was similar (**S5 Fig**). Noteworthy, the growth behavior of Δ*fpkmt6* differed from FpWT significantly thereby impeding a proper assessment of the asexual development. Therefore, transcription of conidiation-related genes was quantified by RT-qPCR. In *A*. *nidulans*, conidiation is regulated *via* the so-called ‘fluffy’ proteins FlbB-FlbE, which are required for efficient expression of the central regulator of asexual development BrlA. BrlA in turn activates *abaA*, which further activates *wetA*. Both *abaA* and *wetA* are required for conidia formation and maturation, respectively, in this fungus [38]. While BrlA appears to be absent from the genus *Fusarium* [25, 39], orthologs of *flbB*-*flbE* (*FLB2*-*FLB5*), as well as *abaA* (*ABA1*) and *wetA* (*WET1*) have been identified and functionally characterized in the closely related *F*. *fujikuroi* [25]. Loss of *FLB3*, *FLB4*, and *ABA1* completely abolished conidia formation in this fungus, while the amounts of formed conidia were wild type-like in Δ*flb2*, Δ*flb5*, and Δ*wet1* [25]. To analyze whether expression of the conidiation-related genes is affected by deletion of *FpKMT6* in *F*. *proliferatum*, the respective orthologs were determined by QuartetS [35], using the entries for *F*. *fujikuroi* [25]. The FpWT, Δ*fpkmt6*, and Δ*fpkmt6*/*FpKMT6* were grown for 4 days under conidia-inducing conditions, and expression was determined by RT-qPCR for *FpABA1*, *FpFLB3*, *FpFLB4*, and *FpWET1*. Expression of *FpFLB3* (FPRN2_04427), *FpABA1* (FPRN2_21361), and also *FpWET1* (FPRN2_07602) was reduced to 28%, 23% and 30%, respectively, and largely restored to wild-type level (1.9, 2.6 and 1.4) in Δ*fpkmt6*/*FpKMT6*, while *FpFLB4* (FPRN2_08233) expression remained unaffected (**Fig 2B**). Next to this, deletion of *FpKMT6* did not affect conidia maturation as all harvested conidia had a wild type-like appearance under the microscope (**Fig 2C**). The results are in line with the literature. Declined or even aberrant growth as well as impaired sexual and asexual reproduction was observed also for other fungal mutants lacking a functional Kmt6, including *Ustilaginoidea virens* [40], *Podospora anserina* [41], and also in other fusaria [20, 23]. Only a slight growth reduction and conflicting results with regard to asexual development were reported for *M*. *oryzae* [34, 42]. Interestingly, for *E*. *festucae* and *Zymoseptoria tritici*, only subtle changes in growth and development were observed, implying that H3K27me3 governs different roles in other fungi [16, 43].

### Re-sequencing of FpWT results in near-complete chromosome map

The previous assembly of FpWT was done by whole-genome shotgun sequencing using 454 pyrosequencing and Illumina HiSeq 2000 technologies and resulted in overall 155 scaffolds (43.2 Mb, 98-fold coverage) with an annotated 15,254 gene loci [8]. To generate more complete chromosome sequences and thus to allow for a correct allocation of histone marks, especially concerning larger genomic regions, the MinION sequencing platform from Oxford Nanopore Technologies was applied [44, 45]. This yielded a near-complete chromosome sequence (43.63 Mb, 110-fold coverage) where the previous 155 scaffolds were assembled into twelve scaffolds, representing the twelve chromosomes present in FpWT as determined by pulsed-field gel electrophoresis previously [8], with an annotated 15,362 gene loci.

**Fig 3:**
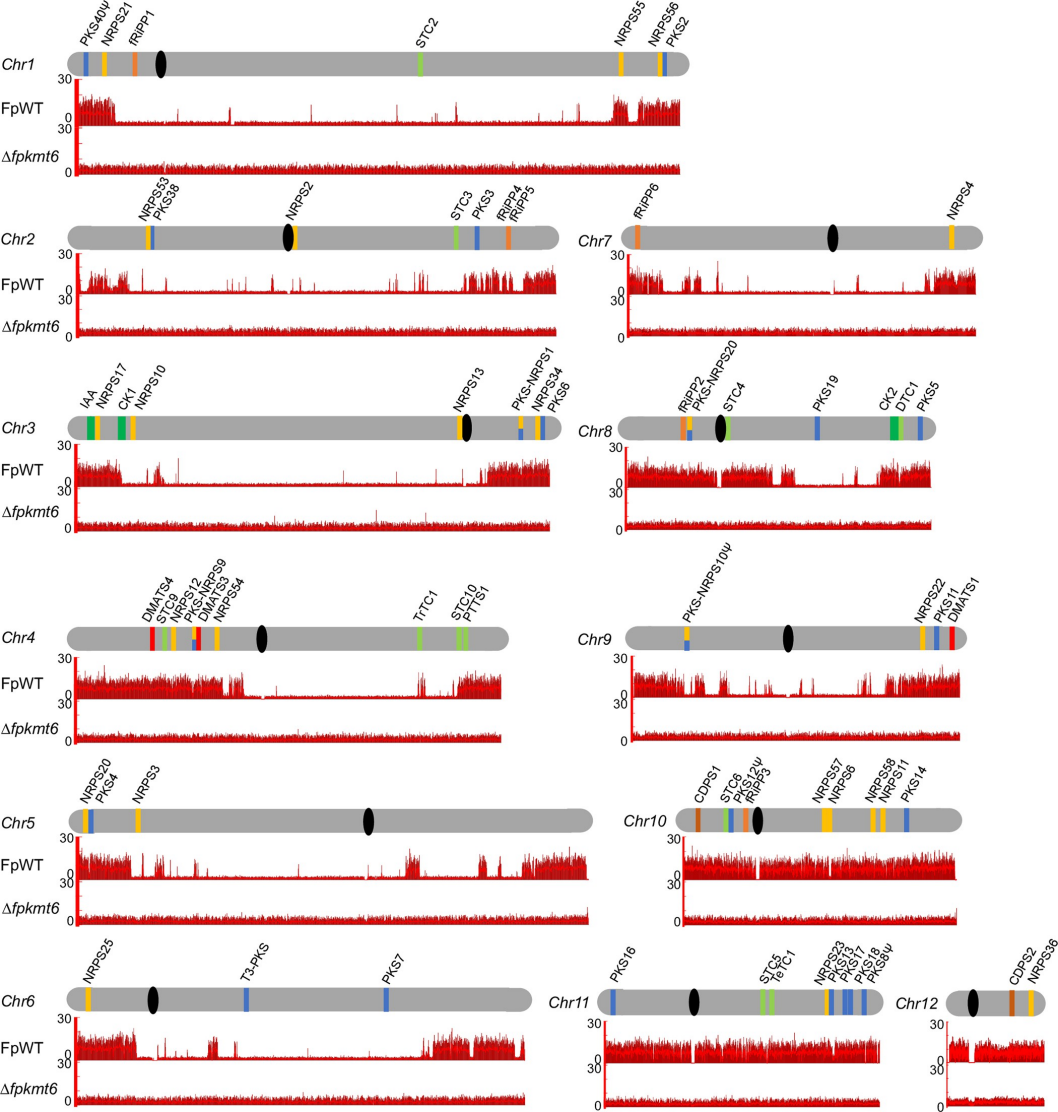
Genomic localization of H3K27me3 and SM key enzyme-encoding genes in *F*. *proliferatum*. The re-sequenced and newly assembled twelve chromosomes are shown in grey i.e., Chr1 –Chr12, and centromeres are shown in black; SM key enzyme-encoding genes are indicated by bars according to the following color code: polyketide synthase (PKS), blue; non-ribosomal peptide synthetase (NRPS), orange; (sesqui-/di-/sester-/tri-/tetra-) terpene cyclase (STC/DTC/PTTS/TrTC/TeTC), light green; dimethylallyl tryptophan synthase (DMATS), red; putative fungal RIPPs, light brown; cyclodipeptide synthase (CDPS), dark brown. Pseudogenes are highlighted with a ψ. Genome-wide distribution of H3K27me3, present in the wild-type strain (FpWT) and absent from the *KMT6*-deleted strain (Δ*fpkmt6*), are depicted in red bars in the two lanes beneath each respective chromosome.

To determine the genome-wide distribution of H3K27me3, chromatin immunoprecipitation (ChIP) coupled to NGS was performed. For this, the wild-type strain FpWT was grown for 3 days in a synthetic minimal medium (liquid ICI supplemented with 6 mM glutamine as sole nitrogen source), crosslinked, and subsequently used for ChIP using an anti-H3K27me3 antibody. Reads obtained from NGS were mapped on the new assembly. H3K27me3 is highly abundant in FpWT covering nearly 45% of all annotated genes (6,805 of 15,362). Similar to other *Fusarium* spp. [20, 23], *P*. *anserina* [41], *U*. *virens* [46], and *M*. *oryzae* [34, 42] but different to *Z*. *tritici* [43] a high percentage of BGCs are allocated with H3K27me3 in *F*. *proliferatum* (**Fig 3**). No H3K27me3 was detected in strains deleted for *FpKMT6* as determined by ChIP-seq, thereby confirming the specificity of the used antibody and the absence of other H3K27me3-specific methyltransferases in this fungus (**Fig 3**).

*F*. *proliferatum* is known to produce a wide range of SMs including the well-characterized red pigment bikaverin, or the mycotoxins fusaric acid and fumonisins [8]. Given the new gene models, the presence of known but also additional BGCs was re-evaluated using antiSMASH 6.0 [47]. The new genome assembly of FpWT revealed 59 putative key enzyme-encoding genes possibly involved in SM biosynthesis: 22 NRPSs, 17 PKSs and 4 PKS-NRPSs, 11 terpene synthases (TSs), 3 dimethylallyltryptophan synthases (DMATSs), and 1 type III PKS (Table 1). Overall, 42 of the 59 putative BGCs, 5 of the 6 fungal RiPP-like genes and both putative CDPSs fall within facultative heterochromatin (**Fig 3**).

**Table 1:** SM key enzyme-encoding genes in FpWT as determined by antiSMASH 6.0. Genes upregulated in Δ*fpkmt6* are highlighted in green, and genes downregulated in red. Genes resulting in non-functional proteins are marked by an Ψ.

Gene	Product	Gene ID	H3K27me3	Δ
Non-ribosomal peptide synthetases				
*FpNRPS2*	Ferricrocin	FPRN2_22646	-	
*FpNRPS3*		FPRN2_26759	-	
*FpNRPS4*	Fusahexin	FPRN2_30438	+	
*FpNRPS6*	Fusarinine	FPRN2_13643	-	
*FpNRPS10*		FPRN2_23678	-	
*FpNRPS11*		FPRN2_13843	+	
*FpNRPS12*		FPRN2_06570	+	
*FpNRPS13*		FPRN2_04074	-	
*FpNRPS17*	Ferrichrome	FPRN2_23563	+	
*FpNRPS20*		FPRN2_07029	+	
*FpNRPS21*		FPRN2_00102	+	
*FpNRPS22*	Beauvericin	FPRN2_12971	+	
*FpNRPS23*		FPRN2_34114	+	
*FpNRPS25*		FPRN2_04017	+	
*FpNRPS34*	Fusaric acid	FPRN2_03685	+	
*FpNRPS36*		FPRN2_15183	+	
*FpNRPS53*		FPRN2_02333	-	
*FpNRPS54*		FPRN2_06362	+	
*FpNRPS55*		FPRN2_21757	+	
*FpNRPS56*		FPRN2_21914	+	
*FpNRPS57*		FPRN2_32910	+	
*FpNRPS58*		FPRN2_33084	+	
Polyketide synthases				
*FpPKS2*		FPRN2_01964	+	
*FpPKS3*	Fusarubins	FPRN2_03394	-	
*FpPKS4*	Bikaverin	FPRN2_07054	-	
*FpPKS5*		FPRN2_31439	+	
*FpPKS6*		FPRN2_03675	+	
*FpPKS7*		FPRN2_09408	-	
*FpPKS8* Ψ	Fusamarins	FPRN2_15017	+	
*FpPKS11*	Fumonisins	FPRN2_42368	+	
*FpPKS12* Ψ		FPRN2_32556	+	
*FpPKS13*	Gibepyrones	FPRN2_14947	+	
*FpPKS14*		FPRN2_33206	+	
*FpPKS16*		FPRN2_14120	+	
*FpPKS17*		FPRN2_14992	+	
*FpPKS18*		FPRN2_15000	+	
*FpPKS19*	Fujikurin	FPRN2_11365	-	
*FpPKS38*		FPRN2_02335	-	
*FpPKS40* Ψ	Fusapyrone	FPRN2_00002	+	
Type III PKS		FPRN2_09028	-	
Hybrid polyketide synthases—non-ribosomal peptide synthetases				
*FpPKS-NRPS1*	Trichosetin	FPRN2_03782	+	
*FpPKS-NRPS9*		FPRN2_25508	+	
*FpPKS-NRPS10* Ψ	Fusarins	FPRN2_12214	+	
*FpPKS-NRPS20*	Sambutoxin	FPRN2_11813	+	
Dimethylallyltryptophane synthases				
*FpDMATS1*	*r*-N-DMAT	FPRN2_13086	+	
*FpDMATS3*		FPRN2_25520	+	
*FpDMATS4*		FPRN2_25325	+	
Terpene synthases				
*FpSTC2*		FPRN2_01122	-	
*FpSTC3*	Eremophilene	FPRN2_03310	-	
*FpSTC4*	Koraiol	FPRN2_11694	+	
*FpSTC5*	Guaiadien	FPRN2_14684	+	
*FpSTC6*	Acorenol	FPRN2_13245	+	
*FpSTC9*		FPRN2_25375	+	
*FpSTC12*		FPRN2_26417	+	
*FpDTC1*		FPRN2_08492	+	
*FpPTTS1*	Fusaproliferin	FPRN2_05485	+	
*FpTrTC1*	Lanosterol	FPRN2_05680	-	
*FpTeTC1*	Carotenoids	FPRN2_14723	+	
Ribosomally synthesized and post-translationally modified peptides (fungal RiPP-like)				
*fRiPP-like1*		FPRN2_00217	-	
*fRiPP-like2*		FPRN2_11838	+	
*fRiPP-like3*		FPRN2_13331	+	
*fRiPP-like4*		FPRN2_23340	+	
*fRiPP-like5*		FPRN2_23343	+	
*fRiPP-like6*		FPRN2_29457	+	
Cyclodipeptide synthases				
*FpCDPS1*		FPRN2_32462	+	
*FpCDPS2*		FPRN2_15133	+	

Thus, 9 additional putative SM key enzyme-encoding genes have been identified. These include one type III PKS-encoding gene, FPRN2_09028, six NRPS-encoding genes, designated NRPS53 through NRPS58 (FPRN2_02333, FPRN2_06362, FPRN2_21757, FPRN2_21914, FPRN2_32910, FPRN2_33084), as well as two putative TS-encoding genes, FPRN2_05485 (PTTS1) and FPRN2_26417 (STC10). FPRN2_05485 harbors a C-terminal prenyltransferase (PT) and an N-terminal terpene synthase (TS) domain, hence designated PTTS1 according to [48]. While the products for most of them are still cryptic, PTTS1 has recently been connected with fusaproliferin biosynthesis in *F*. *proliferatum*, designated as *FpFUP1* [49]. Next to these classical SM key enzyme-encoding genes, FpWT harbors six fungal RiPP-like genes putatively encoding for ribosomal synthesized and post-translationally modified peptides, and two genes encoding putative cyclodipeptide synthases i.e., FPRN2_32462 and FPRN2_15133, designated CDPS1 and CDPS2, respectively, as predicted by antiSMASH 6.0 [47]. CDPSs are involved in the synthesis of cyclodipeptides, also known as diketopiperazines, using two activated amino acid moieties as aminoacyl-tRNAs as substrates [50, 51]. Most CDPSs are promiscuous enzymes, synthesizing one main cyclodipeptide and one or several minor cyclodipeptides. No CDPS-derived product has been identified in *Fusarium* yet. The genomic distribution of the putative BGCs along the twelve chromosomes is depicted in **Fig 3**.

### H327me3 substitutes for the loss of H3K9me3 in *F*. *proliferatum*

Next, we asked whether there is a connection between facultative heterochromatin (H3K27me3) and constitutive heterochromatin (H3K9me3) since both marks seem to take over distinct functions with regard to gene silencing. This includes a possible re-distribution of H3K9me3 in Δ*fpkmt6* and/or H3K27me3 in strains deleted for the H3K9me3-specific histone methyltransferase-encoding gene *FpKMT1*, Δ*fpkmt1*, as shown in other fungi e.g., *N*. *crassa* and *Z*. *tritici* [43, 52]. To analyze whether this is also true for *F*. *proliferatum*, we recorded H3K9me3 and H3K27me3 distributions by ChIP-seq in Δ*fpkmt6* and Δ*fpkmt1*, respectively, also in this species. The *FpKMT1* ortholog was identified by QuartetS [35]. Function- and phylogeny-based metrics based on large-scale comparison predicted FPRN2_27438 as the true ortholog of the H3K9me3 histone methyltransferase FmKmt1 (FMAN_07768) from *F*. *mangiferae* [28]. Pairwise sequence alignment using EMBOSSNeedle showed 97.7% identity of FPRN2_27438 (from now on referred to as FpKmt1), with FMAN_07768 on protein level [36]. Deletion of FPRN2_27438 was approached by homologous integration of a hygromycin resistance cassette into FpWT. Several transformants were obtained that showed correct *in situ* integration of the resistance cassette and the absence of the *FpKMT1* wild-type gene (**S6A Fig**). Western blot analysis showed reduced H3K9me3 levels in three individual-Δ*fpkmt1* strains to about 30% and 40% of FpWT depending on the antibody used (**S6B and S7 Figs**). This is similar to the results obtained for *F*. *mangiferae* [28] and is likely due to the promiscuity of the used antibodies. All obtained mutants showed an identical phenotype on plates (**S6C Fig**). Hence, Δ*fpkmt1*_T4 was arbitrarily chosen for complementation. For this, *FpKMT1* driven by its native promoter and followed by the BcTgluc terminator sequence was targeted to the native locus (*in situ*, **S8A Fig**). Obtained mutants, Δ*fpkmt1*/*FpKMT1* were verified by diagnostic PCR (**S8B Fig**) as well as the absence of growth on hygromycin, and reconstitution of *FpKMT1* expression was verified by RT-qPCR (**S8C Fig**). Thus, similar to *F*. *mangiferae* but distinct from *F*. *fujikuroi* IMI58289 [23, 28], deletion of *KMT1* is not lethal in *F*. *proliferatum*. The Δ*fpkmt1* did not deviate significantly in hyphal growth on solid media or conidiation (**S9A and S9B Fig**), suggesting that H3K9me3 is largely dispensable for fungal development in this *Fusarium* species. Similar results were observed for *F*. *mangiferae* and *F*. *verticillioides* [22, 28]. To determine the genome-wide distribution of H3K9me3, ChIP-seq was performed in FpWT using the previous growth conditions (liquid ICI medium with 6 mM glutamine). In contrast to H3K27me3, only a few H3K9me3-rich islands are present in FpWT. All peaks reside in intergenic regions. This is similar to previous findings for the closely related *F*. *fujikuroi* [7]. Both heterochromatic marks i.e., H3K27me3 and H3K9me3, appeared mutually exclusive along the *F*. *proliferatum* chromosomes in FpWT (**S10 Fig**), which is in agreement with *N*. *crassa* [53] but stands in contrast to other fungi i.e., *P*. *anserina*, *Leptosphaeria maculans* and *Z*. *tritici*, where H3K9me3 and H3K27me3 were largely but not overall devoid from each other [41, 54, 55]. The commercially available H3K9me3-specific antibodies proved promiscuous in western blot analysis (**S8B Fig**). Thus, to cross-validate the obtained data, ChIP-seq was also performed with a H3K9me3-specific antibody in Δ*fpkmt1*. H3K9me3 levels detected in FpWT were nearly abolished in Δ*fpkmt1* samples and remaining low signals in the *kmt1*-deleted strain are likely due to mapping issues as H3K9me3 frequently resides in repeated regions, thereby confirming the specificity of the used H3K9me3 antibody in the ChIP-seq experiments (**S10 Fig**).

**Fig 4:**
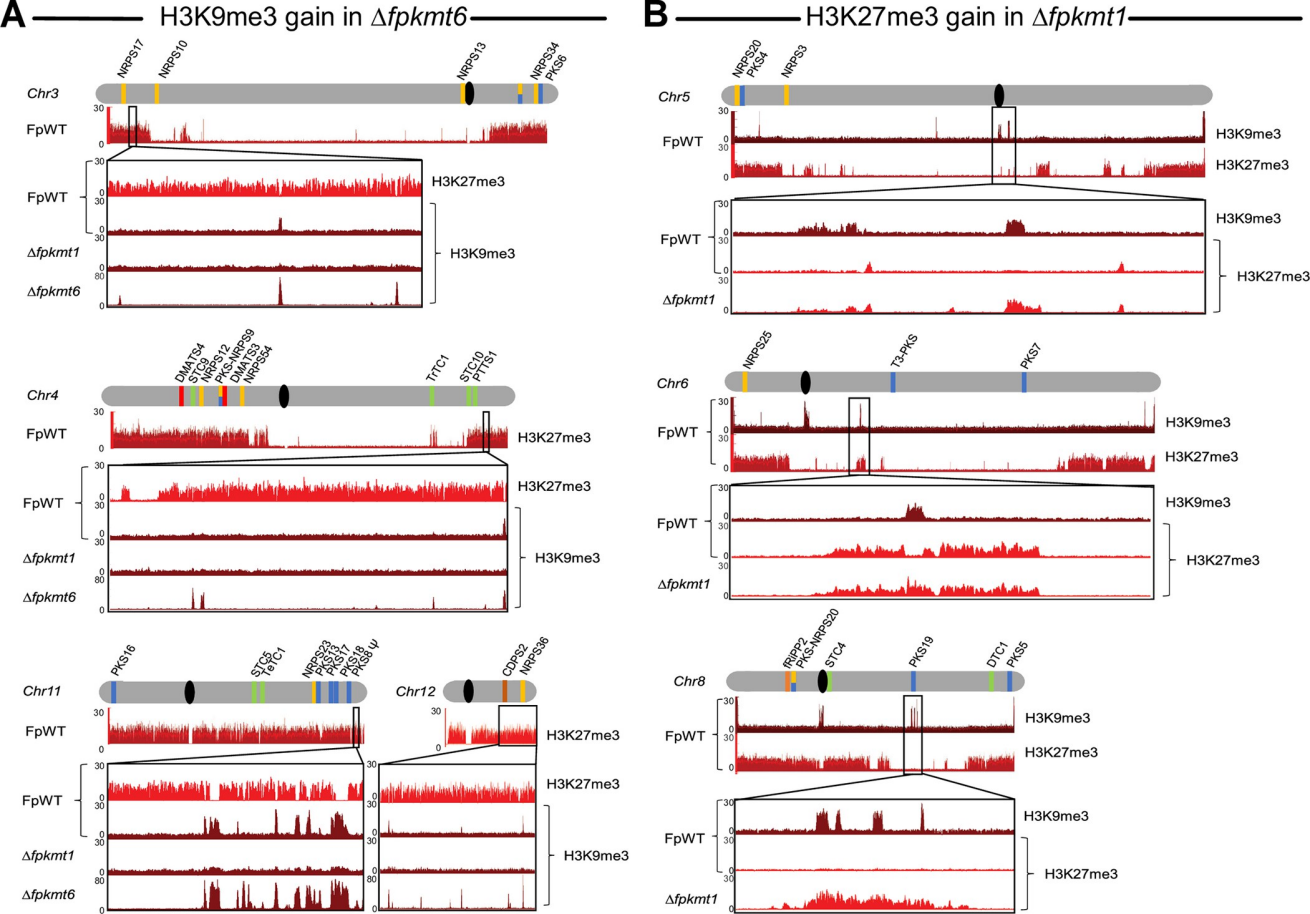
Crosstalk between H3K27me3 and H3K9me3 in *F*. *proliferatum*. (A) Few additional H3K9me3 peaks are gained in previously H3K27me3 intergenic regions in a strain deleted for *FpKMT6* (Δ*fpkmt6*) compared to the wild-type strain (FpWT) as determined by ChIP-sequencing. Here, exemplarily shown for chromosomes 3, 4, 11 and 12. (B) Previously, H3K9me3-marked genomic regions become H3K27me3 upon deletion of *FpKMT1* (Δ*fpkmt1*) compared to FpWT. Here, exemplarily shown for chromosomes 5, 6 and 8. Selected chromosomes are shown in grey and centromeres are shown in black; SM key enzyme-encoding genes are indicated by bars according to the following color code: polyketide synthase (PKS), blue; non-ribosomal peptide synthetase (NRPS), orange; (sesqui-/di-/sester-/tri-/tetra-) terpene cyclase (STC/DTC/PTTS/TrTC/TeTC), light green; dimethylallyl tryptophan synthase (DMATS), red; putative fungal RIPPs, light brown; cyclodipeptide synthase (CDPS), dark brown; Pseudogenes are highlighted, Ψ; Genome-wide distribution of H3K9me3 and H3K27me3 are shown beneath each chromosome in brown and red, respectively.

To determine whether H3K9me3 is re-positioned upon loss of H3K27me3, ChIP-seq was also performed in the Δ*fpkmt6* strain using a H3K9me3-specific antibody. The first most noticeable result was that H3K9me3 detection was better (signal-to-noise ratio) in Δ*fpkmt6* strain, possibly attributable to a higher shearing efficiency of the less dense mycelium. However, this was only detectable for H3K9me3, while other used antibodies had similar efficiencies between FpWT and Δ*fpkmt6*. While H3K9me3 patterns in Δ*fpkmt6* look wild type-like on most of the chromosomes, there are indeed a few peaks gained in previously H3K27me3-rich regions (**Figs 4A, S11 and S12**). These peaks appear exclusively in intergenic regions and are specific to previous H3K27me3-rich regions in FpWT. The function of these emerging H3K9me3 peaks remains elusive at this point and needs further attention in the future. This, however, stands in marked contrast to *P*. *anserina* and *Z*. *tritici*. For *P*. *anserina* absence of H3K27me3 led to a drastic reduction of H3K9me3 levels [41, 43, 52].

Next, we analyzed whether H3K27me3 patterns are affected by deletion of *FpKMT1*. For this, Δ*fpkmt1* was also included in the experiments, and ChIP-seq was performed using a H3K27me3-specific antibody. H3K27me3 patterns in Δ*fpkmt1* largely resemble H3K27me3 of the wild-type strain with one marked exception: previous H3K9me3 peaks become decorated with H3K27me3 in Δ*fpkmt1* (**S13 Fig**). This is most prominent at chromosomes 5, 6, and 8, that harbor dominant H3K9me3 peaks in FpWT (**Figs 4B and S12**). This is also true for the fungal plant-pathogen *Z*. *tritici*, *P*. *anserina*, and *N*. *crassa*, where H3K27me3 relocation in former H3K9me3 marked regions was observed [41, 43, 52].

Thus, for *F*. *proliferatum* it appears that H3K27me3 can substitute for the loss of H3K9me3 in Δ*fpkmt1*. It is tempting to speculate that this crosstalk is the reason for the subtle phenotype of Δ*fpkmt1*, a phenotype that stands in marked contrast to other fungi. This idea is supported by the fact that attempts to generate homokaryotic ΔΔ*fpkmt1*/*fpkmt6* strains failed in our hands, probably because those strains are not viable.

### Loss of H3K27me3 results in elevated gene expression of former silent genes accompanied by an increase in H3K27ac

**Fig 5:**
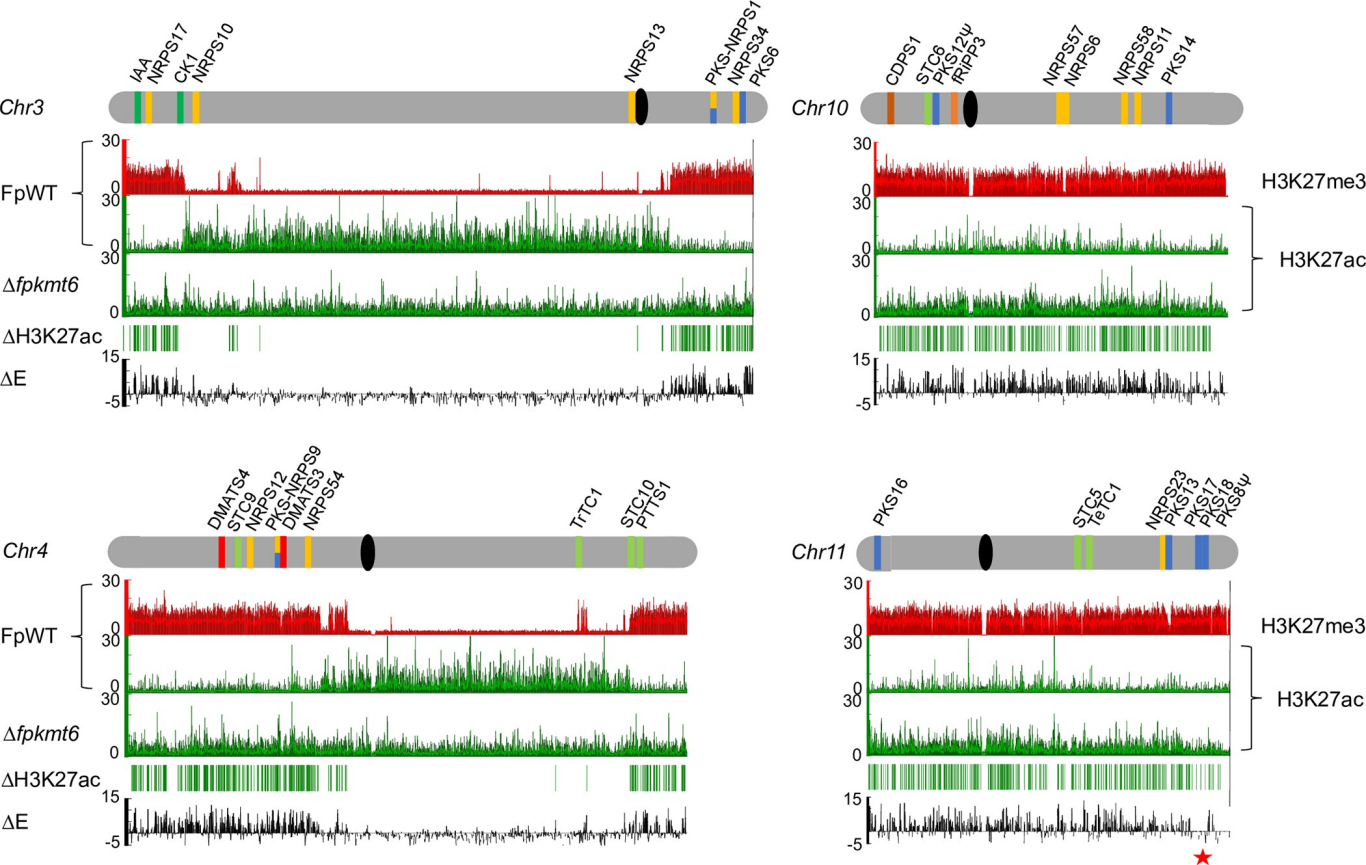
Loss of FpKmt6 results in the de-repression of previous H3K27me3-associated genes accompanied by H3K27ac. Selected chromosomes are shown in grey and centromeres are shown in black; SM key enzyme-encoding genes are indicated by bars according to the following color code: polyketide synthase (PKS), blue; non-ribosomal peptide synthetase (NRPS), orange; (sesqui-/di-/sester-/tri-/tetra-) terpene cyclase (STC/DTC/PTTS/TrTC/TeTC), light green; dimethylallyl tryptophan synthase (DMATS), red; putative fungal RIPPs, light brown; cyclodipeptide synthase (CDPS), dark brown; pseudogenes are marked by a Ψ. Beneath each chromosome in descending order: genome-wide distribution of H3K27me3 (red) and H3K27ac (green) in the wild-type strain (FpWT); H3K27ac in Δ*fpkmt6*, appearance of new H3K27ac peaks in Δ*fpkmt6* as compared to FpWT (ΔH3K27ac); differential expression in Δ*fpkmt6* compared to FpWT (ΔE); genomic region around PKS17, PKS18 and PKS8 on chromosome 11, not de-repressed in Δ*fpkmt6*, is highlighted by an asterisk.

Loss of H3K27me3 led to a strong induction of prior silenced genes, particularly of genes involved in fungal secondary metabolism in *Fusarium* [20, 23, 24]. To determine if this is also the case for *F*. *proliferatum*, whole-transcriptome analysis was performed using RNA-sequencing (RNA-seq). For this, FpWT and Δ*fpkmt6* were grown in a synthetic ICI medium with 6 mM glutamine as the sole nitrogen source, identical to the previously performed ChIP experiments. Indeed, lack of FpKmt6 resulted in the upregulation of a large set of genes specifically localized to previous H3K27me3-marked regions (**Fig 5**). Overall, 4,646 and 3,164 genes are down- and upregulated, respectively, upon deletion *FpKMT6* with a confidence interval of log_2_ ≥ ±1, p-value < 0.01 (**S1 Table**). We used Gene Ontology (GO) based gene set enrichment analysis on subsets of genes either by association with H3K27me3 alone or in conjunction with significant transcriptional upregulation in Δ*fpkmt6* to identify functionalities of genes silenced by H3K27me3. Solely H3K27me3 labeling of genes identified as expected the biological processes (BP): secondary metabolic process (GO:0019748 111 out of 132 genes, p = 2.48E-27) and related ones including antibiotic biosynthetic process (GO:0017000) and mycotoxin metabolic process (GO:0043385); see complete **S2 Table**.

Previously we have shown that H3K27me3 is involved in silencing of genes involved in beauvericin biosynthesis in *F*. *fujikuroi* [24]. Knock-down of *FfKMT6* resulted in reduced H3K27me3 and de-repression of the respective genes, *FfBEA1* –*FfBEA4*, accompanied by elevated H3K27ac as determined by gene-specific ChIP. To evaluate whether this is true also for *F*. *proliferatum* and whether re-decoration of previous H3K27me3 regions with H3K27ac is a general scheme, we performed ChIP-seq using an H3K27ac-specific antibody. Generally, H3K27ac is found in the 5’regions of genes throughout the genome and appears mutually exclusive with H3K27me3 in FpWT. Overall, 42% of the genes are enriched for this histone mark (6,385 of 15,362). Upon removal of H3K27me3, previous H3K27me3-rich regions become replaced by H3K27ac concomitant with a de-repression of the underlying genes (**Fig 5**). This observation was most notable on chromosomes 10 and 11, which are almost completely covered with H3K27me3 in FpWT but hyperacetylated in Δ*fpkmt6* accompanied by a de-repression of a large set of genes located on both chromosomes (**Fig 5**). H3K27ac-rich regions in the FpWT largely stayed acetylated also in Δ*fpkmt6* as determined by ChIP-seq, going in line with an elevated overall H3K27ac level in Δ*fpkmt6* compared to FpWT as determined by western blot (**Fig 1C**). These results align with previous reported results in *M*. *oryzae*. Similar to *F*. *proliferatum*, deletion of *MoKMT6* revealed that a large fraction of genes previously decorated with H3K27me3 were now covered with H3K27ac, which was accompanied by an increased gene expression (fold-change) [34].

### H3K36me3 patterns at deregulated BGCs appear distinct between FpWT and Δ*fpkmt6*

**Fig 6:**
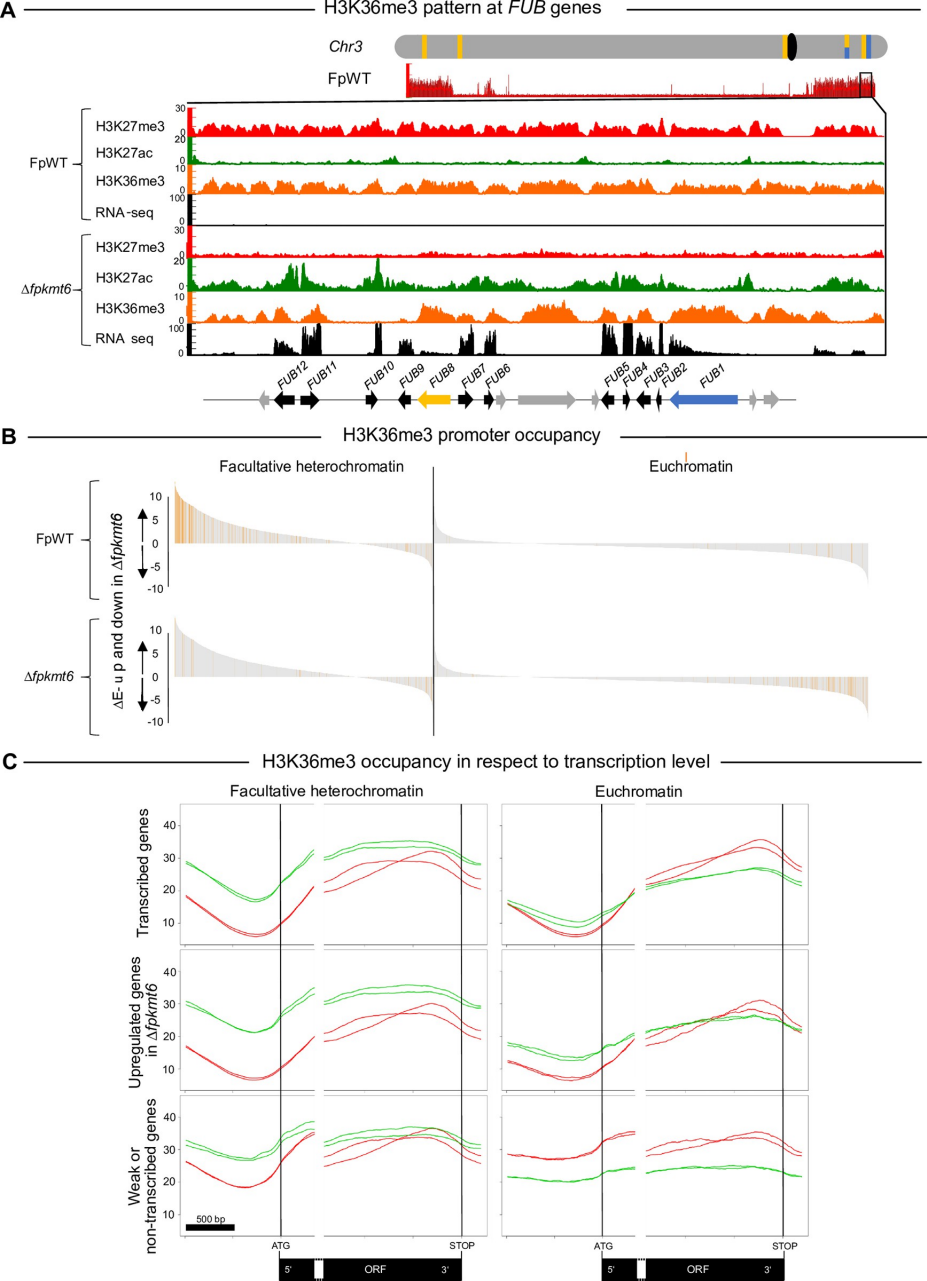
H3K36me3 pattern at de-regulated genes appear distinct between FpWT and Δ*fpkmt6*. (A) Chromatin marks for selected genomic regions of the *F*. *proliferatum* wild-type strain NRRL 62905 (FpWT) and a strain deleted for *FpKMT6* (Δ*fpkmt6*). In descending order are H3K27me3 (red), H3K36me3 (orange), input (grey), and transcription as determined by RNA-seq (black) for FpWT and the same for Δ*fpkmt6*. Below this, the selected BGC is indicated as follows: the PKS- and NRPS-encoding genes are highlighted in blue and yellow, respectively. Other cluster genes are shown in black, and genes not belonging to the FUB cluster are in grey. (B) Grey bar plots represent the differential expression of genes with at least an average transcript level of 0 RPKM. Positive bars represent genes upregulated in Δ*fpkmt6* negative bars represent downregulated genes in Δ*fpkmt6*, indicated by arrow up and down, respectively. Orange bars in the upper lane show genes with H3K36me3-enriched promoters in the wild-type strain (FpWT). Orange bars in the lower lane depict H3K36me3-enriched promoters in Δ*fpkmt6*. (C) H3K36me3 occupancy with respect to the ATG and STOP codons of transcribed genes (mean transcription level > 0 RPKM) and separated into H3K27me3 (facultative heterochromatin, 4420 genes) and euchromatin (not marked with H3K27me3, 7438 genes). The top row is the mean occupancy of all transcribed genes, the middle row shows genes that are significantly upregulated in Δ*fpkmt6* (3016 facultative heterochromatic genes, 445 euchromatic genes), and the third row depicts genes that are transcriptionally silenced (2381 facultative heterochromatic genes, 1114 euchromatic genes) and show high H3K36me3 levels in their promoter regions. Green and red lines depict patterns of FpWT and Δ*fpkmt6* (in replicates), respectively.

In *N*. *crassa* it was previously shown that proper H3K27me2/3 states depend on a catalytically active version of the lysine methyltransferase NcAsh1. NcAsh1 facilitates H3K36me3 at otherwise silent or poorly transcribed genomic regions significantly co-localizing with H3K27me2/3 [33]. To determine whether H3K36me3 depends on H3K27me3 also in *F*. *proliferatum*, we recorded the genome-wide association of H3K36me3 and H3K27me3 in FpWT and Δ*fpkmt6* by ChIP-seq using the same conditions as previously described. While all genes were associated with H3K36me3 in FpWT, some genes appear to have lost this association in the Δ*fpkmt6* strain (**S14 Fig**), the same is true for *M*. *oryzae* [34]. This observation prompted us to have a closer look at the H3K36me3 patterns in *F*. *proliferatum*. In *N*. *crassa*, Ash1- and Set2-catalyzed genes exhibit distinct methylation patterns: while Ash1-catalyzed H3K36me2 was prominent across the promoter and body of the genes, Set2-catalyzed H3K36me2 was depleted from transcriptional start sites (TSS) but enriched over gene bodies [33], which is also true for *F*. *fujikuroi* [26]. Indeed, H3K36me3 patterns at deregulated BGCs in Δ*fpkmt6* deviate from FpWT: BGCs that are silent in FpWT are enriched with H3K36me3 over the TSS and gene bodies, thereby resembling the Ash1-associated methylation pattern, while the TSS of BGCs that are de-repressed in Δ*fpkmt6* resemble more the Set2-associated methylation pattern (**Fig 6A**).

Next, when comparing the differential transcription between FpWT and Δ*fpkmt6* with the differential H3K36me3 occupancy in the promoter regions of genes (minimal [CPM] coverage in 1 kb upstream of the ATG), it became apparent that the majority of genes showing high H3K36me3 in their promoter regions were associated with facultative heterochromatin (**S15A Fig**). The difference in H3K36me3 between Δ*fpkmt6* and FpWT is negatively correlated with the differential transcription between these two strains i.e., high transcription is correlated with reduced H3K36me3 promoter occupancy in Δ*fpkmt6* as compared to FpWT (**S15B Fig**). Euchromatic genes (**Fig 6B**, on the right) also show a different transcriptional response in respect to Δ*fpkmt6* with a majority of genes being downregulated with respect to FpWT. This presumably secondary effect correlates with increased H3K36me3 promoter occupancy of euchromatic genes in Δ*fpkmt6*. However, most of the changes in H3K36me3 occurs in previous H3K27me3-rich regions in Δ*fpkmt6* (**Fig 6B**). Overall, H3K36me3 patterns in euchromatic genes appear similar in both, FpWT and Δ*fpkmt6*, with increased occupancy towards the terminator region to prevent non-sense transcription (**Fig 6C**, top right), and this pattern is mimicked in genes assigned to H3K27me3-rich regions in Δ*fpkmt6* whereas H3K36me3 promoter occupancy is elevated in FpWT. (**Fig 6C**, top left). This is even more pronounced in differentially expressed genes (**Fig 6C**, middle row right). In genes showing low or no transcription (**Fig 6C**, bottom row) H3K36me3 is high in promoter regions which is true for both, facultative heterochromatin and euchromatin. Thus, high H3K36me3 promoter occupancy levels co-occur in facultative heterochromatic regions suggesting that this histone mark is involved in preventing transcription of these genes. The roles of the two H3K36-specific histone methyltransferases, FpSet2 and FpAsh1, are yet unclear, and the general mechanism of targeted promoter H3K36me3 requires further investigation. However, it is intriguing to speculate that, similar to *N*. *crassa* [33], Ash1-mediated H3K36me3 marks TSS and, thus, contributes to SM-gene silencing in FpWT, while FpSet2-catalyzed H3K36me3, generally enriched over the gene bodies but absent from TSS in *N*. *crassa* [33], marks actively transcribed genes at previous H3K27me3 sites in Δ*fpkmt6*.

### Loss of H3K27me3 results in increased BGC expression in *F*. *proliferatum*

Of the 59 predicted key enzyme-encoding genes 16 and 29 were down- and upregulated, respectively, in Δ*fpkmt6*. Next to this, 5 of the 6 fungal RiPP-like genes as well as CDPS1 and CDPS2 were also significantly upregulated in Δ*fpkmt6* within the confidence interval of log_2_ ≥ ±1, p-value < 0.01 (**Table 1**).

All SM key enzyme-encoding genes identified as significantly upregulated in Δ*fpkmt6* are enriched with H3K27me3 in FpWT, while this is the case for only 4 of the 16 downregulated SM key enzyme-encoding genes (**Table 1**). Expression of 3 of them, i.e., *DMATS1* (FPRN2_13086) involved in r-N-DMAT biosynthesis [56] and *NRPS36* (FPRN2_15183) and NRPS54 (FPRN2_06362) was generally very low in both FpWT and Δ*fpkmt6*. It is noteworthy, that the removal of H3K27me3 and/or its replacement with H3K27ac in Δ*fpkmt6* is not sufficient to induce transcription in all gene sets, and that not all previously H3K27-methylated genes become acetylated, which is in accordance with results shown in *M*. *oryzae* [34]. For 19 key enzyme-encoding genes expression was not significantly changed upon deletion of *FpKMT6* although all of them are allocated with H3K27me3 in FpWT. For most of these, expression was generally very low suggesting that additional factors are required to induce their transcription. This is most prominent in the case of the two PKS-encoding genes, PKS17 (FPRN2_14992) and PKS18 (FPRN2_15000), and the remnant of PKS8 (FPRN_15017) [32], all located in close proximity to one another on chromosome 11. None of those genes is de-repressed upon relief of H3K27me3 in Δ*fpkmt6* (**Fig 5**, highlighted with an asterisk). Thus, the removal of H3K27me3 allowed for the expression of 29 SM key enzyme-encoding genes (49%), five out of six fungal RiPP-like genes, and the two putative CDPSs, named CPDS1 and CPDS2. All of them are localized in H3K27me3-rich regions in FpWT (**Fig 3 and Table 1**).

To cross-verify the results obtained by RNA-sequencing, FpWT, Δ*fpkmt6*, and the complemented strain (Δ*fpkmt6*/*FpKMT6*) were grown under identical conditions for subsequent SM analysis. After seven days of growth at 30°C and 180 rpm in the dark, aliquots were taken and analyzed for the most prominent fusarial SMs by targeted metabolomics using LC-MS/MS. To account for the aberrant growth of Δ*fpkmt6*, single cultures were inoculated with twice the volume compared to FpWT and (Δ*fpkmt6*/*FpKMT6*), resulting in a similar final biomass of all strains **(S16 Fig**). Going in line with the expression data (**S1 Table**), biosynthesis of the red pigment bikaverin was almost abolished in Δ*fpkmt6* and restored nearly to wild-type level in the complemented strain Δ*fpkmt6*/*FpKMT6*, while fusaric acid was increased about 140-fold as compared to FpWT and Δ*fpkmt6*/*FpKMT6* (**S17 Fig**). Fusaric acid is normally induced by high nitrogen [57, 58]. Noteworthy, fusaric acid levels (most upregulated genes in the transcriptome) reached amounts in 6 mM glutamine (normally repressing) in Δ*fpkmt6* that were comparable with the amounts produced in FpWT under normally inducing high nitrogen conditions (60 mM glutamine), suggesting that H3K27me3 is responsible for fusaric acid gene-silencing under non-inducing conditions. The distribution of H3K27me3 under different culture conditions was already studied in the plant pathogen *Verticillium dahliae*. Here, ChIP-seq analyses under three different growth conditions revealed overall globally stable H3K27me3 patterns. Expression analysis under these different conditions were correlated with the respective H3K27me3 patterns and results indicated that genes, which are stably covered can be differentially expressed regardless of the chromatin status [59]. If fungi can remove, re-locate H3K27me3 or unwind the tightly packed chromatin structure and, if so, which mechanisms are involved in this phenomenon is currently unknown. Further work is required to prove this assumption unequivocally. Next, two SMs not detectable in FpWT and Δ*fpkmt6*/*FpKMT6* cultures were found to be produced in Δ*fpkmt6* i.e., siccanol (the deacetylated form of fusaproliferin) and beauvericin (**S17 Fig**). The latter was also increased in the RNA-seq experiments though not significantly and hence is not listed in Table 1. This discrepancy is likely attributable to the different time points of measurement and the crippled growth of Δ*fpkmt6*. The BGC involved in fusaproliferin biosynthesis has recently been elucidated [49]. Overall, five genes are involved in fusaproliferin biosynthesis encoding two cytochrome P450 monooxygenases (FPRN2_05484, *FpFUP2* and FPRN2_05488, *FpFUP3*), the sesterterpenoid synthase composed of a C-terminal prenyltransferase and N-terminal terpene synthase (PTTS) (FPRN2_05485, *FpFUP1*), a FAD-linked oxidase (FPRN2_05486, *FpFUP4*), and an acyltransferase (FPRN_05487, *FpFUP5*). Two putative transcription factor-encoding genes are located directly downstream of the BGC, but their involvement in fusaproliferin biosynthesis is still unknown. All five *FUP* genes are silenced by H3K27me3 in FpWT and significantly de-repressed in Δ*fpkmt6* accompanied by H3K27ac under the chosen conditions (**S1 Table** and **S18 Fig**). It remains unknown, why only siccanol but not fusaproliferin was detected in the Δ*fpkmt6* samples. One possible explanation is that the relatively low levels that might potentially be produced are below the limit of detection (LOD) for fusaproliferin, which exhibits a comparatively poor sensitivity in ESI-MS/MS. Next to this, four genes are involved in beauvericin biosynthesis in *F*. *fujikuroi*, encoding a NRPS (FPRN2_12971, *FpBEA1*), the ketoisovalerate reductase KivR (FPRN2_12972, *FpBEA2*), an ABC transporter (FPRN2_32324, *FpBEA3*) and a Zn2Cys6 transcription factor (FPRN2_32320, *FpBEA4*) that was shown to negatively impact beauvericin biosynthesis [24]. In line with this, only *FpBEA1* –*FpBEA3* are upregulated in *F*. *proliferatum*, while expression of *FpBEA4* is reduced in Δ*fpkmt6* compared to FpWT (**S1 Table**). It is possible that up- and downregulation of *FfBEA1*-*FfBEA3* in Δ*ffbea4* and OE::*FfBEA4*, respectively, in *F*. *fujikuroi* [24], is due to a yet unknown secondary effect. Nonetheless, expression patterns of the *FpBEA1*-*FpBEA4* were in agreement with the production levels (**S17 Fig**).

*F*. *proliferatum* is well-known to produce fumonisins [60]. Yet, fumonisins were not detected in FpWT samples although grown under low nitrogen conditions that were shown to induce fumonisin biosynthesis in the closely related *F*. *fujikuroi* [61]. In line with these results, the fumonisin BGC is not expressed in FpWT, and low expression levels are likely due to H3K27me3-mediated gene silencing as FUM genes are located in a H3K27me3-rich region (**Fig 3**). As a consequence, removal of *FpKMT6* results in an upregulation of *FpFUM1* (FPRN2_42368) the key enzyme-encoding gene involved in fumonisin biosynthesis under these conditions in Δ*fpkmt6*. Yet, next to *FpFUM1* only 5 of the 15 additional biosynthetic genes, i.e., *FpFUM21* (FPRN2_32368), *FpFUM6* (FPRN2_32367), *FpFUM7* (FPRN2_32366), *FpFUM8* (FPRN2_32365) and *FpFUM15* (FPRN2_13016) are significantly upregulated in Δ*fpkmt6* (log_2_ ≥ ±1, p-value < 0.01) thereby possibly explaining the lack of the final product also in Δ*fpkmt6* samples (**Tables 1 and S1**).

### H3K27me3 is decisive for gibberellin biosynthesis in *F*. *proliferatum* NRRL62905

**Fig 7:**
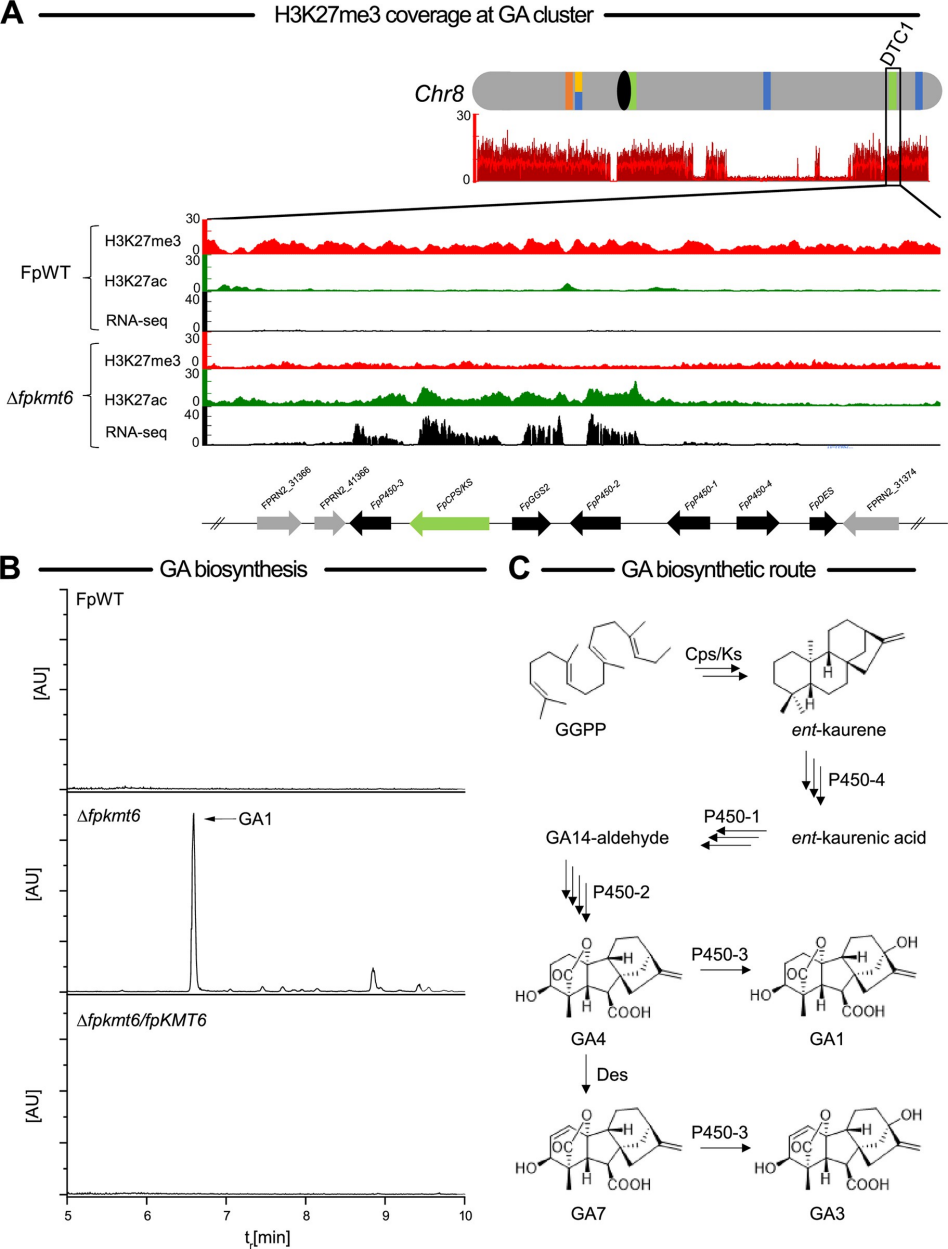
Gibberellin biosynthesis is silenced by H3K27me3 in *F*. *proliferatum*. (A) Chromosome 8 is shown in grey and centromere in black; SM key enzyme-encoding genes are indicated by bars according to the following color code: polyketide synthase (PKS), blue; non-ribosomal peptide synthetase (NRPS), orange; (sesqui-/di-/sester-/tri-/tetra-) terpene cyclase (STC/DTC/PTTS/TrTC/TeTC), light green. Below the chromosome, the H3K27me3 coverage in the *F*. *proliferatum* wild-type strain NRRL62905 (FpWT) is shown. Chromatin marks at the BGC involved in GA biosynthesis in the *F*. *proliferatum* wild-type strain NRRL62905 (FpWT) and the strain deleted for *FpKMT6* (Δ*fpkmt6*). In descending order are H3K27me3 (red), H3K27ac (green), and transcription as determined by RNA-seq (black) for FpWT and the same for Δ*fpkmt6*. Below this, the selected BGC is indicated as follows: the key enzyme-encoding gene is depicted in light green; cluster genes are shown in black, and bordering genes in grey. (B) Chemical analysis of GA biosynthesis. GA1_,_ but not GA3, was detected by targeted MS-based analysis in Δ*fpkmt6* but absent from FpWT and the complemented strain Δ*fpkmt6*/*FpKMT6*. (C) Simplified scheme of the GA biosynthetic pathway (modified from Studt and Tudzynski, 2014).

Members of the FFSC have the genetic potential to produce three classes of phytohormones, i.e., auxin, cytokinins, and gibberellins. Both auxin and cytokinins are produced in liquid cultures in FpWT, while gibberellins are not detectable [8]. Production of gibberellins (GA) is a hallmark of the rice pathogen *F*. *fujikuroi* [7]. Though the seven biosynthetic genes involved in the production of these phytohormones appear conserved in the FFSC (including *F*. *proliferatum* NRRL62905), the production of GAs is mainly restricted to *F*. *fujikuroi* with few exceptions. *F*. *proliferatum* ET1 isolated as an endophyte from orchids produces *ent*-kaurene and GAs in axenic culture though only in small amounts [8, 62]. The reasons for that are not well understood. Pseudogenization is likely responsible for the failure to detect GAs in the *F*. *proliferatum* strains D02945 and D00502 despite of expression of the respective BGC [63], while the genes are silent in other FFSC members in axenic cultures including the here used *F*. *proliferatum* wild-type strain, FpWT ([8]; this study). Intriguingly, the GA biosynthetic genes were among the most highly upregulated SM genes in Δ*fpkmt6* (Tables **1 and S1**). In line with the transcript data, GA biosynthetic genes are enriched with H3K27me3 in FpWT, and H3K27 becomes acetylated upon deletion of *FpKMT6* (**Fig 7A**). To evaluate transcript data and verify whether deletion of *FpKMT6* indeed results in detectable GA amounts, FpWT, Δ*fpkmt6* and Δ*fpkmt6*/*FpKMT6* were grown under identical, *F*. *fujikuroi* GA-inducing, conditions as for ChIP and RNA-seq for subsequent chemical analysis. In agreement with previous data [8] and transcript data, no GAs were detectable in FpWT and Δ*fpkmt6*/*FpKMT6* samples, while GAs were very well detected in the Δ*fpkmt6* strain (**Fig 7B**). The most prominent bioactive GAs are GA1, GA3, GA4 and GA7, with GA3 being the main product in *F*. *fujikuroi* GA biosynthesis [64]. However, GA1 but not GA3 was the main product in Δ*fpkmt6* (**Fig 7B**). GA biosynthesis is mediated by a seven-gene cluster, encoding a pathway-specific geranylgeranyl diphosphate synthase (FpGgs2, FPRN2_08493), the bifunctional *ent*-copalyl diphosphate synthase/*ent*-kaurene diphosphate synthase (FpCps/Ks, FPRN2_08492), four cytochrome P450 monooxygenases (FpP450-1 to FpP450-4, FPRN2_31371, FPRN2_08494, FPRN2_31367 and FPRN2_31372), and a desaturase (FpDes, FPRN2_08497). Starting from acetyl-CoA, GAs are synthesized *via* the mevalonate pathway resulting after several biosynthetic steps in the first biologically active C19 GA, GA4. Desaturation of GA4 at C1,2 by the GA4 1,2 desaturase (Des) yields GA7. In the final step, both GA4 and GA7 are converted by the C13 oxidase P450-3 to GA1 and GA3, respectively, whereby GA3 is the main product and only minor amounts of GA1 are formed in *F*. *fujikuroi* (**Fig 7C**; Studt and Tudzynski, 2014). Thus, Des activity is decisive for the proportion of GA3 to GA1 in the fungus. Indeed, *FpDES* is the only GA biosynthetic gene that is not affected by deletion of *FpKMT6* and remains silent, while the remaining six are significantly upregulated in Δ*fpkmt6* as compared to FpWT (**Fig 7A and S1 Table**), thereby explaining the absence of GA7 and GA3 in the Δ*fpkmt6* samples. Thus, FpKmt6-mediated H3K27me3 is responsible for GA-gene silencing in *F*. *proliferatum* NRRL62905.

**Fig 8:**
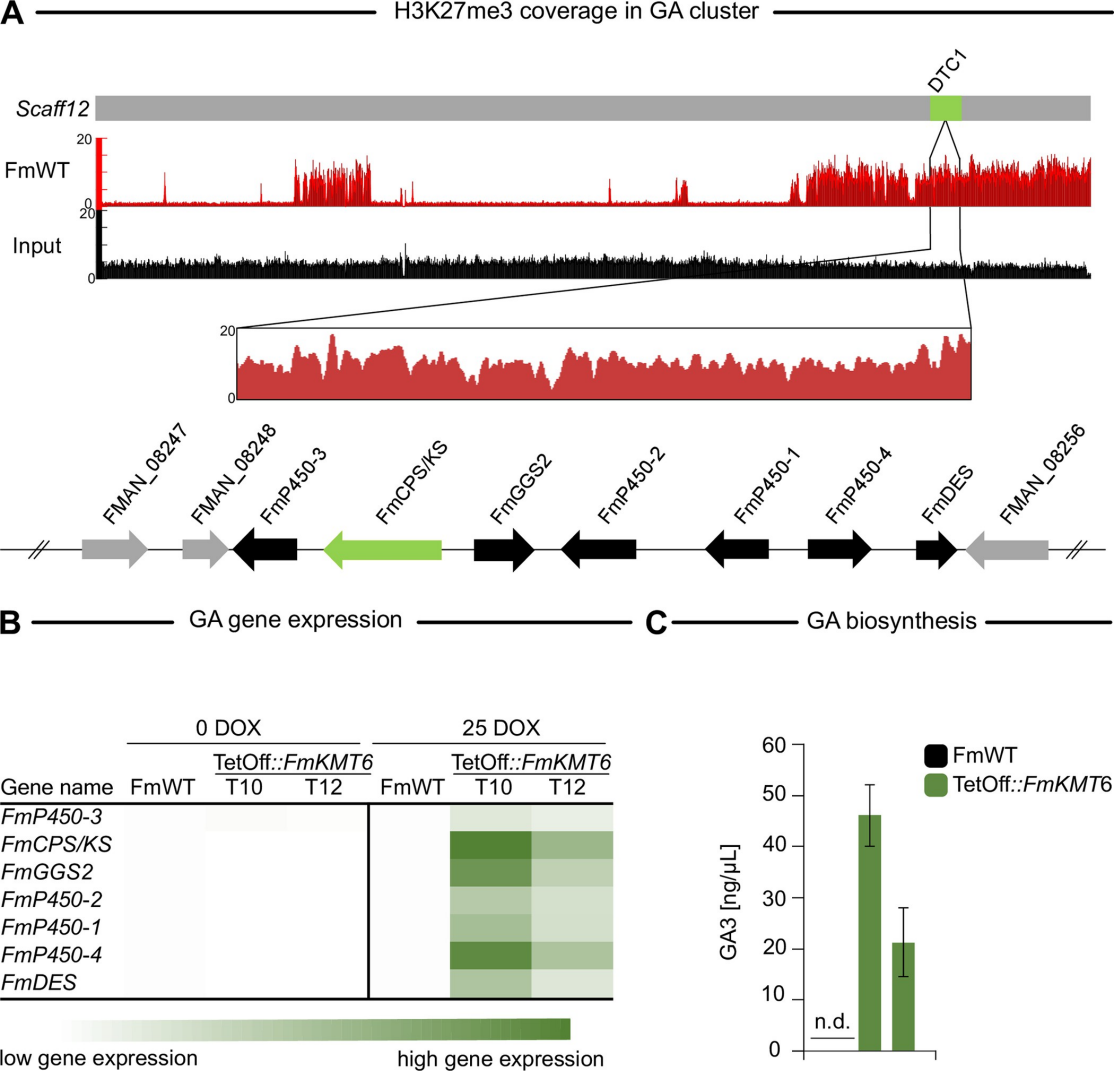
Gibberellic acid (GA) biosynthesis is also silenced by H3K27me3 in *Fusarium mangiferae*. (A) Detailed depiction of H3K27me3 coverage at the GA gene cluster (highlighted in light green) located on Scaffold12 in FmWT. Below the chromosome, the H3K27me3 coverage in the *F*. *mangiferae* wild-type strain MRC7560 (FmWT) is shown. Below this, the genes involved in GA biosynthesis are indicated as follows: the key enzyme-encoding gene is depicted in light green; cluster genes are shown in black, and bordering genes in grey. (B) Gene expression of GA biosynthetic genes in FmWT and two TetOff::*FmKMT6* mutants under non-inducing (0 DOX) and inducing (25 DOX) conditions. Fungi were cultivated under low nitrogen conditions (*F*. *fujikuroi* GA-inducing conditions). Transcript levels were determined using RT-qPCR from prior transcribed cDNA. While GA biosynthesis is silent under both conditions for FmWT, the partial reduction of H3K27me3 led to the expression of all GA biosynthetic genes. (C) GA biosynthesis in FmWT and the TetOff::*FmKMT6* knock-down strains. Fungi were cultivated under low nitrogen conditions (liquid ICI and 6 mM glutamine) for 7 days post inoculation at 30°C. Experiments were performed in triplicates. HPLC-HRMS measurement revealed that GAs are absent from cultures containing FmWT, while the TetOff::*FmKMT6* strains were very well able to produce GAs in liquid cultures.

Intrigued by these findings, we wanted to test whether H3K27me3 is also involved in GA gene silencing in another FFSC member. For this, we included *F*. *mangiferae* MRC7560 (FmWT) in our analyses. First, the genome-wide distribution of H3K27me3 was recorded *via* ChIP-seq using an H3K27me3-specific antibody and identical culture conditions as for FpWT. In line with low expression levels, GA biosynthetic genes are enriched for H3K27me3 also in FmWT (**Fig 8A**). To evaluate the relevance of H3K27me3 for GA gene regulation, downregulation of *FmKMT6* was approached using the TetOff system [65], as deletion of *FmKMT6* did not result in homokaryotic Δ*fmkmt6* strains (**S1 Fig**). At least three independent *F*. *mangiferae* mutant strains, i.e., TetOff::*FmKMT6*_T7, T8 and T10, were gained that showed correct integration of the TetOff construct as verified by diagnostic PCR (**S19A and S19B Fig**), and reduced H3K27me3 levels in western blot upon supplementation with 25 μg/mL doxycycline-hyclate (Sigma-Aldrich, DOX) (**S19C and S20 Figs**). All three TetOff::*FmKMT6* mutants showed severe growth defects when supplemented with 10 to 50 μg/mL DOX (**S19D Fig**). To test whether GA genes are affected by the downregulation of *FmKMT6* in *F*. *mangiferae*, relevant strains were grown in GA-inducing conditions (synthetic ICI with 6 mM glutamine) and GA transcript levels were analyzed and compared to FmWT. While only low and even lower GA expression was detected in FmWT and TetOff::*FmKMT6* without the addition of DOX, GA transcript levels were significantly increased upon the addition of DOX in TetOff::*FmKMT6* but remained the same in FmWT (**Fig 8B**). Chemical analysis *via* LC-MS/MS under the same culture conditions confirmed these results. Indeed, GA3 was detected in cultures inoculated with TetOff::*FmKMT6* upon supplementation with DOX, while GAs biosynthesis is still absent from FmWT (**Fig 8C**). In contrast to *F*. *proliferatum*, all seven GA biosynthetic genes are increased upon knock down of *FmKMT6* about 10- to 50-fold in *F*. *mangiferae*. Thus, it is intriguing to speculate that H3K27me3 functions as an adaptive layer for GA gene expression in *F*. *proliferatum* and *F*. *mangiferae* and possibly also other FFSC members but not *F*. *fujikuroi*. Here, the complete GA cluster is devoid of H3K27me3, which explains the fact, that *F*. *fujikuroi* can utilize these genes more frequently than other species [23]. These results again demonstrate how the chromatin landscape can shape fungal lifestyles.

## Conclusion

Packaging of DNA into chromatin is a fundamental process, which ensures balanced gene transcription. Decoration of chromatin *via* the modification of residues on histone N-termini results in an additional layer of regulation for the organism facilitating a concert of fine-tuned gene expression. The tri-methylation of histone H3 lysine 27 (H3K27me3) is known to be the hallmark of facultative heterochromatin in many fungal species and is important for cellular processes, chromosome stability or secondary metabolite (SM) gene expression. In this study, we were able to demonstrate that the H3K27me3-specific methyltransferase FpKmt6 is vital for fungal development and the key regulator for silencing fungal secondary metabolite gene expression in the FFSC member *F*. *proliferatum* NRRL62905. We further revealed that facultative and constitutive heterochromatic marks i.e., H3K27me3 and H3K9me3 are mutually exclusive but loss of H3K9me3 can be substituted by H3K27me3 spreading, confirming a crosstalk between these two histone marks also in the genus *Fusarium*. Next, we could show that loss of H3K27me3 leads to aberrant H3K36me3 patterns as well as an increase of H3K27ac in former facultative heterochromatic regions, which are enriched in BGCs. In line with the altered distribution of histone marks in the *FpKMT6* loss-of-function mutant, a predominant increase of gene transcription of former silenced BGCs was observed (56%), including the phytohormones gibberellins (GAs). GA are also not produced in the closely related FFSC member *F*. *mangiferae* but partial removal of H3K27me3 relieved repression of the GA genes and resulted in GA production. In general, the expression of GA in the genus *Fusarium* is not well understood but these results suggest that H3K27me3 gene silencing is an important regulatory layer not only for fungal secondary metabolism but also for plant colonization in this genus and further illustrates the different lifestyles of these notorious pathogens.

## Materials and methods

### Strains, media, and culture conditions

The *F*. *proliferatum* strain NRRL62905, FpWT, was used as parental strain for the generation of *FpKMT6* and *FpKMT1* deletion (Δ*fpkmt6* and Δ*fpkmtl*), and *in situ* complementation (Δ*fpkmt6*/*FpKMT6* and Δ*fpkmt1*/*FpKMT1*). The *F*. *mangiferae* MRC7560 wild-type strain, FmWT, originated in Israel and deposited in the culture collection of the Medical Research Council (MRC) (Tygerberg, South Africa) was used as parental strain for the deletion attempt (Δ*fmkmt6*), and the inducible knock-down using the tetracycline-sensitive TetOff system (TetOff::*FmKMT6*) [65]. The uracil-auxotrophic *Saccharomyces cerevisiae* FGSC 9721 (FY 834) [66] was provided by the Fungal Genetics Stock Center (Kansas State University) and used for yeast recombination cloning [67]. *Escherichia coli* strain DH5α (Invitrogen™) was used for plasmid propagation.

Strains were maintained on solid complete medium (CM, [68]). Fungal growth assays were conducted on solid CM, vegetable V8 juice (30 mM CaCO_3_, 20%, v/v, vegetable juice; Campbell Food, Puurs, Belgium), and synthetic ICI [69] medium supplemented with 6 mM glutamine (Carl Roth). Plates were inoculated either with 10 μL of a 10^5^ conidia/mL solution or agar plugs and incubated at 30°C in the dark for up to seven days. In the case of TetOff::*FmKMT6* mutant strains, solid CM was supplemented with either 0–50 μg/mL doxycycline (DOX) to induce silencing of *FmKMT6*. For genomic DNA isolation, relevant strains were grown for three days on CM covered with cellophane sheets (Folia Bringmann) at 30°C in the dark. Conidia production was assessed on solid V8. Medium was inoculated with a 5 mm^2^ agar plug each and incubated at 20°C for 7 days under the presence of 18 h light and 6 h dark (L/D) or dark conditions (D) and 70% humidity. Conidia were counted using a Neubauer improved hemocytometer under a light microscope (Carl Zeiss). Microscopy of the fungal conidia was performed using a confocal laser scanning microscope (CLSM, Leica STELLARIS 5). For fungal liquid cultivations, mycelia were pre-cultured in 100 mL Darken medium [70] in a 300 mL Erlenmeyer flask for 3 days in the dark (30°C, 180 rpm). Then, 0.5 mL of the pre-culture was transferred to 100 mL ICI media supplemented with either 6 mM or 60 mM glutamine (Carl Roth) as sole nitrogen source. In case of the TetOff strains, cultivation was performed similar as described earlier [71]. Briefly, 150 μL of Darken medium of the respective wild-type strain and mutants were transferred to 30 mL synthetic liquid ICI supplemented with 6 mM glutamine as sole nitrogen source and 25 μg/mL DOX to induce continuous silencing of *fmKMT6*. All fungal cultures were incubated on an orbital shaker at 180 rpm, 30°C for 3 days for RNA- or ChIP-sequencing as well as protein extraction in case of the TetOff mutant strains and 7 dpi for SM analysis. Protein extraction of all other mutant strains were performed with mycelia grown on solid CM at 30°C in the dark and harvested 4 dpi. For Nanopore sequencing, FpWT was grown for 3 days in liquid YPG [72].

### Plasmid construction and strain generation

Plasmids for deletion (pΔ*fpkmt6*, pΔ*fmKMT6*, and pΔ*fpkmt1*), complementation (pΔ*fpkmt6/FpKMT6* and pΔ*fpkmt1/FpKMT1*^s^), and inducible downregulation of *FmKMT6* (TetOff::*FmKMT6*) were generated using yeast-recombinational cloning as described previously [67]. Primers were obtained from Merck (Austria) and are listed in S3 Table. Fragments for cloning and subsequent transformation were amplified using a high-fidelity polymerase (Q5-polymerase, New England Biolabs or Phusion High-Fidelity DNA polymerase, Thermo Scientific). In the case of targeted gene deletion, ~1 kb upstream (5′) and downstream (3′) region of the gene of interest were amplified from FpWT gDNA using the primer pairs 5F/5R and 3F/3R, respectively, and the hygR resistance cassette amplified from the template vector pCSN44 [73] with the primer pair hphF/hphR, was used as selection marker. Complementation plasmids were generated as follows: *FpKMT6* was amplified together with ~1kb upstream (5’) region in two fragments using the primer pairs KMT6_5F/Dia_KMT6_WT_R and KMT6_F1/FpKMT6_Cil-Tgluc_R2, followed by the artificial terminator sequence, *Tgluc*, from *Botrytis cinerea* B05.10 (*BcTgluc*) and the nourseothricin resistance cassette (natR) using the primers Tgluc_F2//hphF amplified from p*ffccl1*_IL_C [74]. In the case of *KMT1*, *FpKMT1* was amplified together with ~ 1kb upstream (5’) region using the primer pair KMT1_5F/ FpKMT1_Cil-Tgluc_R, followed by *BcTgluc* and the geneticin resistance cassette (genR) with the primer pair TglucF2//hphF from pΔ*fmkmt1*/*FmKMT1*^Ces^ [28]. Amplification of the downstream (3′) region for both was performed with the respective primer pairs 3F/3R. In the case of the *in situ* inducible knock-down construct (TetOff::*FmKMT6*), the TetOff elements (Pgpda, tTA-Advanced, AFcgrA and the TetO7 sequence) coupled to the hygromycin resistance cassette was amplified from pTetOff::*FfH2A*.*Z*, using the primer pair TtrpC_R/ Tet-off_R. Next, ~1 kb of the 5’ and 3’ regions were amplified from FmWT gDNA using the primer pair KMT6_F/TetOff_KMT6_5R and TetOff_KMT6_3F/KMT6_3R, respectively. The *Saccharomyces cerevisiae* FY834 strain was transformed with the respective PCR fragments, the resistance cassettes and the *Eco*RI/*Xho*I-digested shuttle vector pRS426, yielding pΔ*fpkmt6*/*FpKMT6*, Δ*fpkmt1*/*FpKMT1* and TetOff::*FmKMT6* (**S3A, S8A and S19A Figs**). Correctness of the complementation and the silencing plasmids was verified by sequencing (LGC Genomics, Germany).

Fungal transformations were performed as described previously [32]. For gene deletion, linear fragments were amplified from pΔ*fpkmt6*, pΔ*fmkmt6* and pΔ*fpkmt1* with the respective primer pairs 5F//3R (S1 Table), using a proof-reading polymerase (Q5-polymerase, New England Biolabs or Phusion Flash High-Fidelity PCR Master Mix, Thermo Fisher Scientific). In the case of complementation, 10 μg each of pΔ*fpkmt6/FpKMT6* and pΔ*fpkmt1/FpKMT1* were digested with *Xba*I/*Xho*I prior to transformation (**S3A and S8A Figs**). Transformation of the TetOff::*FmKMT6* construct was approached using 10 μg *Aan*I/*Eco*105I digested plasmid DNA (pDNA, **S19A Fig**). Fungal transformants were selected on regeneration medium supplemented with the appropriate antibiotic i.e., hygromycin B (Merck Millipore), geneticin (Fermtech Garching) or nourseothricin (Jena Bioscience). Homologous integration events of Δ*fpkmt6* and Δ*fmkmt6* was verified by diagnostic PCR using GoTaq G2 DNA Polymerase (Promega) and the primer pairs dia_kmt6_5’/trpC-T and dia_kmt6_3’/trpC-P, and absence of the wild-type gene was analyzed using the primer pairs dia_KMT6_WT_F/R (**S1B, S1C and S2B Figs and S3 Table**). In the case of Δ*fpkmt1*, dia_kmt1_5’/trpC-T and dia_kmt1_3’/trpC-P proved homologous integration, and absence of the wild-type gene was verified using the primer pair dia_KMT1_WT_F/R (**S6 Fig and S3 Table**). Successful complementation of Δ*fpkmt6* (**S3 Fig**) with the respective wild-type gene, *FpKMT6*, was verified with the primer pairs dia_KMT6_5’/trpC-T (absence of hphR), dia_KMT6_3’/trpC-P2 and dia_KMT6_WT_F/dia_KMT6_WT_R (presence of natR and *FpKMT6*, respectively. In the case of Δ*fpkmt1*, attempts to complement the deletion strain Δ*fpkmt1* by *in situ* integration of *FpKMT1* failed in our hands, but several transformants that showed ectopic integration of the complementation fragment were gained as verified with the primer pairs KMT1_dia_WT_F/R (presence of *FpKMT1*), dia_kmt1_5’/KMT1_dia_WT_R and dia_kmt1_3’/genR_R (would show *in situ* integration, **S8 Fig**). Complemented strains were additionally verified by reverse transcriptase-quantitative PCR (RT-qPCR, **S3C and S8C Figs**).

### Standard molecular techniques

Yeast pDNA was extracted with the GeneJET Plasmid Miniprep Kit (Thermo Fisher Scientific) according to the manufacturer’s protocol and directly used as templates for PCR in the case of the targeted gene deletion. In the case of complementation, yeast pDNA was re-transformed in *E*. *coli* DH5α (Invitrogen) according to the manufacturer. Subsequent extraction of pDNA was again performed with the GeneJET Plasmid Miniprep Kit (Thermo Fisher Scientific. Genomic DNA (gDNA) for subsequent PCRs was extracted from lyophilized, ground material as described elsewhere [75]. For gene expression analysis, RNA was isolated from lyophilized and ground mycelium with the RNA reagent TRIzol (Thermo Fisher Scientific) according to the manufacturer’s instructions. For cDNA synthesis, 1 μg RNA was treated with DNaseI (Thermo Fisher Scientific) and transcribed into cDNA using LunaScript RT SuperMix Kit (NEB). For subsequent RT-qPCR analysis the iTaq Universal SYBR Green Supermix (Bio-Rad) was used and cDNA was quantified on an iCycler iQ Real-Time PCR System (Bio-Rad). In all cases the primer efficiency was kept between 90 and 110%, Ct values greater than 32 were taken as not expressed. Results were calculated according to the ΔΔCt [76]. Expression of all tested genes were normalized to the expression of actin (FPRN2_04233), Glyceraldehyde-3-phosphate dehydrogenase (GPD, FPRN2_25921), and β-tubulin (FPRN2_07708). All primers used are listed in **S3 Table**. Western blot analyses was performed as described previously [28]. For western blot analysis roughly 25–50 μg of total protein extract was separated on a SDS gel and the following primary antibodies were used for subsequent probing: anti-H3 C-Term (Active motif, 91299), anti-H3K9me3 (Active motif, 39161 / Abcam, 8988), anti-H3K27me3 (Active motif, 39155), and anti-H3K27ac (Cell Signaling, D5E4 XP), and anti-rabbit HRP-conjugated secondary antibody (Jackson ImmunoResearch) was used for subsequent detection of chemiluminescence signals. Membranes were developed with Clarity ECL Western Substrate (Bio-Rad) and visualized with a ChemiDoc XRS (Bio-Rad) system. Subsequent densitometric analyses of western blot signals were performed using the ImageJ software. Signals were normalized to the histone H3 C-term signal and the wild-type signal ratio was set to 100% for referencing.

### Chemical analyses

Supernatants for chemical analyses were retrieved from fungi-inoculated 7-day-old liquid ICI cultures supplemented either with 6 mM or 60 mM glutamine. Detection of siccanol in FpWT, Δ*fpkmt6* and Δ*fpkmt6*/*FpKMT6*^Cis^ as well as GA_3_ in samples from *F*. *mangiferae* were analyzed as previously described [77]. For all other samples, supernatants were filtered through the 0.2 μm syringe filters (RC membrane, 15 mm, non-sterile, PP housing, luer/slip, Phenomenex, Aschaffenburg, Germany). An aliquot of 100 μL filtrate was diluted 1:1 with acetonitrile (MeCN), the samples were stored at 4°C overnight, filtered a second time, and directly used for screening analysis. Bikaverin, fusaric acid, and beauvericin were obtained from Merck (Darmstadt, Germany), Gibberellins (GAs) GA1, GA3, GA4 and GA7 were obtained from Serva (Heidelberg, Germany), fumonisins B1, B2, and fusarin C were obtained as described in previous work [78] all in analytical grade.

For the targeted screening an LC-MS/MS method was developed. The chromatographic separation and subsequent MS/MS analysis was achieved with a 1260 LC system (Agilent, Waldbronn, Germany), coupled to a QTRAP 5500 mass spectrometer equipped with a Turbo V Ion Source (SCIEX, Darmstadt, Germany). Data acquisition and subsequent data analysis was done with the Analyst 1.6.2. software (SCIEX, Darmstadt, Germany). Chromatographic separation was performed on a Nucleodur C18 Gravity SB column (100 mm * 2 mm i.d., particle size 3 μm) equipped with a 4 * 2 mm i.d. guard column of the same material (Macherey-Nagel, Düren, Germany). The gradient started with 10% MeCN + 0.1% FA (solvent A) and 95% H_2_O + 0.1% FA (solvent B) for 1.5 min and a flow rate of 0.50 mL/min at 40°C column oven temperature. Within 15.5 min, A was increased linearly to 100%. For the next 1.5 min, A was held at 100%. Equilibration was performed for 3 min. Electrospray ionization was performed in both positive mode at a source voltage of 4.5 kV, and in negative mode at -4.5 kV. Source temperature was set to 450°C; curtain gas flow to 35 psi, nebulizer gas to 35 psi, heater gas to 45 psi. Both declustering potential (DP) and collision energy voltage (CE) were optimized along with the analyte specific transitions and measurement modes and are given in S4 Table. For qualitative detection of the formation of the target compounds, precursor ion scans were implemented to the LC-MS/MS method based on the fragmentation pattern of the respective analyte. Additionally, a standard solution was prepared containing all analytes at a concentration of 50 μg/mL, which was measured before sample measurement was commenced and after completion of the sample sequence. In all cases, obtained results were normalized to the biomass of the respective strains to account for growth differences of the different strains.

### Nanopore sequencing and new assembly

High molecular weight DNA was extracted from ground lyophilized mycelium using Genomics Buffer Set (Qiagen) and purified using QIAGEN Genomic-Tips 20/G as described previously [45]. Small DNA fragments were removed from the sample with Circulomics Short Read Eliminator XS in accordance with manufacturers recommendations prior to sequencing on a MinION R9.4.1 flow cell using the ligation sequencing protocol (SQK-LSK109) from Oxford Nanopore Technologies. Basecalling, demultiplexing, and adaptor removal were performed with Guppy v5.0.12 [79] in GPU mode using the r941_min_sup_g507 model. The reads were filtered to a minimum length of 20 kb and a minimum quality of 80 (Q7) using Filtlong v0.2.0 [80] followed by assembling of the reads as described previously (Petersen et al., 2022) with the software versions: Minimap2 v2.17 [81], Miniasm v0.3 [82]. Polishing was performed by Racon v1.3.3 [83] and Medaka v1.4.3 (Oxford Nanopore Technologies, 2018). An identity between utg000012l and utg000004l was observed and BLAT v37x1 [84] was used to calculate the overlap; thus, the first 878,742 pb from utg000012l were then added to utg000004l on the 5’ end creating the new utg000004l. The fungal telomeric repeat sequence of TTAGGG/CCCTAA was used to identify telomeric region in the assembly of FpWT as described previously [45]. The genome sequence data is available on NCBI Genbank CP128300-CP128312.

### RNA sequencing, mapping and quantification

Total RNA for subsequent sequencing was extracted using TRIzol Reagent as described above, and sent to the Vienna BioCenter (Vienna, Austria) for quality control, library preparation and sequencing. Experiments were performed in duplicates. Library prep and sequencing was performed using poly-A enrichment kit (NEB) and Nextera Library prep kit. 50 bp single end sequencing was performed using a HiSeq v4 Illumina sequencer. Obtained sequences were de-multiplexed, quality controlled, filtered using trimmomatic 0.36 [85] and mapped on the new genome assembly. Mapping was performed using BWA [86] and reverse transcripts were counted using python script HTSeq [87]. Normalization and statistics were done using R/Bioconductor and the limma and edgeR packages, using mean-variance weighting (voom) and TMM normalisation [88]. A significance cut-off of p < 0.01 and differential expression of +/-1 (2-fold) was applied for analysis. Transcription levels are log2 read counts per kilobase of exon per million library reads (RPKM). The RNA-seq data has been submitted to NCBI’s Gene Expression Omnibus [89] and are accessible through GEO Series accession number GSE235901. GO annotation done using PANNZER2 [90] and processed using R library “GOstats”, “GSEABase”; GO term reduction was done using library “rrvgo”. Only GO-terms enriched in the selected gene sets with p < 0.05 were consider for further investigation.

### Chromatin immunoprecipitation and ChIP-sequencing

Chromatin immunoprecipitation (ChIP) was performed essentially as described [23, 74]. The following antibodies were used for immunoprecipitation: anti-H3K9me3 (Active motif, 39161), anti-H3K27me3 (Active motif, 39155), anti-H3K27ac (Cell Signaling, D5E4-XP(R)), H3K36me3 (Abcam, 9050). Experiments were performed at least in biological duplicates, and input controls were added for each strain. Obtained DNA was sent to the Vienna BioCenter (Vienna, Austria) for library preparation and sequencing. Paired-end sequencing was performed on HiSeq v4 Illumina sequencer and quality filtering, trimming and mapping was performed as described for RNA-seq experiments. Quantification of mapping in specific regions around genes were performed in R using the GenomicRanges Biostrings [91, 92]. Regions of interest and ChIP coverage were determined as follows: For H3K27me3 the maximum coverage in the region 100 bp upstream and 1000 bp downstream of ATG of each gene, coverages above 4 were considered as positives; for H3K27ac the max coverage in the region 2000 bp upstream and 100 bp downstream with a threshold of 6; for H3K36me3 the region 1000bp upstream and 0 bp downstream and minimum coverage of above 2 were considered positive; and for H3K9me3 peaks above a height of 2 were considered positives, peaks present in both (Δ*fpkmt6* and FpWT) were excluded only peaks emerging in kmt6 were considered and genes within regions +/- 10kb and +/- 50 kb around them were indicated. Peak-calling was done in R functions used are available via github (https://github.com/symbiocyte/MNase). The ChIP-seq data is available under BioProject PRJNA986687.

## Supporting information

S1 Fig Loss of FmKmt6 is lethal in *Fusarium mangiferae*. (A) *FmKMT6* deletion strategy in *F*. *mangiferae* MRC7560 (FmWT). Primers used for diagnostic PCRs are shown as arrows. (B) Verification of homologous recombination of the hygromycin resistance cassette (hygR) in the native *FmKMT6* locus. Homologous integration was verified by the presence of the upstream region (5’ flank) and downstream region (3’ flank). (C) Presence of the 5’ and 3’ flank as well as the wild-type gene after single spore isolation. As positive (+) control FmWT gDNA was used, while sterile IonEx was used as a negative control (C-). The 1 kb Plus DNA ladder (NEB) was used as a size marker.
(TIF)

S2 Fig Generation of viable *FpKMT6* deletion strains in *Fusarium proliferatum*. (A) *FpKMT6* deletion strategy in *F*. *proliferatum* NRRL62905 (FpWT). Primers used for diagnostic PCRs are shown as arrows. Probe used for Southern blotting is indicated as red bar. (B) Verification of homologous recombination of the hygromycin resistance cassette (hygR) in the native *FmKMT6* locus. Homologous integration was verified by the presence of the upstream region (5’ flank) and downstream region (3’ flank). As positive (+) control FpWT gDNA was used. The 1 kb Plus DNA ladder (NEB) was used as a size marker. (C) For the verification of a single integration event of the hygR cassette in the Δ*fpkmt6* mutants Southern blot was performed. The left panel shows the digested DNA before hybridization of the probe. On the right panel the visualized probes for FpWT (2.3 kb) and the *FpKMT6* deletion strains (3.7kb) are shown at the correct size.
(TIF)

S3 Fig *In situ* complementation of Δ*fpkmt6* with *FpKMT6* in *Fusarium proliferatum*. (A) *FpKMT6* complementation strategy. For complementation the Δ*fpkmt6* T1 deletion mutant was arbitrarily chosen. *Xho*I and *Xba*I were used for plasmid linearization prior to transformation. Restriction sites are depicted in the plasmid map. Primers used for verification of successful homologous recombination are indicated in the scheme below the plasmid map. (B) Complementation was achieved by *in situ* integration of the native *FpKMT6* wild-type gene of FpWT into Δ*fpkmt6* T1 using a nourseothricin resistance cassette (natR). Correct re-integration of the native *FpKMT6* gene in Δ*fpkmt6/FpKMT6* strains was verified by diagnostic PCR. Here, presence of the upstream region (5’), downstream region (3’) and wild-type (WT) gene was tested. As negative control (5’ and 3’) gDNA of *F*. *proliferatum* NRRL62905 (FpWT) was used, while FpWT served as positive control for wild-type gene amplification. Water served as a negative control and as size marker the 1 kb Plus DNA ladder (NEB) was used. (C) Complemented Δ*fpkmt6/FpKMT6* strains were tested by RT-qPCR to verify reconstitution of *FpKMT6* expression. *FpKMT6* gene expression was restored in two independent transformants, while it is absent from the Δ*fpkmt6* strain. RE, relative expression.
(TIF)

S4 Fig Uncropped western blot images of *Fusarium proliferatum* (FpWT), Δ*fpkmt6* deletion and Δ*fpkmt6*/*FpKMT6* complementation strains. For analysis, the following antibodies were used: H3K27me3- (AM39155) and H3K27ac-specific antibody (D5E4-XP(R)) as well as a H3 control (AM91299).
(TIF)

S5 Fig Impact of *FpKMT6* deletion on conidiation in *Fusarium proliferatum*. Conidia of *F*. *proliferatum* NRRL62905 (FpWT) and three independent Δ*fpkmt6* strains were quantified using a Neubauer improved counting chamber. Experiments were performed in biological triplicates, mean values are shown. While the overall conidia count was decreased significantly, the conidia/mm^2^ count was wild type-like when related to the radial hyphal growth of the respective strains.
(TIF)

S6 Fig Generation of viable *FpKMT1* deletion strains in *Fusarium proliferatum*. (A) Gene deletion strategy of Δ*fpkmt1* mutants in *F*. *proliferatum* NRRL62905 (FpWT). Primers used for diagnostic PCRs are shown as arrows in the schemata. (B) Verification of the homologous integration with the hygromycin resistance cassette (hygR) in the native *FpKMT1* locus. The homologous integration was verified by the presence of the upstream region (5’), downstream region (3’ flank) as well as the absence of the native *FpKMT1* gene. FpWT gDNA was used as negative (5’, 3’) as well as positive control (native wild-type gene). Sterile IonEx was used as a negative control (-). The 1 kb Plus DNA ladder (NEB) was used as a size marker. (C) Western blot analysis of FpWT and *FpKMT1* deletion strains using a H3K9me3- (AM 39161/ab8898) and H3K27me3-specific antibody (AM39155) as well as a H3 control (AM91299). For quantification, a densitometric analysis was performed and the respective wild-type strain was arbitrarily set as 1.
(TIF)

S7 Fig Uncropped western blot images of Fusarium proliferatum (FpWT) and Δfpkmt1 deletion strains. For analysis following antibodies were used: H3K9me3- (AM39161/ab8898) and H3K27me3-specific antibody (AM39155) as well as a H3 control (AM91299).
(TIF)

S8 Fig *In situ* complementation of Δ*fpkmt1* with *FpKMT1* in *Fusarium proliferatum*. (A) *FpKMT1* complementation strategy. For complementation, the Δ*fpkmt1* T4 deletion mutant was arbitrarily chosen. *Xho*I and *Xba*I were used for plasmid linearization prior to transformation. Restriction sites are depicted in the plasmid map. Primers used for verification of successful homologous recombination are indicated in the scheme below the plasmid map. (B) Complementation was achieved by *in situ* integration of the native *FpKMT6* wild-type gene of FpWT into Δ*fpkmt6* T1 using a geneticin resistance cassette (genR). Correct re-integration of the native *FpKMT1* gene in Δ*fpkmt1/FpKMT1* strains was verified by diagnostic PCR. Here, presence of the upstream region (5’), downstream region (3’) and wild-type (WT) gene was tested. As negative control (5’ and 3’) gDNA of *F*. *proliferatum* NRRL62905 (FpWT) was used, while FpWT served as positive control for wild-type gene amplification. Water served as a negative control. As size marker 1 kb Plus DNA ladder (NEB) was used. (C) Complemented Δ*fpkmt1/FpKMT1* strains were tested by RT-qPCR to verify reconstitution of *FpKMT1* expression. *FpKMT1* gene expression was restored in three independent transformants, while it is absent from the Δ*fpkmt1* strain. RE, relative expression.
(TIF)

S9 Fig Functional characterization of *FpKMT1* deletion strains. (A) Radial hyphal growth of the *F*. *proliferatum* NRRL62905 wild-type strain (FpWT) as well as the *FpKMT1* deletion (Δ*fpkmt1*) and complementation (Δ*fpkmt1*/*FpKMT1*) strains on different growth media. CM, V8 (complete media) and ICI (minimal medium) were inoculated with an agar plug and incubated for 5 days post inoculation at 30°C in the dark. Experiments were performed in biological triplicates. Hyphal growth of FpWT on the respective media was arbitrarily set to 1. Mean values and standard deviations are shown in the diagram. (B) Conidiation assay using FpWT, the *FpKMT1* deletion strain and Δ*FpKMT1*/*FpKMT1* complementation strain. Conidiation was induced on V8 and samples were incubated for 7 days under a light / dark cycle (18h/6h), 20°C and 70% humidity. Experiments were performed in triplicates. Conidia production of FpWT was arbitrarily set to 1. Mean values and standard deviations are shown in the diagram.
(TIF)

S10 Fig H3K9me3 peaks are lost in the *FpKMT1* deletion strain. The twelve chromosomes are shown in grey i.e., Chr1 –Chr12, and centromeres are shown in black; SM key enzyme-encoding genes are indicated by bars according to the following color code: polyketide synthase (PKS), blue; non-ribosomal peptide synthetase (NRPS), orange; (sesqui-/di-/sester-/tri-/tetra-) terpene cyclase (STC/DTC/PTTS/TrTC/TeTC), light green; dimethylallyl tryptophan synthase (DMATS), red; putative fungal RIPPs, light brown; cyclodipeptide synthase (CDPS), dark brown. Pseudogenes are highlighted with an ψ; Genome-wide distribution of H3K27me3 and H3K9me3, present in the wild-type strain (FpWT) as well as H3K9me3 in the Δ*fpkmt1* strain are depicted beneath the chromosome arms. Input control of Δ*fpkmt1* verifies presence of genomic DNA.
(TIF)

S11 Fig Gain of a few H3K9me3 peaks in Δfpkmt6 shown in triplicates. Affected chromosomes are shown in grey and centromeres are shown in black; SM key enzyme-encoding genes are indicated by bars according to the following color code: polyketide synthase (PKS), blue; non-ribosomal peptide synthetase (NRPS), orange; (sesqui-/di-/sester-/tri-/tetra-) terpene cyclase (STC/DTC/PTTS/TrTC/TeTC), light green; dimethylallyl tryptophan synthase (DMATS), red; putative fungal RIPPs, light brown; cyclodipeptide synthase (CDPS), dark brown. Pseudogenes are highlighted with an ψ; Genome-wide distribution of H3K27me3 and H3K9me3, present in the wild-type strain (FpWT) as well as H3K9me3 in the Δ*fpkmt6* strain in two biological replicates are depicted beneath the chromosome arms.
(TIF)

S12 Fig H3K27me3 and H3K9me3 peaks gained in Δfpkmt1 and Δfpkmt6, respectively, shown in biological replicates. Affected chromosomes are shown in grey and centromeres are shown in black; SM key enzyme-encoding genes are indicated by bars according to the following color code: polyketide synthase (PKS), blue; non-ribosomal peptide synthetase (NRPS), orange; (sesqui-/di-/sester-/tri-/tetra-) terpene cyclase (STC/DTC/PTTS/TrTC/TeTC), light green; dimethylallyl tryptophan synthase (DMATS), red; putative fungal RIPPs, light brown; cyclodipeptide synthase (CDPS), dark brown. Genome-wide distribution of H3K27me3 and H3K9me3, present in the wild-type strain (FpWT) as well as H3K27me3 in the Δ*fpkmt1* strain in two biological replicates are depicted beneath the chromosome arms.
(TIF)

S13 Fig H3K27me3 substitutes for the loss of H3K9me3 in the FpKMT1 deletion strain. The twelve chromosomes are shown in grey i.e., Chr1 –Chr12, and centromeres are shown in black; SM key enzyme-encoding genes are indicated by bars according to the following color code: polyketide synthase (PKS), blue; non-ribosomal peptide synthetase (NRPS), orange; (sesqui-/di-/sester-/tri-/tetra-) terpene cyclase (STC/DTC/PTTS/TrTC/TeTC), light green; dimethylallyl tryptophan synthase (DMATS), red; putative fungal RIPPs, light brown; cyclodipeptide synthase (CDPS), dark brown. Pseudogenes are highlighted with an ψ; Genome-wide distribution of H3K27me3 and H3K9me3, present in the wild-type strain (FpWT) as well as H3K27me3 in the Δ*fpkmt1* strain are depicted beneath the chromosome arms.
(TIF)

S14 Fig Reduced H3K36me3 in previous H3K27me3 regions upon deletion of *FpKMT6*. The selected chromosomes are shown in grey i.e., Chr4 and Chr11, and centromeres are shown in black; SM key enzyme-encoding genes are indicated by bars according to the following color code: polyketide synthase (PKS), blue; non-ribosomal peptide synthetase (NRPS), orange; (sesqui-/di-/sester-/tri-/tetra-) terpene cyclase (STC/DTC/PTTS/TrTC/TeTC), light green; dimethylallyl tryptophan synthase (DMATS), red. Pseudogenes are highlighted with an ψ; Genome-wide distribution of H3K27me3 and H3K36me3, present in the wild-type strain (FpWT) as well as H3K36me3 in the Δ*fpkmt6* strain are depicted beneath the chromosome arms.
(TIF)

S15 Fig Comparison of H3K36me3 occupancy on promoter regions (A) with presence of H3K27me3 and (B) with gene transcription in the *F*. *proliferatum* wild-type strain. (A) Venn diagram of number of genes with H3K27me3 labeling (red) and with H3K36me3 in their promoter region (blue). Genes allocated with both histone marks are written in the intersection. (B) Different H3K36me3 occupancy in promoter regions between Δ*fpkmt6* and FpWT (positive numbers represent higher H3K36me3 occupancy in Δ*fpkmt6* (and *vice versa*) in respect to differential transcription between Δ*fpkmt6* and FpWT. Red and blue dots represent euchromatic and H3K27me3 labeled genes, respectively.
(TIF)

S16 Fig Biomass accumulation during liquid cultivation of Δ*fpkmt6* compared to the *F*. *proliferatum* wild-type strain. Fungal strains were cultivated in liquid ICI supplemented either with low amounts of nitrogen (6 mM glutamine) or high amounts of nitrogen (60 mM glutamine) for 7 days. The dry weight of freeze-dried fungal mycelium was quantified, and respective strains were grown in triplicates.
(TIF)

S17 Fig Changes in chemical profiles upon loss of FpKmt6. The *Fusarium proliferatum* NRRL62905 wild-type strain (FpWT), the *FpKMT6* deletion (Δ*fpkmt6*) and complemented (Δ*fpkmt6/FpKMT6*) complemented strains were grown in exactly the same conditions as for RNA-seq (and ChIP-seq) i.e., in liquid ICI low nitrogen conditions (6 mM glutamine) for 7 days at 30°C. Fungal supernatants were quantified using LC-MS/MS. Quantities of known SMs are illustrated as heatmaps. Determined quantities are normalized to the biomass formation (Area/g dry weight). 0 denotes for not detected. Experiments were performed in biological triplicates and technical duplicates.
(TIF)

S18 Fig Fusaproliferin biosynthesis is relieved upon loss of H3K27me3 in Δ*fpkmt6*. Chromosome 4 is shown in grey and centromeres are shown in black; SM key enzyme-encoding genes are indicated by bars according to the following color code: polyketide synthase (PKS), blue; non-ribosomal peptide synthetase (NRPS), orange; (sesqui-/di-/sester-/tri-/tetra-) terpene cyclase (STC/DTC/PTTS/TrTC/TeTC), light green. Below the chromosome the H3K27me3 coverage in the *F*. *proliferatum* wild-type strain NRRL62905 (FpWT) is shown. Localization of the fusaproliferin BGC (FUP) is boxed, and relevant chromatin marks (H3K27me3 and H3K27ac) allocated with this region in FpWT and in a strain deleted for *FpKMT6* (Δ*fpkmt6*) are zoomed in. In descending order are H3K27me3 (red), H3K27ac (green), Input (grey) and transcription as determined by RNA-seq (black) for FpWT and Δ*fpkmt6*. Below this, the selected BGC is indicated as follows: the key enzyme-encoding gene is depicted in yellow; cluster genes are shown in black. Loss of H3K27me3 results in H3K27ac accompanied by the transcription of the otherwise silent FUP BGC.
(TIF)

S19 Fig Generation and functional verification of a *FmKMT6* knock-down *via* the inducible TetOff system in *Fusarium mangiferae*. (A) Plasmid map of the TetOff::*FmKMT6* construct. Before fungal transformation the plasmid was linearized with the restriction enzymes *Eco*105 and *Aan*I. (B) Verification of the *in-locus* integration of the knock-down construct and absence of the native wild-type gene. The upstream (5’) and downstream (3’) regions were amplified using the primer pairs dia_KMT6_5’//trpC-T and dia_KMT6_3’//trpC-P2, respectively. Absence of the wild-type gene was verified with the primers kmt6_WT_diaF2 and kmt6_WT_diaR2. FmWT genomic DNA was used as a negative (5’/3’) and positive control (wild-type gene). As negative control (-) sterile IonEx was used. (C) Verification of *FmKMT6* knock-down *via* western blot analysis from FmWT and TetOff::*FmKMT6* strains using an H3K27me3-specific antibody (AM39155) and a H3 control (AM91299). For quantification, a densitometric analysis was performed and the respective wild-type strain was arbitrarily set as 1. (D) Radial hyphal growth assay using FmWT and the TetOff::*FmKMT6* strains on ICI (6 mM glutamine) supplemented with 0–50 μg/mL DOX. Plates were inoculated with an agar plug and incubated for 4 days at 30°C in the dark. Experiments were performed in biological triplicates.
(TIF)

S20 Fig Uncropped western blot images of *Fusarium mangiferae* (FmWT) and TetOff::*FmKMT6* knock-down strains. For analysis, the following antibodies were used: H3K27me3-specific antibody (AM39155) and a H3 control (AM91299).
(TIF)

S1 Table Transcriptome of Δ*fpkmt6* in comparison to the *F*. *proliferatum* wild-type strain (FpWT). Columns Fpkmt6_1, Fpkmt6_2, FpWT_1 and FpWT_2 represent raw counts of RNA-seq experiment; Fpkmt1 and FpWT represent RPKM (log2 of reads per kilobase per million reads); kmt6…WT represents the log2 difference and kmt6…WT.p the p-value of this difference being zero.
(XLSX)

S2 Table Gene Ontology (GO) enrichment analysis of H3K27me3 labeled genes of biological processes. Tab GO_H3K27me3_BPOverrep represents the overrepresentation of all H3K27me3 labeled genes, tab GO_H3K27me3_BPOverrep_and upreg represents all H3K27me3 associated genes that are upregulated in the in Δ*fpKMT6* strain. Cloumns: GO number, Pvalue: p-value of being not overrepresented, OddsRatio: odds ration of being overrepresented, ExpCount: expected number of genes found in the selected gene-sets by chance, Count Size: actual number of genes in the selected gene-sets with the respective GO-term and Term: written GO term associated with the GO number.
(XLSX)

S3 Table Primers used in this study. Introduced overhangs required for yeast recombinational cloning are written in small letters.
(DOCX)

S4 Table Applied mass spectrometer and source parameters. Retention time (t_R_) in minutes [min], electrospray ionization (ESI) mode, mass-to-charge-ratio (*(m/z)*), declustering potential (DP) and collision energy (CE) both in volt [V], protonated adduct (M+H), deprotonated adduct (M-H), ammonium adduct (M+NH_4_) quantifier m/z is marked with *.
(DOCX)
