# Supplementary figures and images for: H3K27me3 is vital for fungal development and secondary metabolite gene silencing, and substitutes for the loss of H3K9me3 in the plant pathogen Fusarium proliferatum
Source: PLoS Genet. 2024 Jan 2;20(1):e1011075. doi: 10.1371/journal.pgen.1011075 (PMC10786395; doi:10.1371/journal.pgen.1011075)

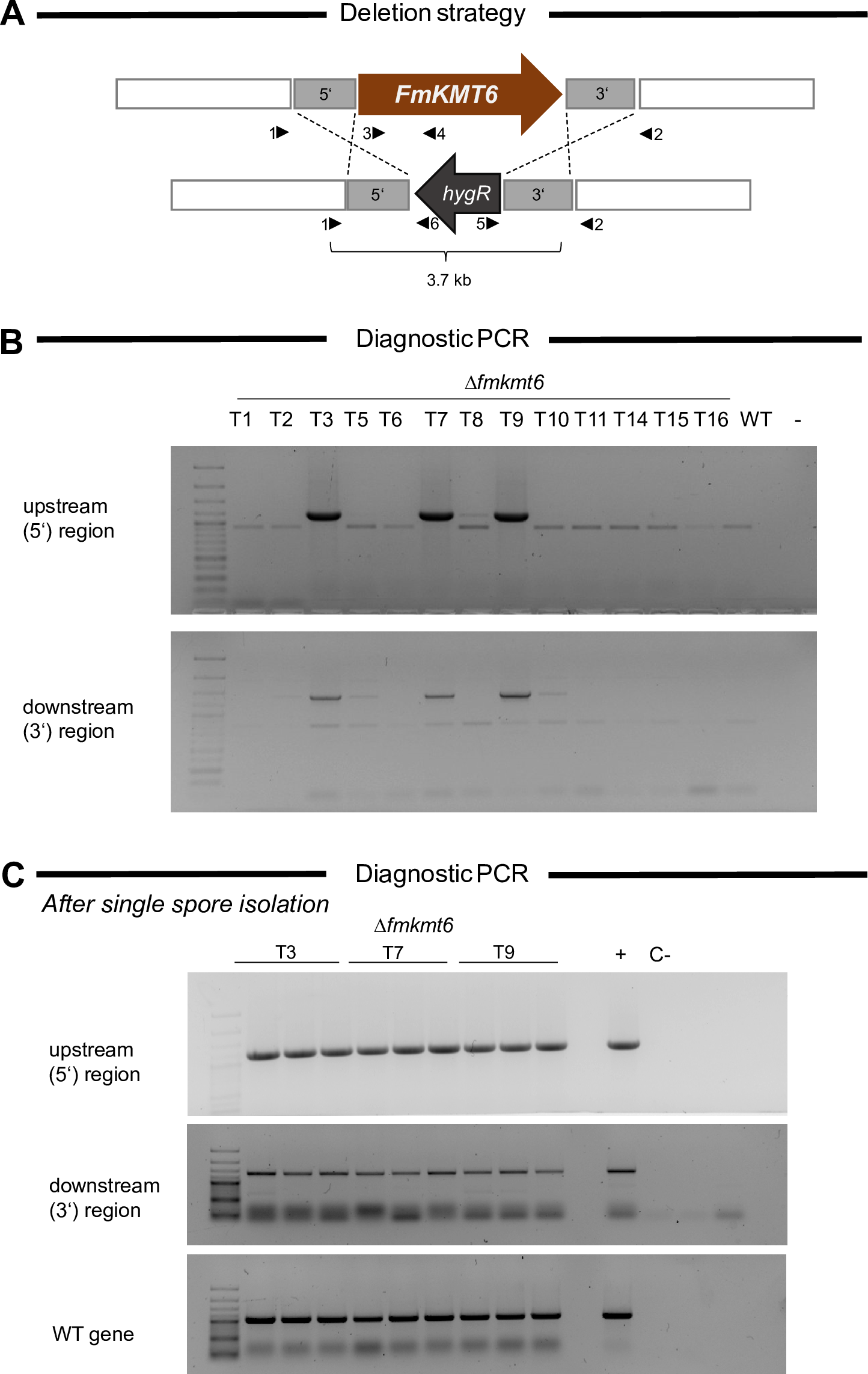

Supplement: S1 Fig — (A) FmKMT6 deletion strategy in F. mangiferae MRC7560 (FmWT). Primers used for diagnostic PCRs are shown as arrows. (B) Verification of homologous recombination of the hygromycin resistance cassette (hygR) in the native FmKMT6 locus. Homologous integration was verified by the presence of the upstream region (5’ flank) and downstream region (3’ flank). (C) Presence of the 5’ and 3’ flank as well as the wild-type gene after single spore isolation. As positive (+) control FmWT gDNA was used, while sterile IonEx was used as a negative control (C-). The 1 kb Plus DNA ladder (NEB) was used as a size marker. (TIF) [file pgen.1011075.s001.tif]

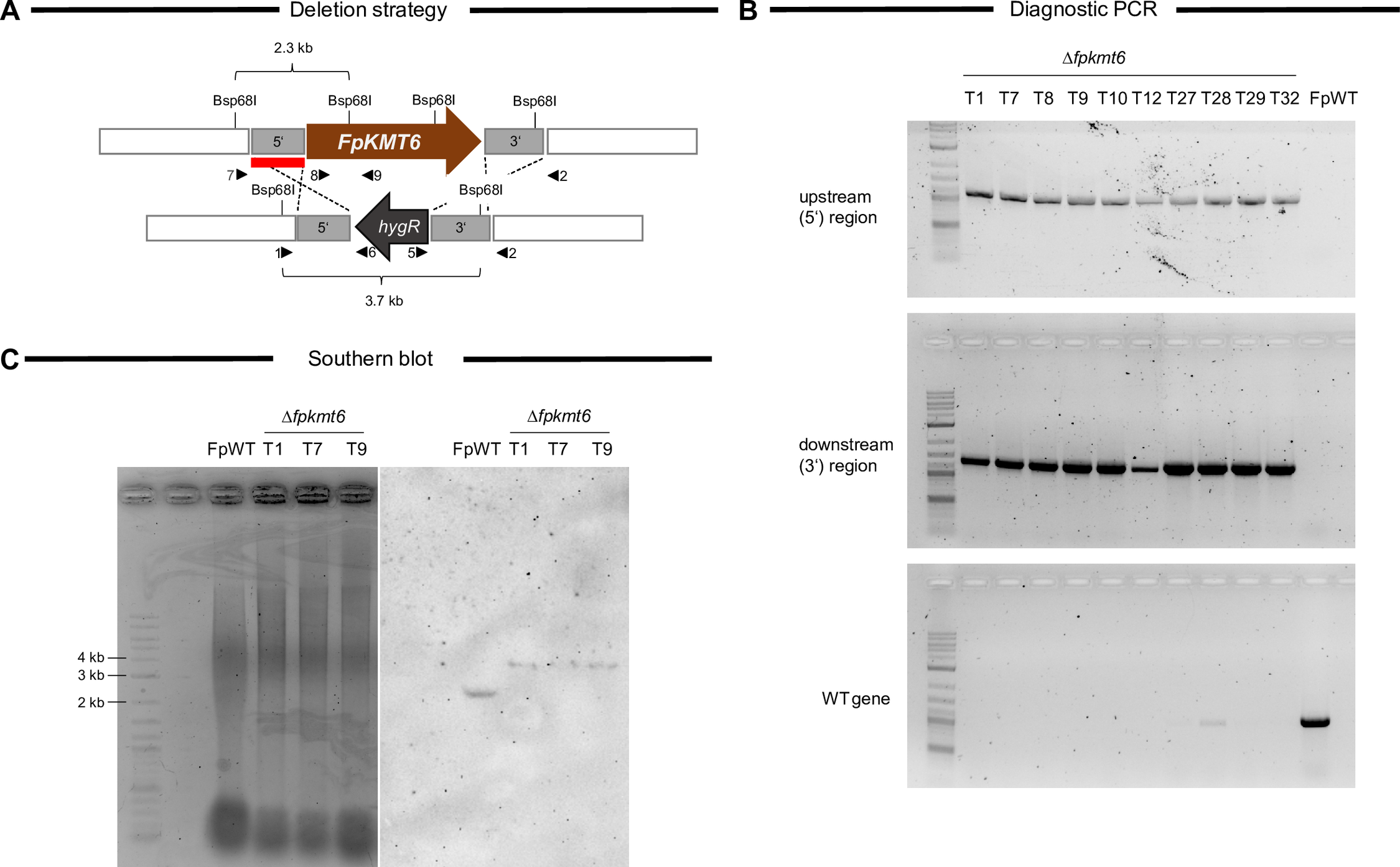

Supplement: S2 Fig — (A) FpKMT6 deletion strategy in F. proliferatum NRRL62905 (FpWT). Primers used for diagnostic PCRs are shown as arrows. Probe used for Southern blotting is indicated as red bar. (B) Verification of homologous recombination of the hygromycin resistance cassette (hygR) in the native FmKMT6 locus. Homologous integration was verified by the presence of the upstream region (5’ flank) and downstream region (3’ flank). As positive (+) control FpWT gDNA was used. The 1 kb Plus DNA ladder (NEB) was used as a size marker. (C) For the verification of a single integration event of the hygR cassette in the Δfpkmt6 mutants Southern blot was performed. The left panel shows the digested DNA before hybridization of the probe. On the right panel the visualized probes for FpWT (2.3 kb) and the FpKMT6 deletion strains (3.7kb) are shown at the correct size. (TIF) [file pgen.1011075.s002.tif]

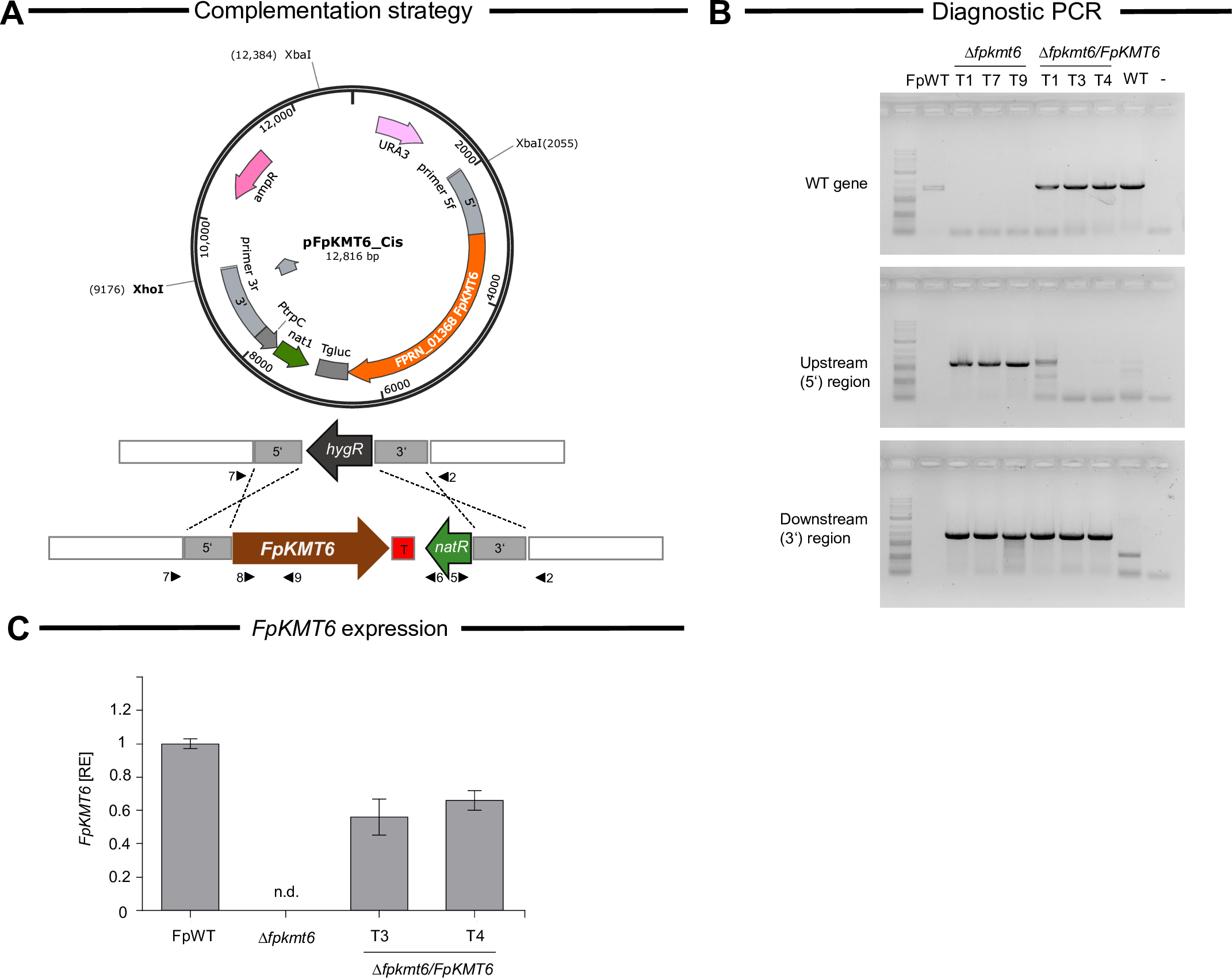

Supplement: S3 Fig — (A) FpKMT6 complementation strategy. For complementation the Δfpkmt6 T1 deletion mutant was arbitrarily chosen. XhoI and XbaI were used for plasmid linearization prior to transformation. Restriction sites are depicted in the plasmid map. Primers used for verification of successful homologous recombination are indicated in the scheme below the plasmid map. (B) Complementation was achieved by in situ integration of the native FpKMT6 wild-type gene of FpWT into Δfpkmt6 T1 using a nourseothricin resistance cassette (natR). Correct re-integration of the native FpKMT6 gene in Δfpkmt6/FpKMT6 strains was verified by diagnostic PCR. Here, presence of the upstream region (5’), downstream region (3’) and wild-type (WT) gene was tested. As negative control (5’ and 3’) gDNA of F. proliferatum NRRL62905 (FpWT) was used, while FpWT served as positive control for wild-type gene amplification. Water served as a negative control and as size marker the 1 kb Plus DNA ladder (NEB) was used. (C) Complemented Δfpkmt6/FpKMT6 strains were tested by RT-qPCR to verify reconstitution of FpKMT6 expression. FpKMT6 gene expression was restored in two independent transformants, while it is absent from the Δfpkmt6 strain. RE, relative expression. (TIF) [file pgen.1011075.s003.tif]

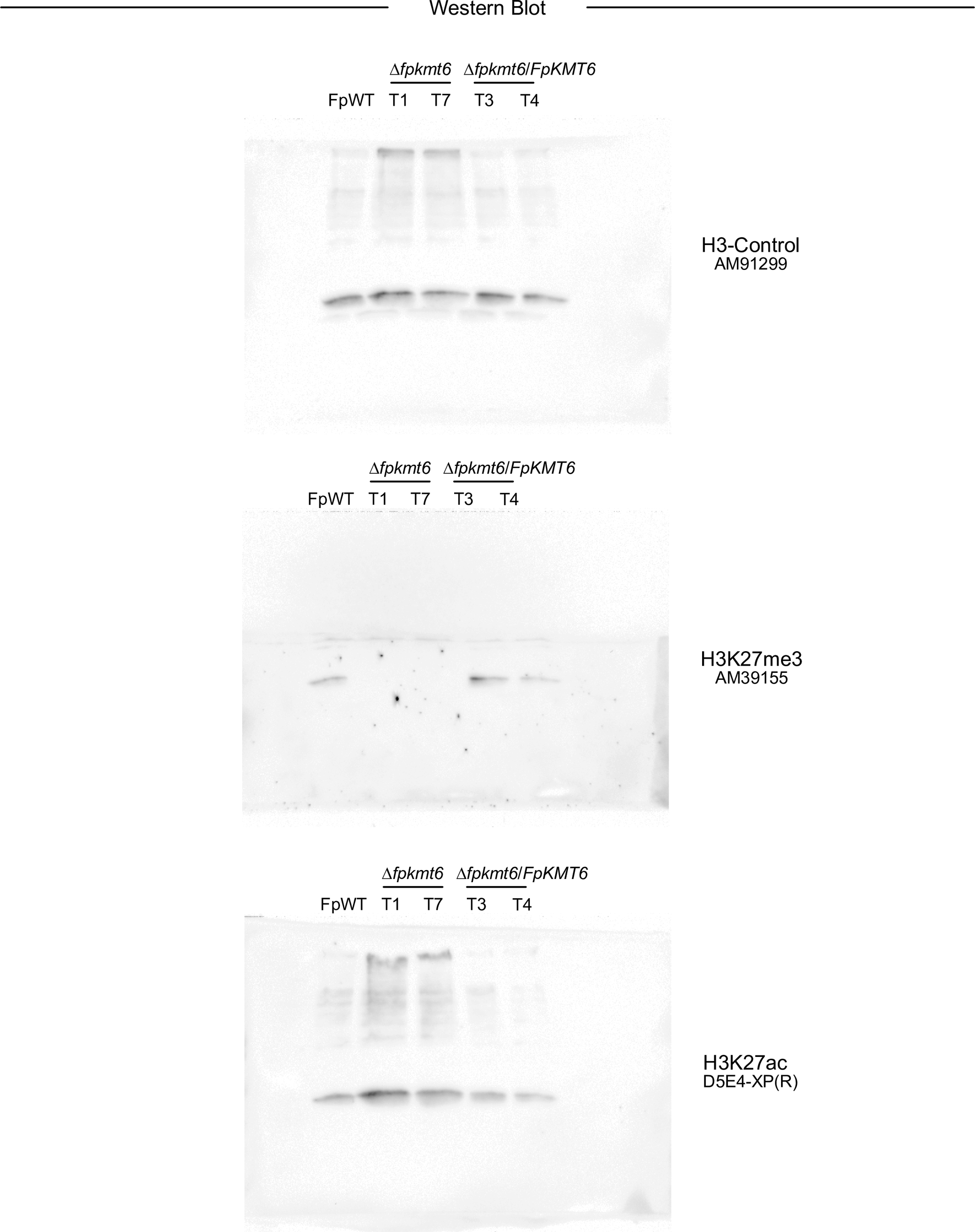

Supplement: S4 Fig — For analysis, the following antibodies were used: H3K27me3- (AM39155) and H3K27ac-specific antibody (D5E4-XP(R)) as well as a H3 control (AM91299). (TIF) [file pgen.1011075.s004.tif]

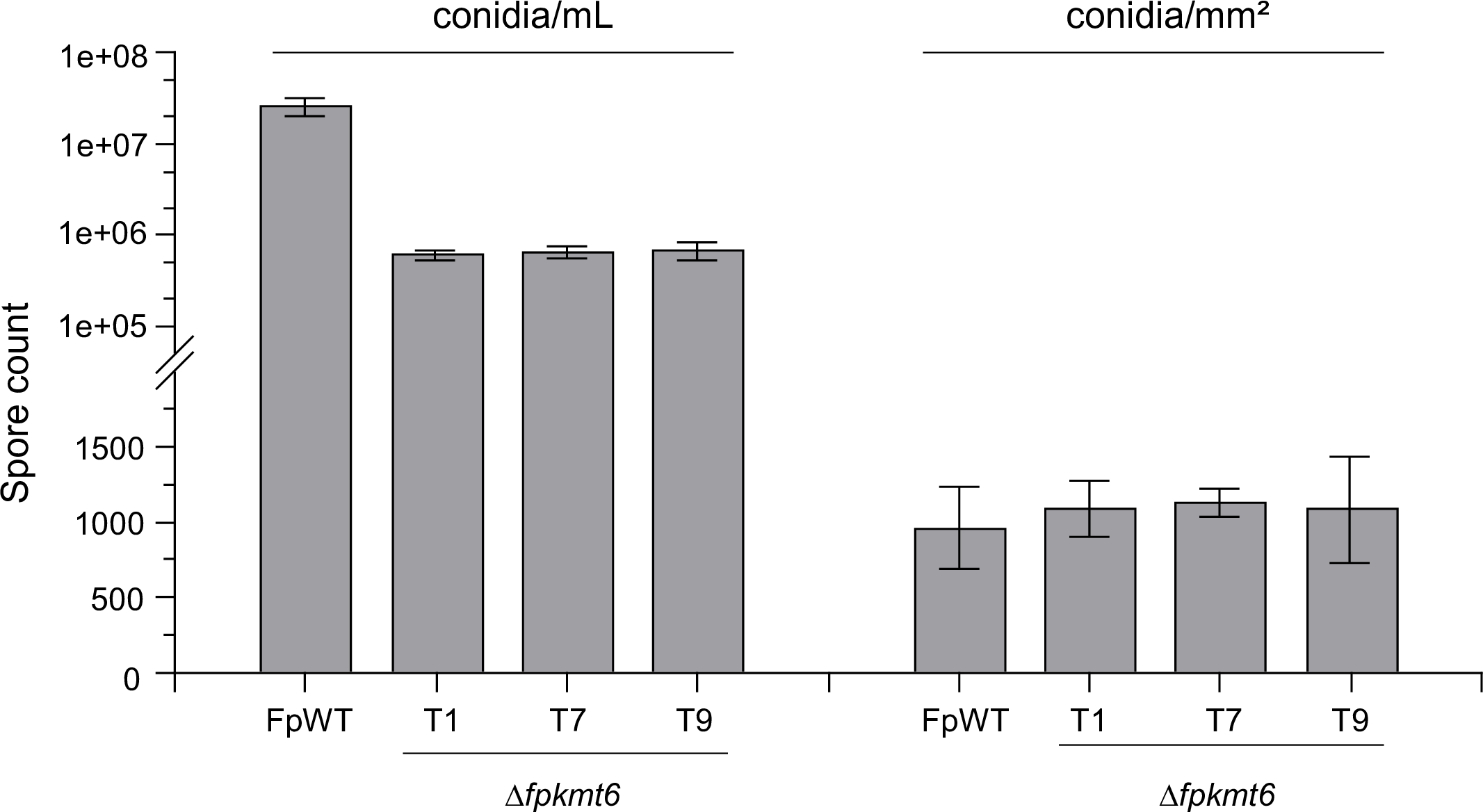

Supplement: S5 Fig — Conidia of F. proliferatum NRRL62905 (FpWT) and three independent Δfpkmt6 strains were quantified using a Neubauer improved counting chamber. Experiments were performed in biological triplicates, mean values are shown. While the overall conidia count was decreased significantly, the conidia/mm2 count was wild type-like when related to the radial hyphal growth of the respective strains. (TIF) [file pgen.1011075.s005.tif]

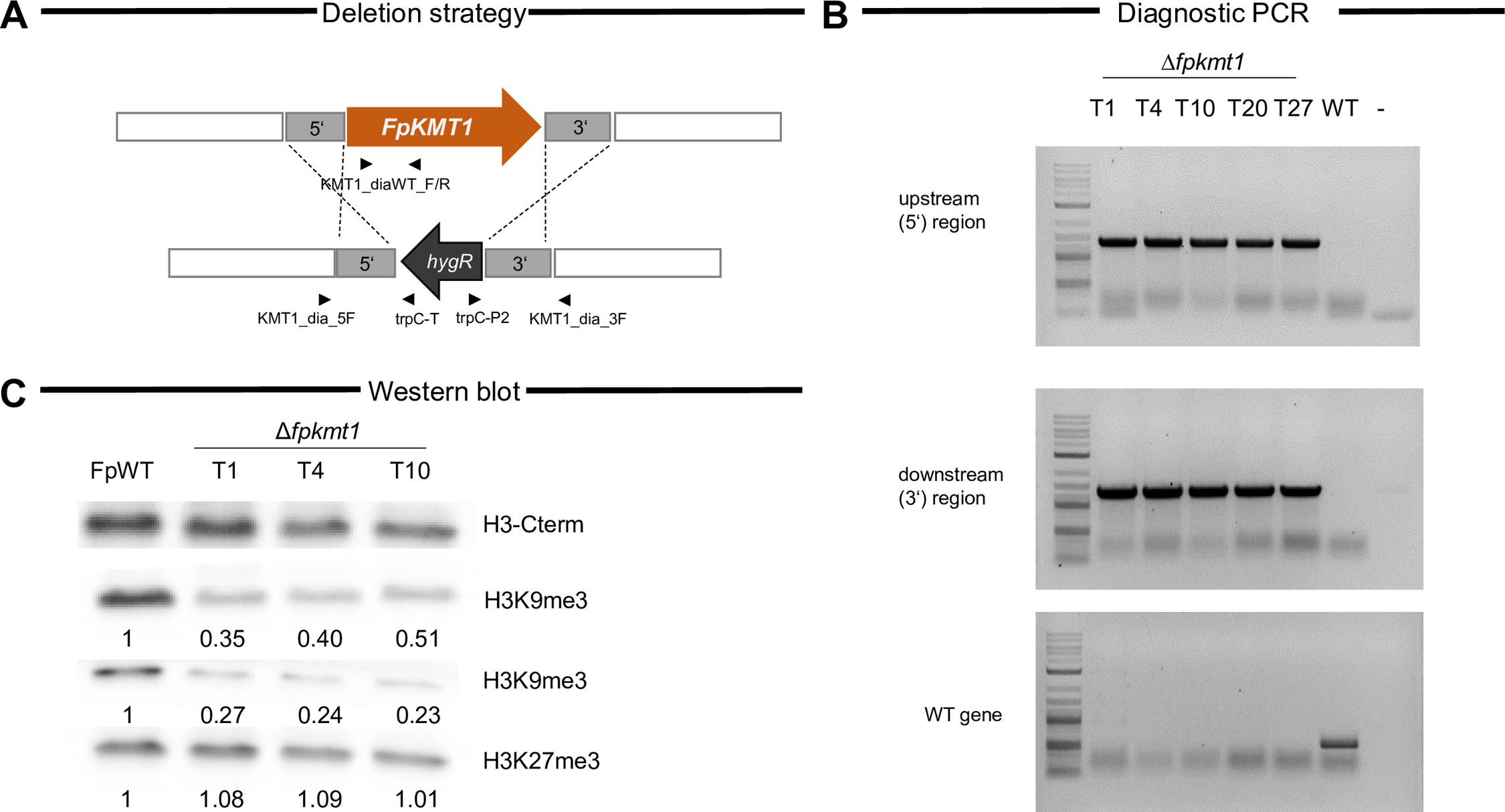

Supplement: S6 Fig — (A) Gene deletion strategy of Δfpkmt1 mutants in F. proliferatum NRRL62905 (FpWT). Primers used for diagnostic PCRs are shown as arrows in the schemata. (B) Verification of the homologous integration with the hygromycin resistance cassette (hygR) in the native FpKMT1 locus. The homologous integration was verified by the presence of the upstream region (5’), downstream region (3’ flank) as well as the absence of the native FpKMT1 gene. FpWT gDNA was used as negative (5’, 3’) as well as positive control (native wild-type gene). Sterile IonEx was used as a negative control (-). The 1 kb Plus DNA ladder (NEB) was used as a size marker. (C) Western blot analysis of FpWT and FpKMT1 deletion strains using a H3K9me3- (AM 39161/ab8898) and H3K27me3-specific antibody (AM39155) as well as a H3 control (AM91299). For quantification, a densitometric analysis was performed and the respective wild-type strain was arbitrarily set as 1. (TIF) [file pgen.1011075.s006.tif]

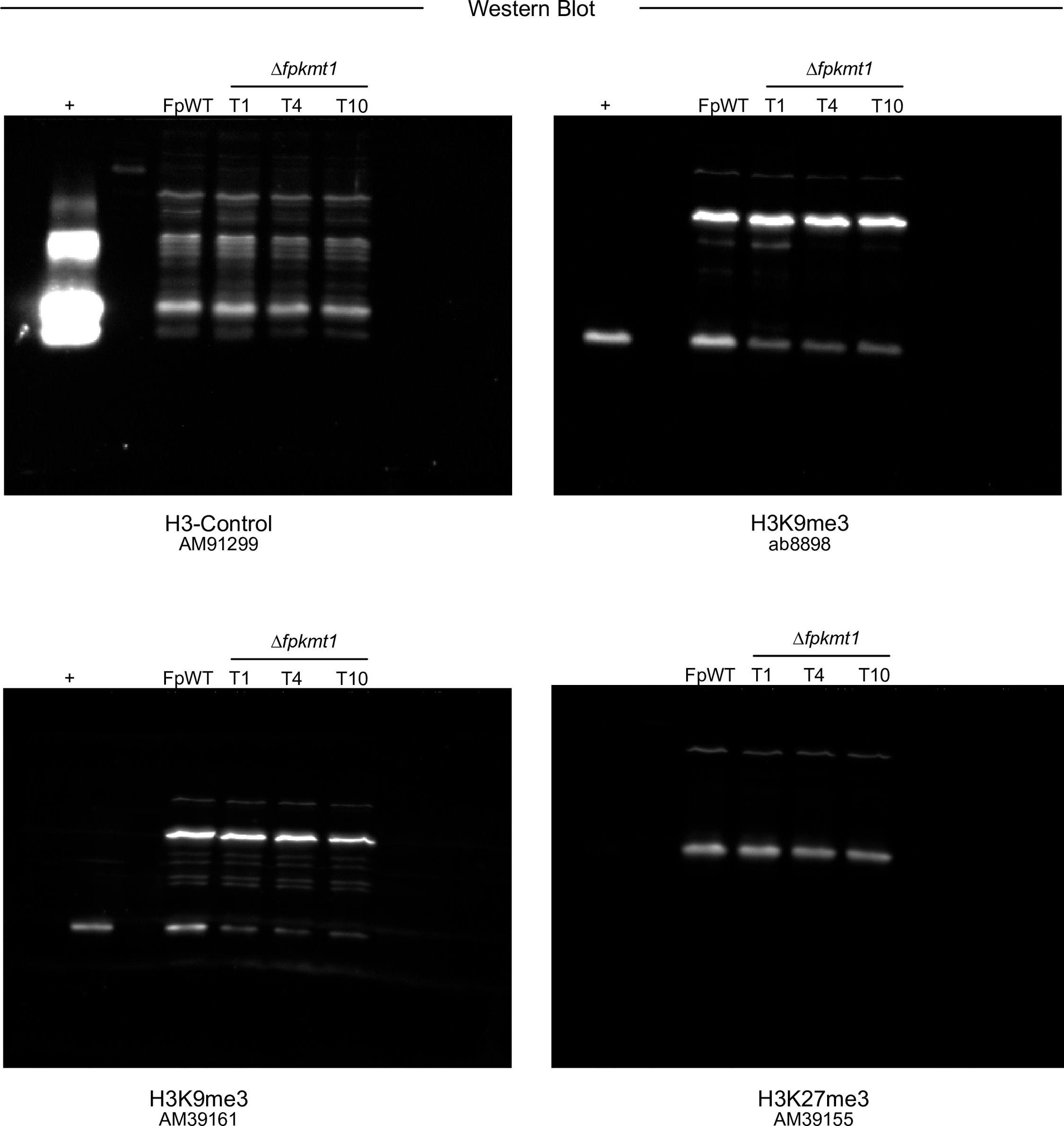

Supplement: S7 Fig — For analysis following antibodies were used: H3K9me3- (AM39161/ab8898) and H3K27me3-specific antibody (AM39155) as well as a H3 control (AM91299). (TIF) [file pgen.1011075.s007.tif]

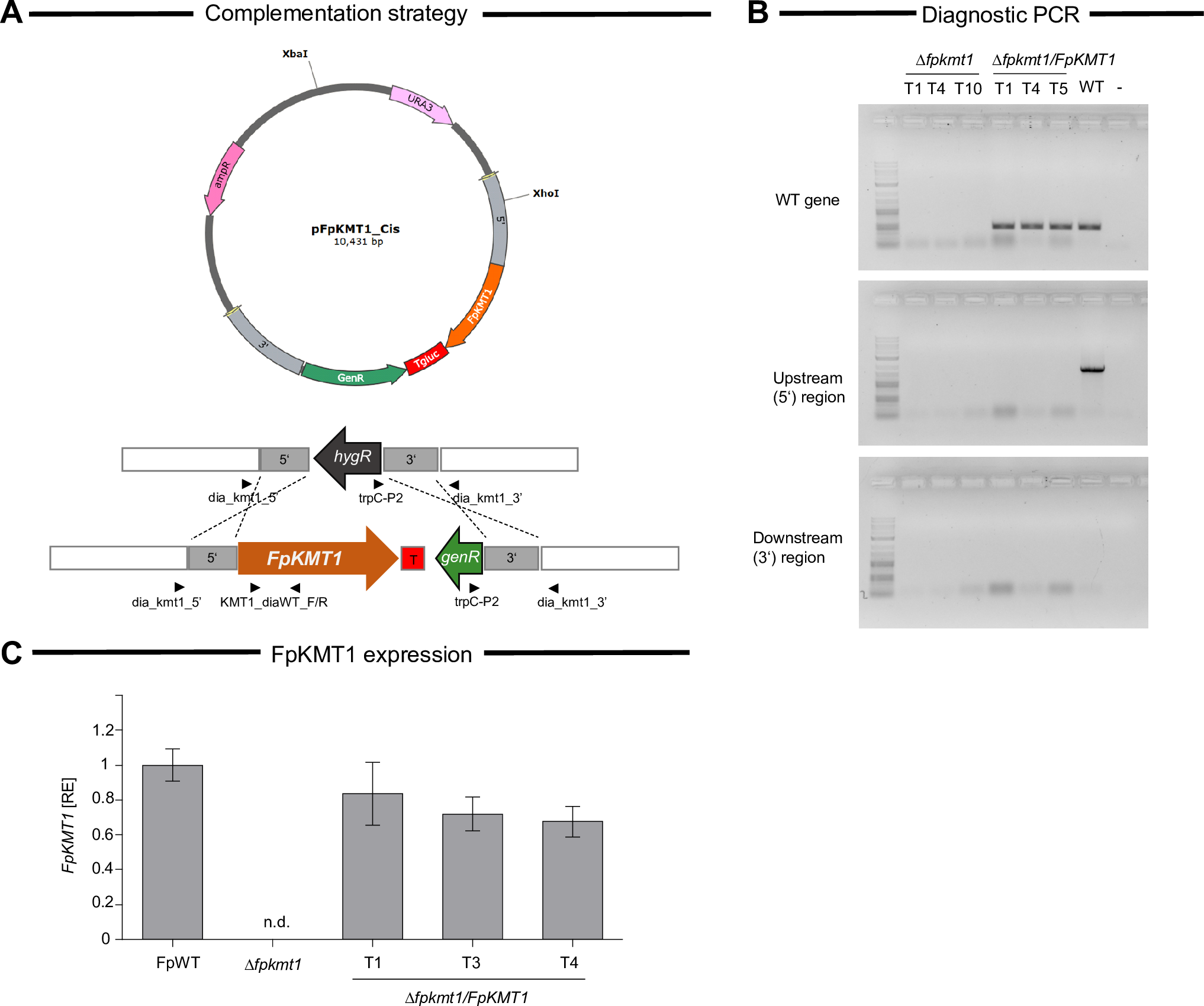

Supplement: S8 Fig — (A) FpKMT1 complementation strategy. For complementation, the Δfpkmt1 T4 deletion mutant was arbitrarily chosen. XhoI and XbaI were used for plasmid linearization prior to transformation. Restriction sites are depicted in the plasmid map. Primers used for verification of successful homologous recombination are indicated in the scheme below the plasmid map. (B) Complementation was achieved by in situ integration of the native FpKMT6 wild-type gene of FpWT into Δfpkmt6 T1 using a geneticin resistance cassette (genR). Correct re-integration of the native FpKMT1 gene in Δfpkmt1/FpKMT1 strains was verified by diagnostic PCR. Here, presence of the upstream region (5’), downstream region (3’) and wild-type (WT) gene was tested. As negative control (5’ and 3’) gDNA of F. proliferatum NRRL62905 (FpWT) was used, while FpWT served as positive control for wild-type gene amplification. Water served as a negative control. As size marker 1 kb Plus DNA ladder (NEB) was used. (C) Complemented Δfpkmt1/FpKMT1 strains were tested by RT-qPCR to verify reconstitution of FpKMT1 expression. FpKMT1 gene expression was restored in three independent transformants, while it is absent from the Δfpkmt1 strain. RE, relative expression. (TIF) [file pgen.1011075.s008.tif]

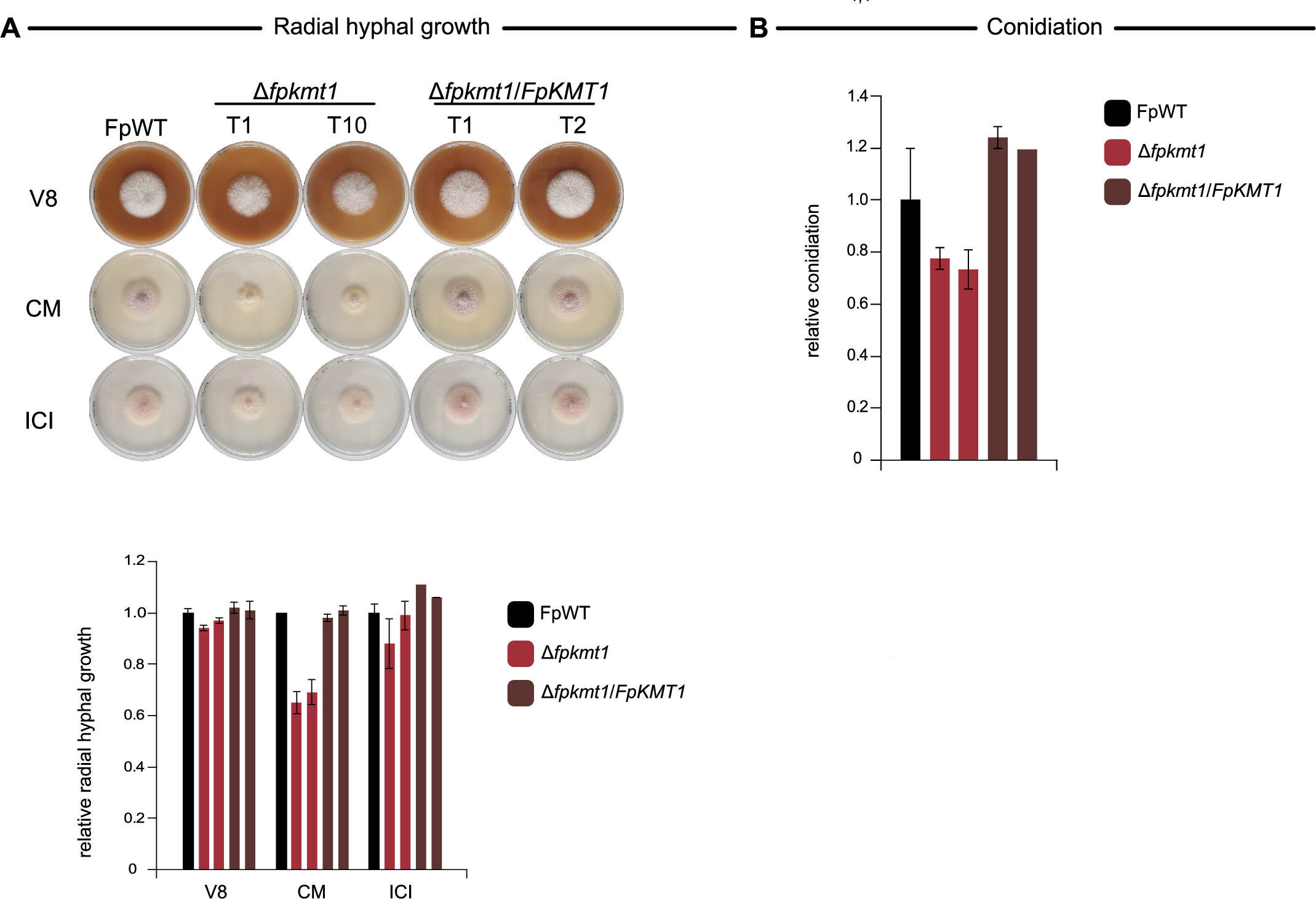

Supplement: S9 Fig — (A) Radial hyphal growth of the F. proliferatum NRRL62905 wild-type strain (FpWT) as well as the FpKMT1 deletion (Δfpkmt1) and complementation (Δfpkmt1/FpKMT1) strains on different growth media. CM, V8 (complete media) and ICI (minimal medium) were inoculated with an agar plug and incubated for 5 days post inoculation at 30°C in the dark. Experiments were performed in biological triplicates. Hyphal growth of FpWT on the respective media was arbitrarily set to 1. Mean values and standard deviations are shown in the diagram. (B) Conidiation assay using FpWT, the FpKMT1 deletion strain and ΔFpKMT1/FpKMT1 complementation strain. Conidiation was induced on V8 and samples were incubated for 7 days under a light / dark cycle (18h/6h), 20°C and 70% humidity. Experiments were performed in triplicates. Conidia production of FpWT was arbitrarily set to 1. Mean values and standard deviations are shown in the diagram. (TIF) [file pgen.1011075.s009.tif]

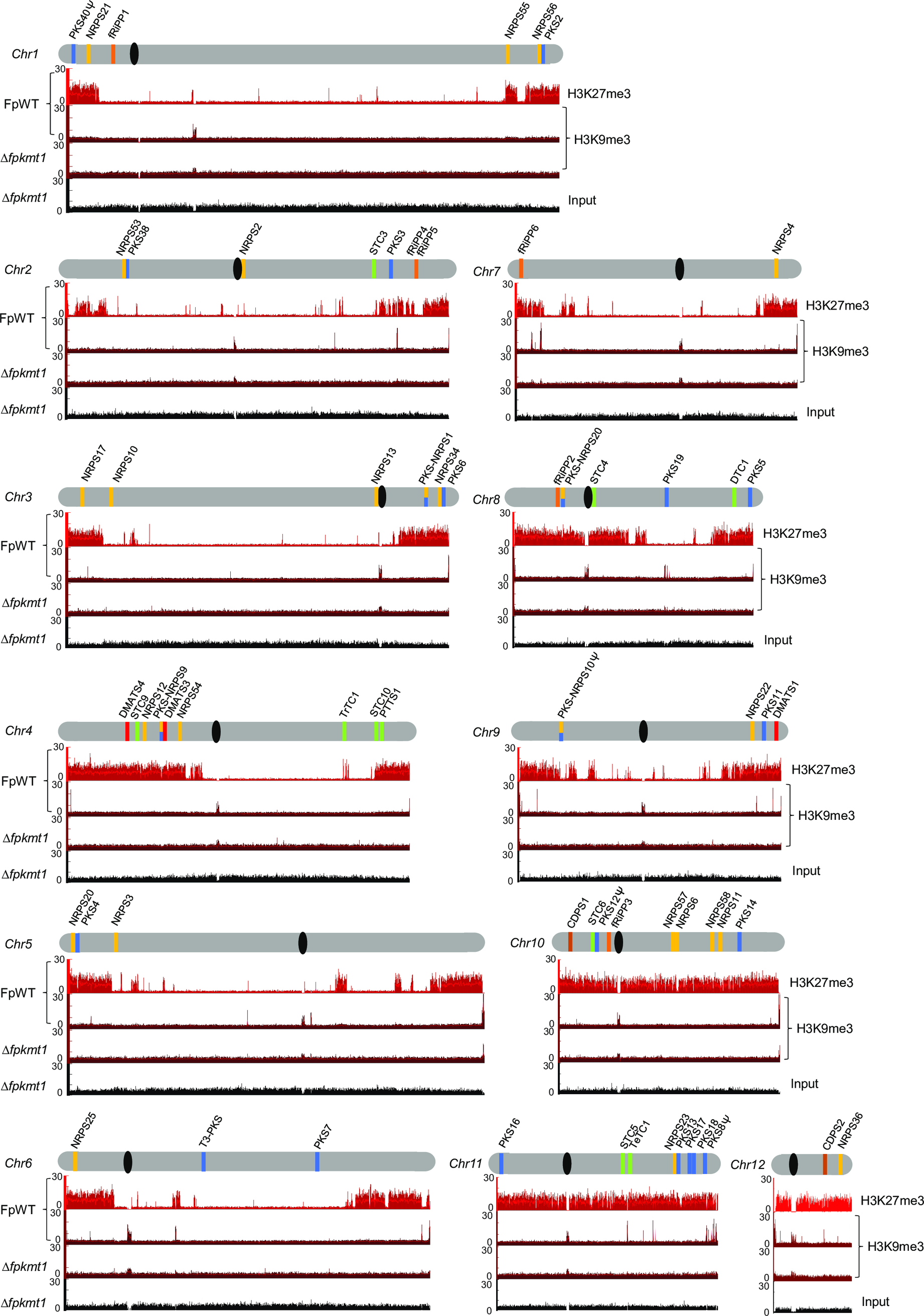

Supplement: S10 Fig — The twelve chromosomes are shown in grey i.e., Chr1 –Chr12, and centromeres are shown in black; SM key enzyme-encoding genes are indicated by bars according to the following color code: polyketide synthase (PKS), blue; non-ribosomal peptide synthetase (NRPS), orange; (sesqui-/di-/sester-/tri-/tetra-) terpene cyclase (STC/DTC/PTTS/TrTC/TeTC), light green; dimethylallyl tryptophan synthase (DMATS), red; putative fungal RIPPs, light brown; cyclodipeptide synthase (CDPS), dark brown. Pseudogenes are highlighted with an ψ; Genome-wide distribution of H3K27me3 and H3K9me3, present in the wild-type strain (FpWT) as well as H3K9me3 in the Δfpkmt1 strain are depicted beneath the chromosome arms. Input control of Δfpkmt1 verifies presence of genomic DNA. (TIF) [file pgen.1011075.s010.tif]

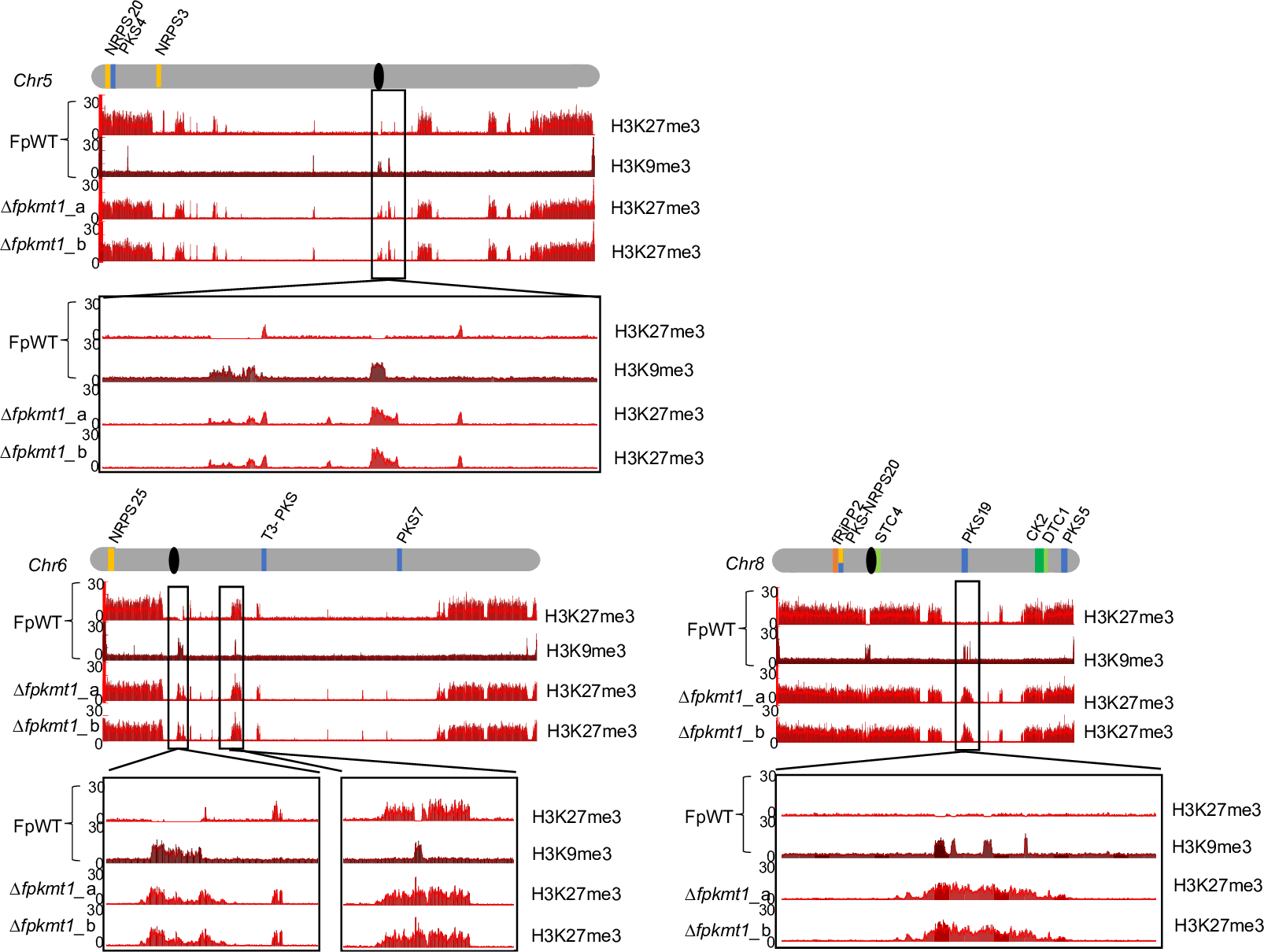

Supplement: S11 Fig — Affected chromosomes are shown in grey and centromeres are shown in black; SM key enzyme-encoding genes are indicated by bars according to the following color code: polyketide synthase (PKS), blue; non-ribosomal peptide synthetase (NRPS), orange; (sesqui-/di-/sester-/tri-/tetra-) terpene cyclase (STC/DTC/PTTS/TrTC/TeTC), light green; dimethylallyl tryptophan synthase (DMATS), red; putative fungal RIPPs, light brown; cyclodipeptide synthase (CDPS), dark brown. Pseudogenes are highlighted with an ψ; Genome-wide distribution of H3K27me3 and H3K9me3, present in the wild-type strain (FpWT) as well as H3K9me3 in the Δfpkmt6 strain in two biological replicates are depicted beneath the chromosome arms. (TIF) [file pgen.1011075.s011.tif]

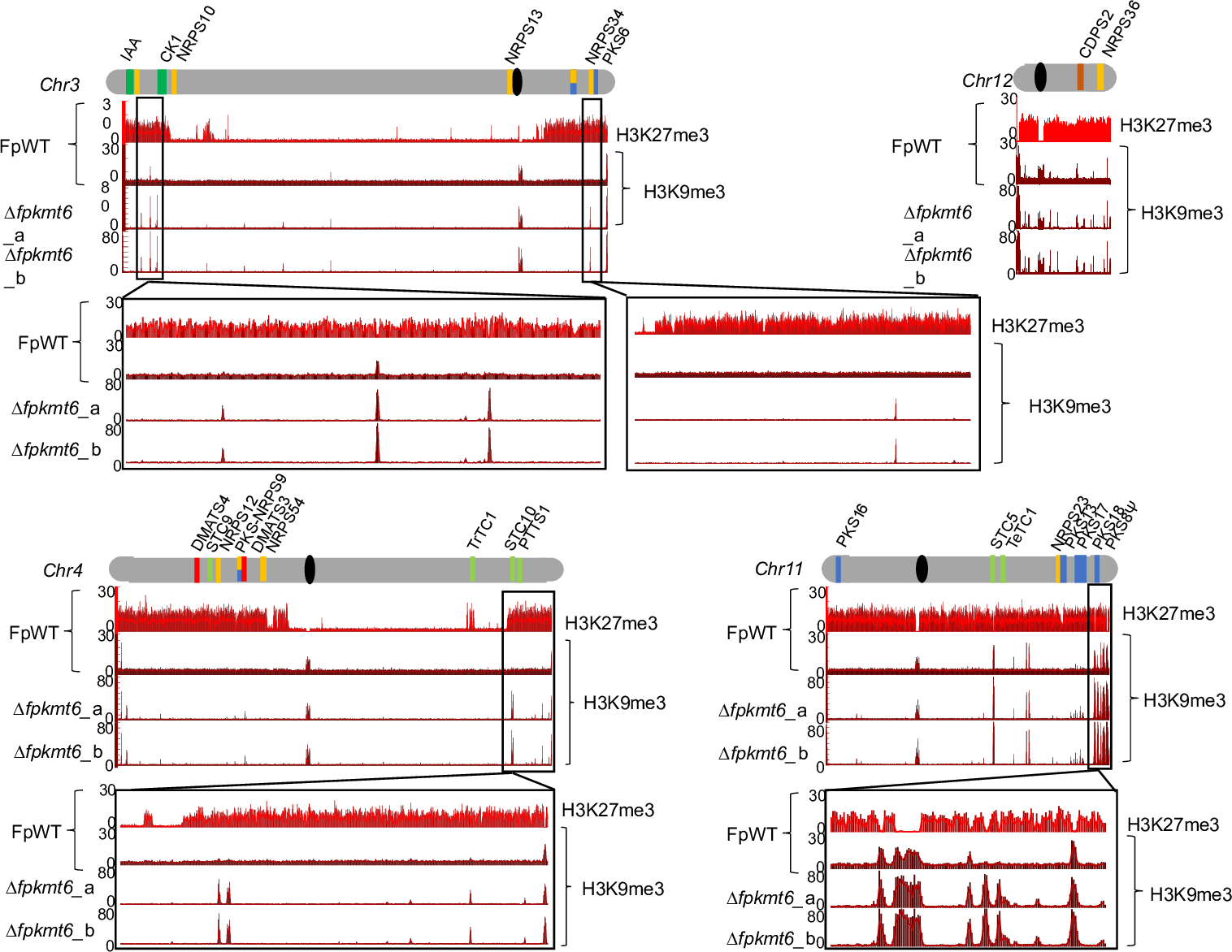

Supplement: S12 Fig — Affected chromosomes are shown in grey and centromeres are shown in black; SM key enzyme-encoding genes are indicated by bars according to the following color code: polyketide synthase (PKS), blue; non-ribosomal peptide synthetase (NRPS), orange; (sesqui-/di-/sester-/tri-/tetra-) terpene cyclase (STC/DTC/PTTS/TrTC/TeTC), light green; dimethylallyl tryptophan synthase (DMATS), red; putative fungal RIPPs, light brown; cyclodipeptide synthase (CDPS), dark brown. Genome-wide distribution of H3K27me3 and H3K9me3, present in the wild-type strain (FpWT) as well as H3K27me3 in the Δfpkmt1 strain in two biological replicates are depicted beneath the chromosome arms. (TIF) [file pgen.1011075.s012.tif]

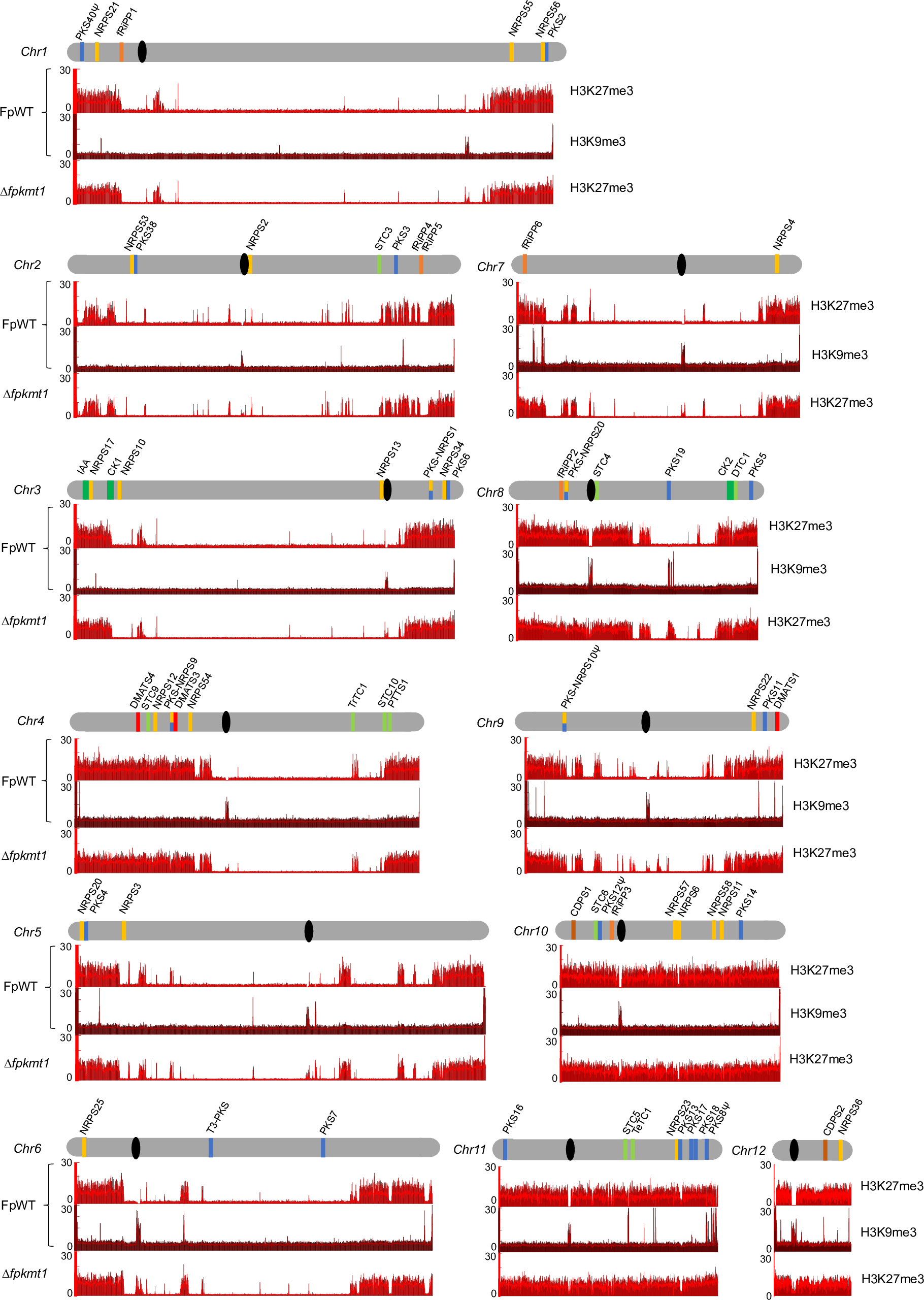

Supplement: S13 Fig — The twelve chromosomes are shown in grey i.e., Chr1 –Chr12, and centromeres are shown in black; SM key enzyme-encoding genes are indicated by bars according to the following color code: polyketide synthase (PKS), blue; non-ribosomal peptide synthetase (NRPS), orange; (sesqui-/di-/sester-/tri-/tetra-) terpene cyclase (STC/DTC/PTTS/TrTC/TeTC), light green; dimethylallyl tryptophan synthase (DMATS), red; putative fungal RIPPs, light brown; cyclodipeptide synthase (CDPS), dark brown. Pseudogenes are highlighted with an ψ; Genome-wide distribution of H3K27me3 and H3K9me3, present in the wild-type strain (FpWT) as well as H3K27me3 in the Δfpkmt1 strain are depicted beneath the chromosome arms. (TIF) [file pgen.1011075.s013.tif]

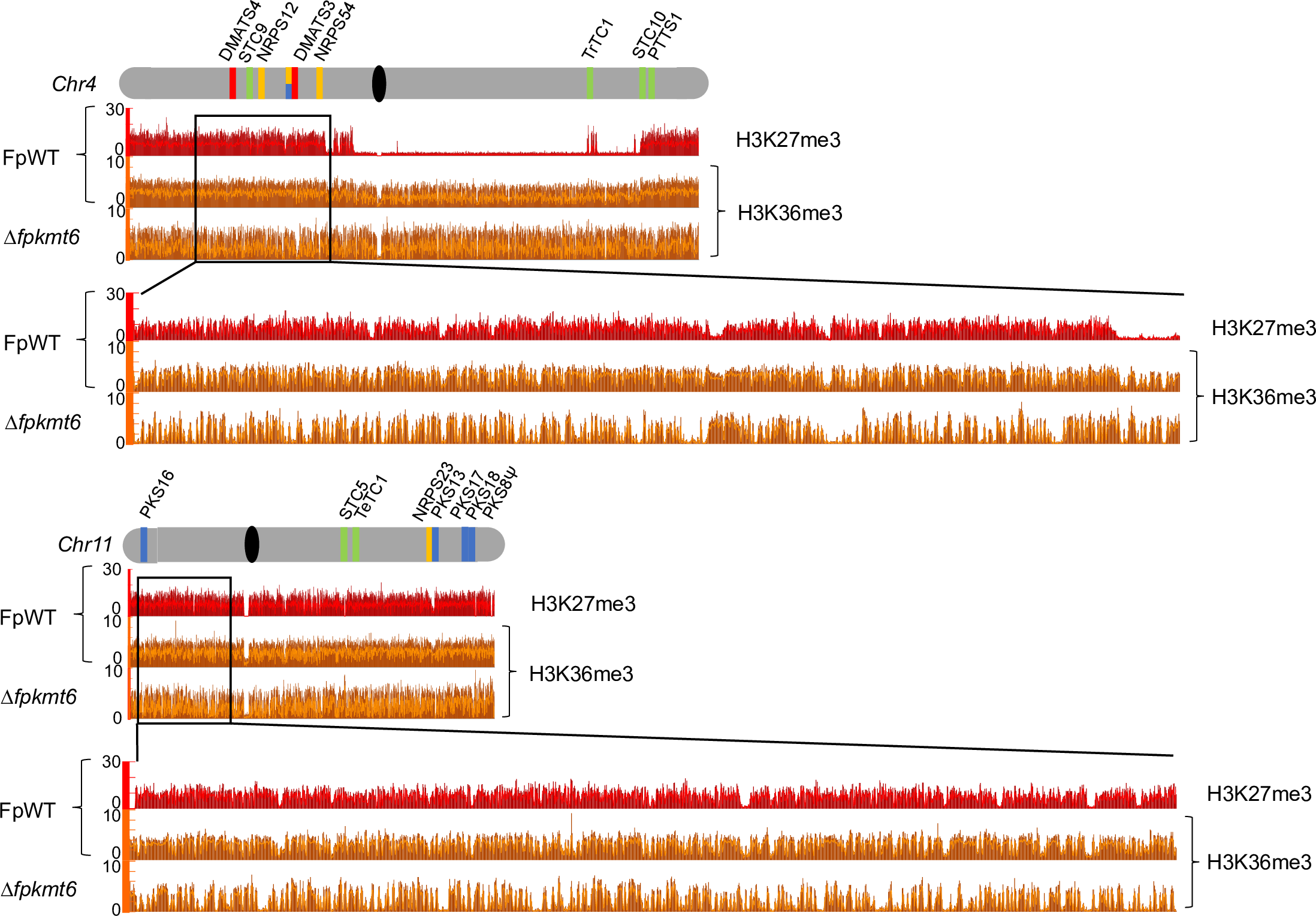

Supplement: S14 Fig — The selected chromosomes are shown in grey i.e., Chr4 and Chr11, and centromeres are shown in black; SM key enzyme-encoding genes are indicated by bars according to the following color code: polyketide synthase (PKS), blue; non-ribosomal peptide synthetase (NRPS), orange; (sesqui-/di-/sester-/tri-/tetra-) terpene cyclase (STC/DTC/PTTS/TrTC/TeTC), light green; dimethylallyl tryptophan synthase (DMATS), red. Pseudogenes are highlighted with an ψ; Genome-wide distribution of H3K27me3 and H3K36me3, present in the wild-type strain (FpWT) as well as H3K36me3 in the Δfpkmt6 strain are depicted beneath the chromosome arms. (TIF) [file pgen.1011075.s014.tif]

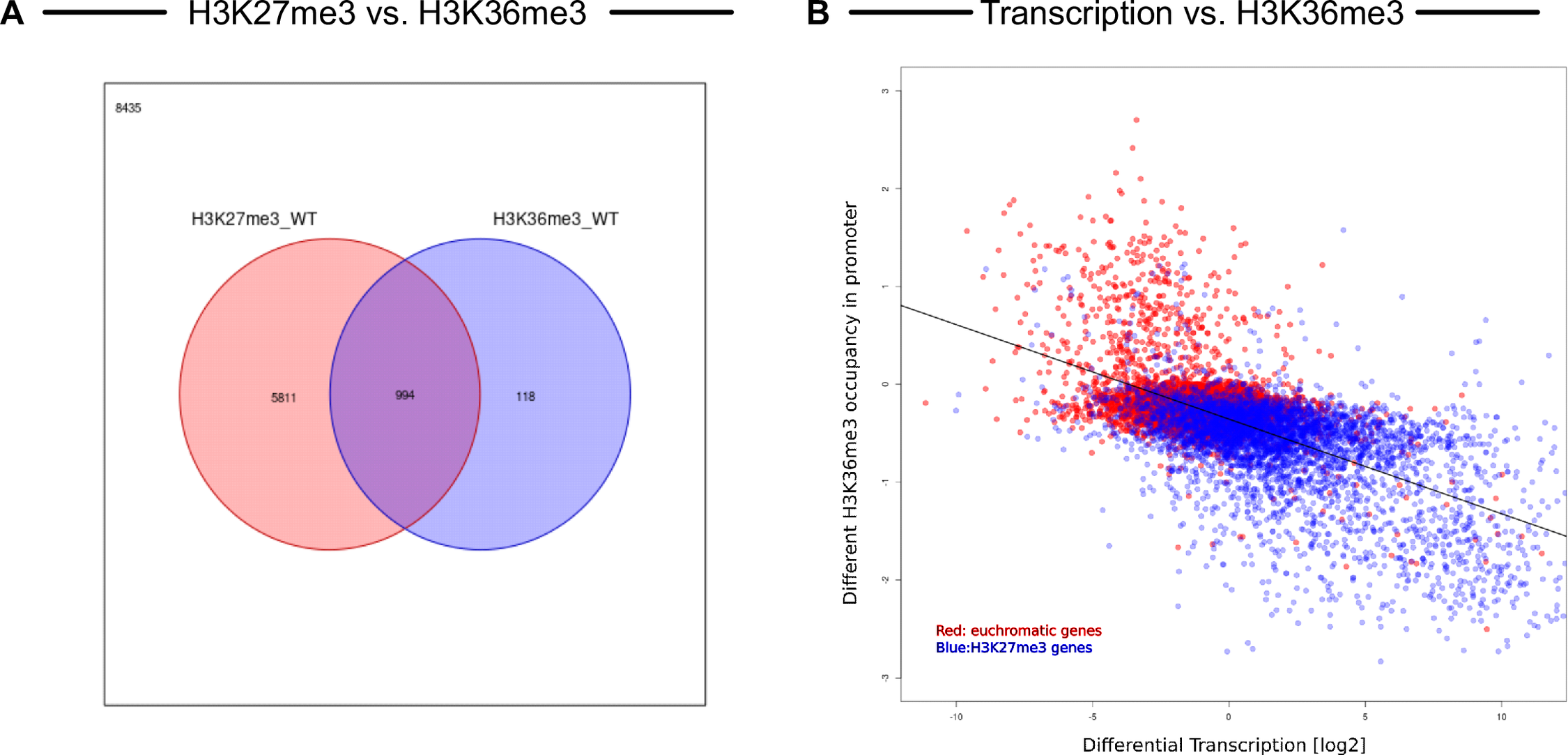

Supplement: S15 Fig — (A) Venn diagram of number of genes with H3K27me3 labeling (red) and with H3K36me3 in their promoter region (blue). Genes allocated with both histone marks are written in the intersection. (B) Different H3K36me3 occupancy in promoter regions between Δfpkmt6 and FpWT (positive numbers represent higher H3K36me3 occupancy in Δfpkmt6 (and vice versa) in respect to differential transcription between Δfpkmt6 and FpWT. Red and blue dots represent euchromatic and H3K27me3 labeled genes, respectively. (TIF) [file pgen.1011075.s015.tif]

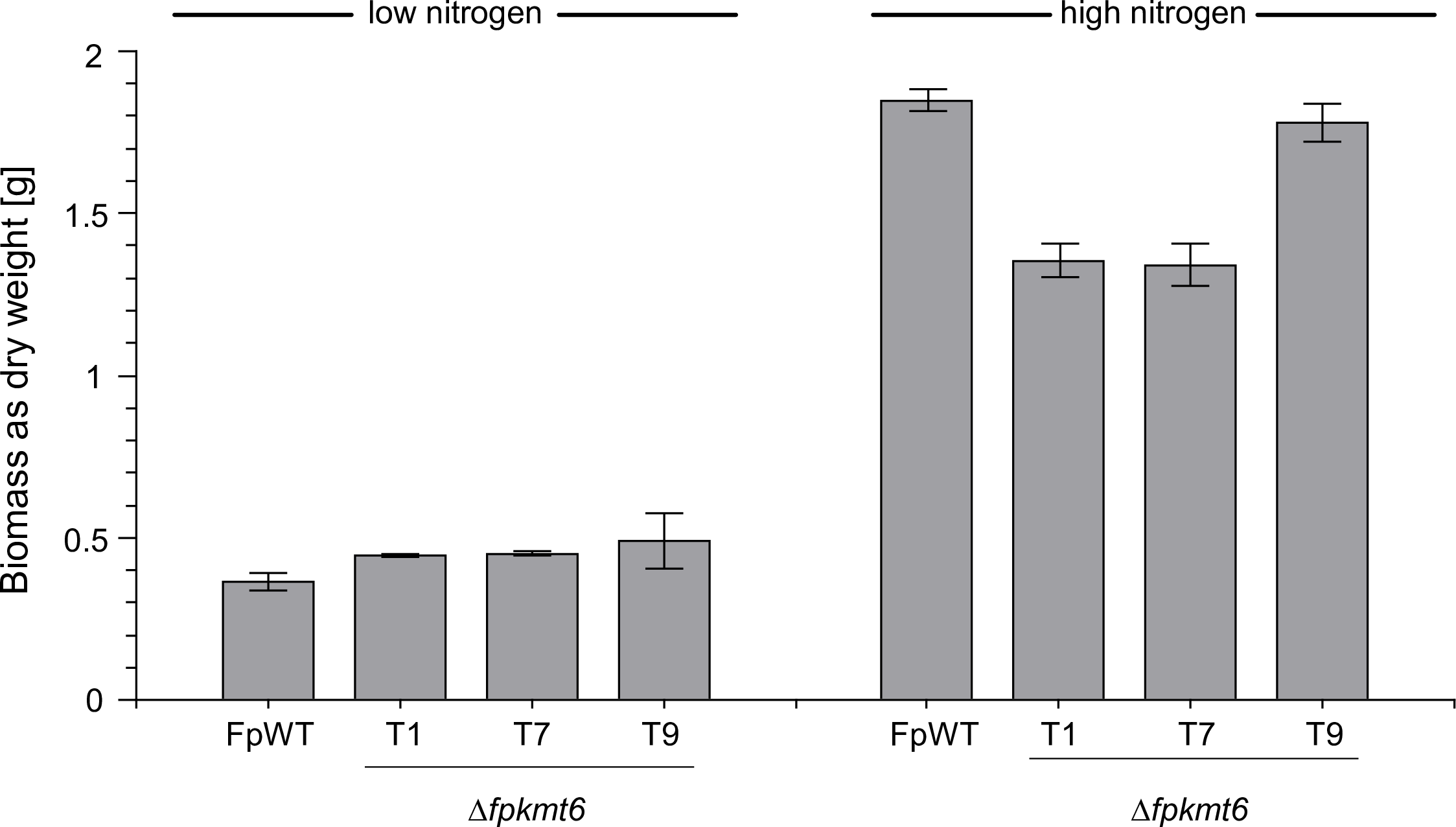

Supplement: S16 Fig — Fungal strains were cultivated in liquid ICI supplemented either with low amounts of nitrogen (6 mM glutamine) or high amounts of nitrogen (60 mM glutamine) for 7 days. The dry weight of freeze-dried fungal mycelium was quantified, and respective strains were grown in triplicates. (TIF) [file pgen.1011075.s016.tif]

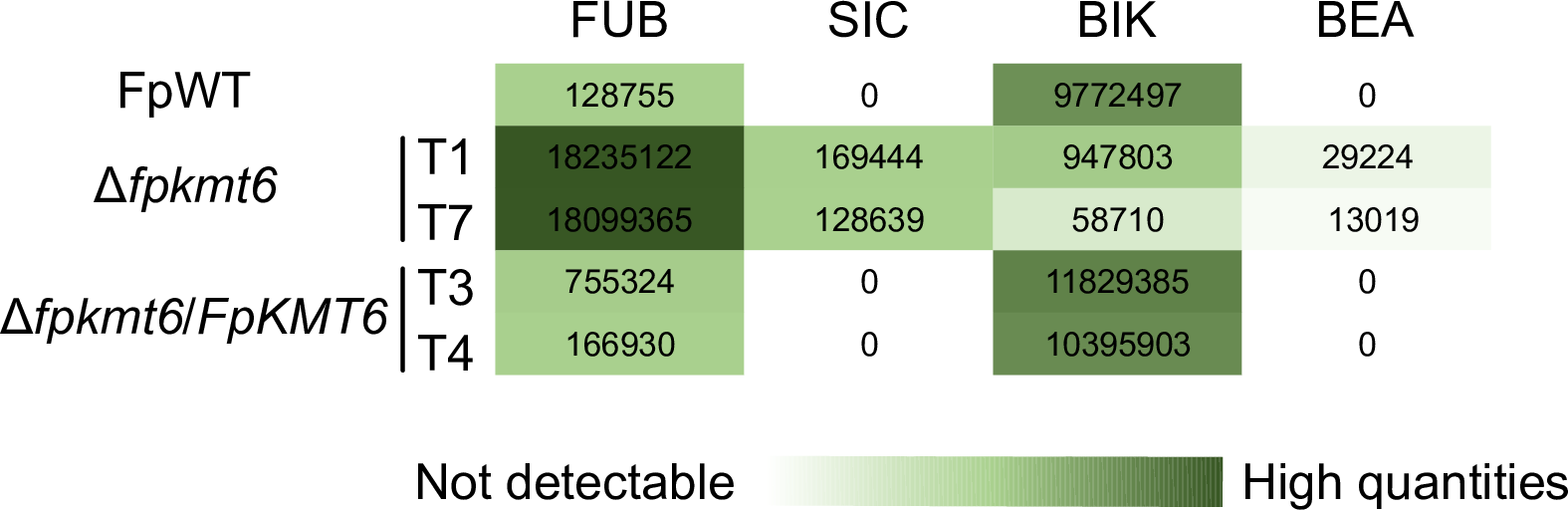

Supplement: S17 Fig — The Fusarium proliferatum NRRL62905 wild-type strain (FpWT), the FpKMT6 deletion (Δfpkmt6) and complemented (Δfpkmt6/FpKMT6) complemented strains were grown in exactly the same conditions as for RNA-seq (and ChIP-seq) i.e., in liquid ICI low nitrogen conditions (6 mM glutamine) for 7 days at 30°C. Fungal supernatants were quantified using LC-MS/MS. Quantities of known SMs are illustrated as heatmaps. Determined quantities are normalized to the biomass formation (Area/g dry weight). 0 denotes for not detected. Experiments were performed in biological triplicates and technical duplicates. (TIF) [file pgen.1011075.s017.tif]

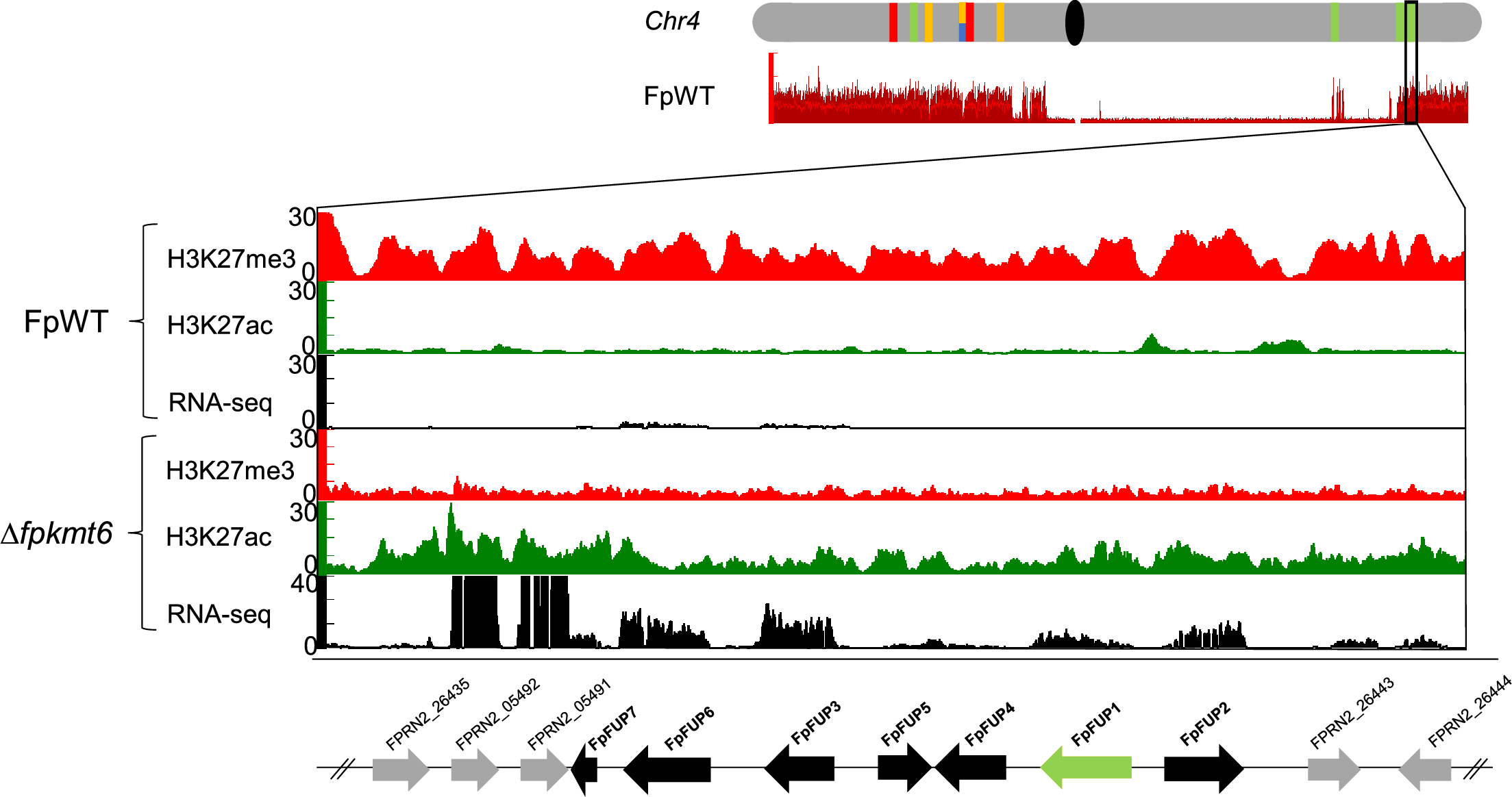

Supplement: S18 Fig — Chromosome 4 is shown in grey and centromeres are shown in black; SM key enzyme-encoding genes are indicated by bars according to the following color code: polyketide synthase (PKS), blue; non-ribosomal peptide synthetase (NRPS), orange; (sesqui-/di-/sester-/tri-/tetra-) terpene cyclase (STC/DTC/PTTS/TrTC/TeTC), light green. Below the chromosome the H3K27me3 coverage in the F. proliferatum wild-type strain NRRL62905 (FpWT) is shown. Localization of the fusaproliferin BGC (FUP) is boxed, and relevant chromatin marks (H3K27me3 and H3K27ac) allocated with this region in FpWT and in a strain deleted for FpKMT6 (Δfpkmt6) are zoomed in. In descending order are H3K27me3 (red), H3K27ac (green), Input (grey) and transcription as determined by RNA-seq (black) for FpWT and Δfpkmt6. Below this, the selected BGC is indicated as follows: the key enzyme-encoding gene is depicted in yellow; cluster genes are shown in black. Loss of H3K27me3 results in H3K27ac accompanied by the transcription of the otherwise silent FUP BGC. (TIF) [file pgen.1011075.s018.tif]

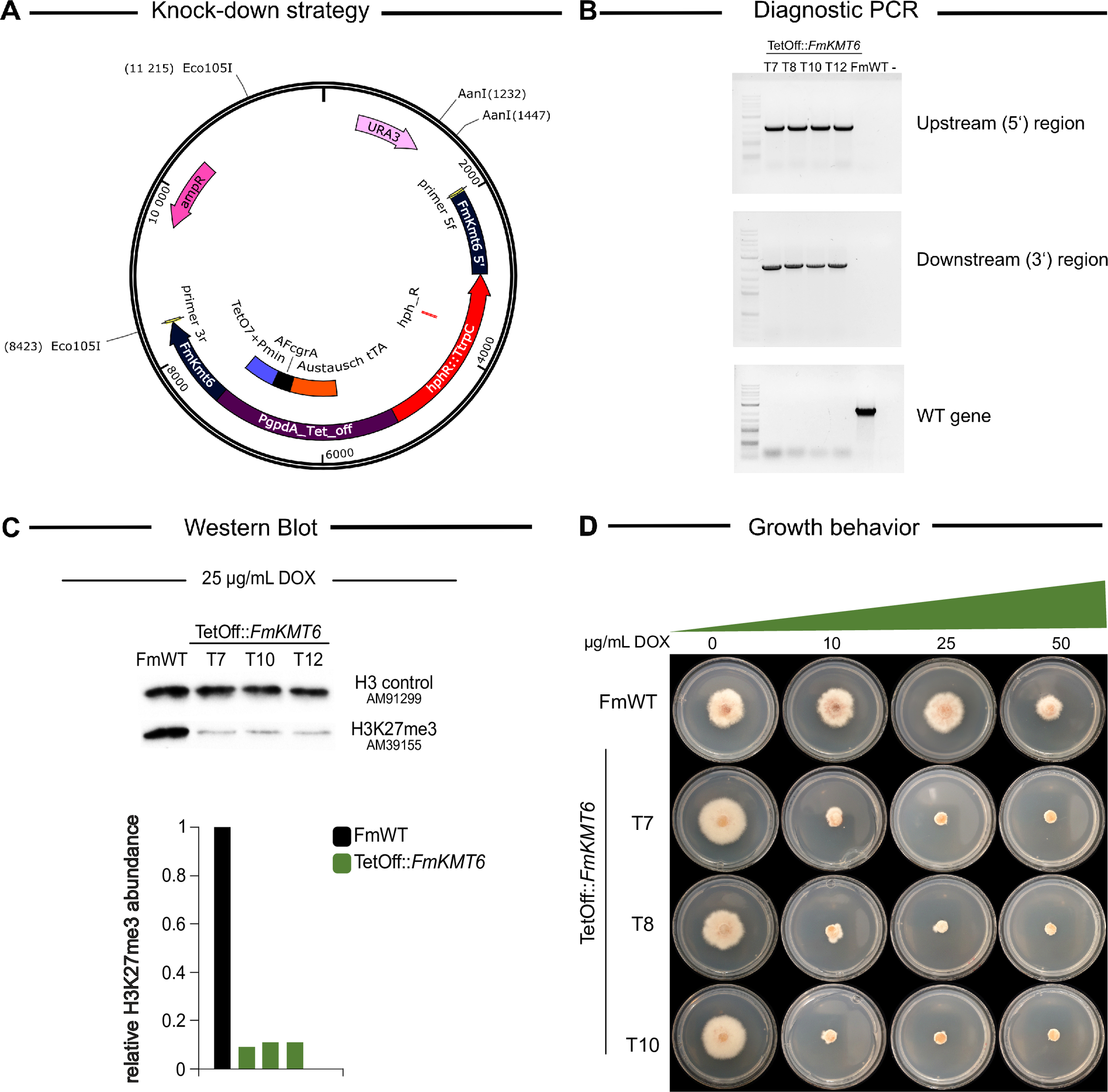

Supplement: S19 Fig — (A) Plasmid map of the TetOff::FmKMT6 construct. Before fungal transformation the plasmid was linearized with the restriction enzymes Eco105 and AanI. (B) Verification of the in-locus integration of the knock-down construct and absence of the native wild-type gene. The upstream (5’) and downstream (3’) regions were amplified using the primer pairs dia_KMT6_5’//trpC-T and dia_KMT6_3’//trpC-P2, respectively. Absence of the wild-type gene was verified with the primers kmt6_WT_diaF2 and kmt6_WT_diaR2. FmWT genomic DNA was used as a negative (5’/3’) and positive control (wild-type gene). As negative control (-) sterile IonEx was used. (C) Verification of FmKMT6 knock-down via western blot analysis from FmWT and TetOff::FmKMT6 strains using an H3K27me3-specific antibody (AM39155) and a H3 control (AM91299). For quantification, a densitometric analysis was performed and the respective wild-type strain was arbitrarily set as 1. (D) Radial hyphal growth assay using FmWT and the TetOff::FmKMT6 strains on ICI (6 mM glutamine) supplemented with 0–50 μg/mL DOX. Plates were inoculated with an agar plug and incubated for 4 days at 30°C in the dark. Experiments were performed in biological triplicates. (TIF) [file pgen.1011075.s019.tif]

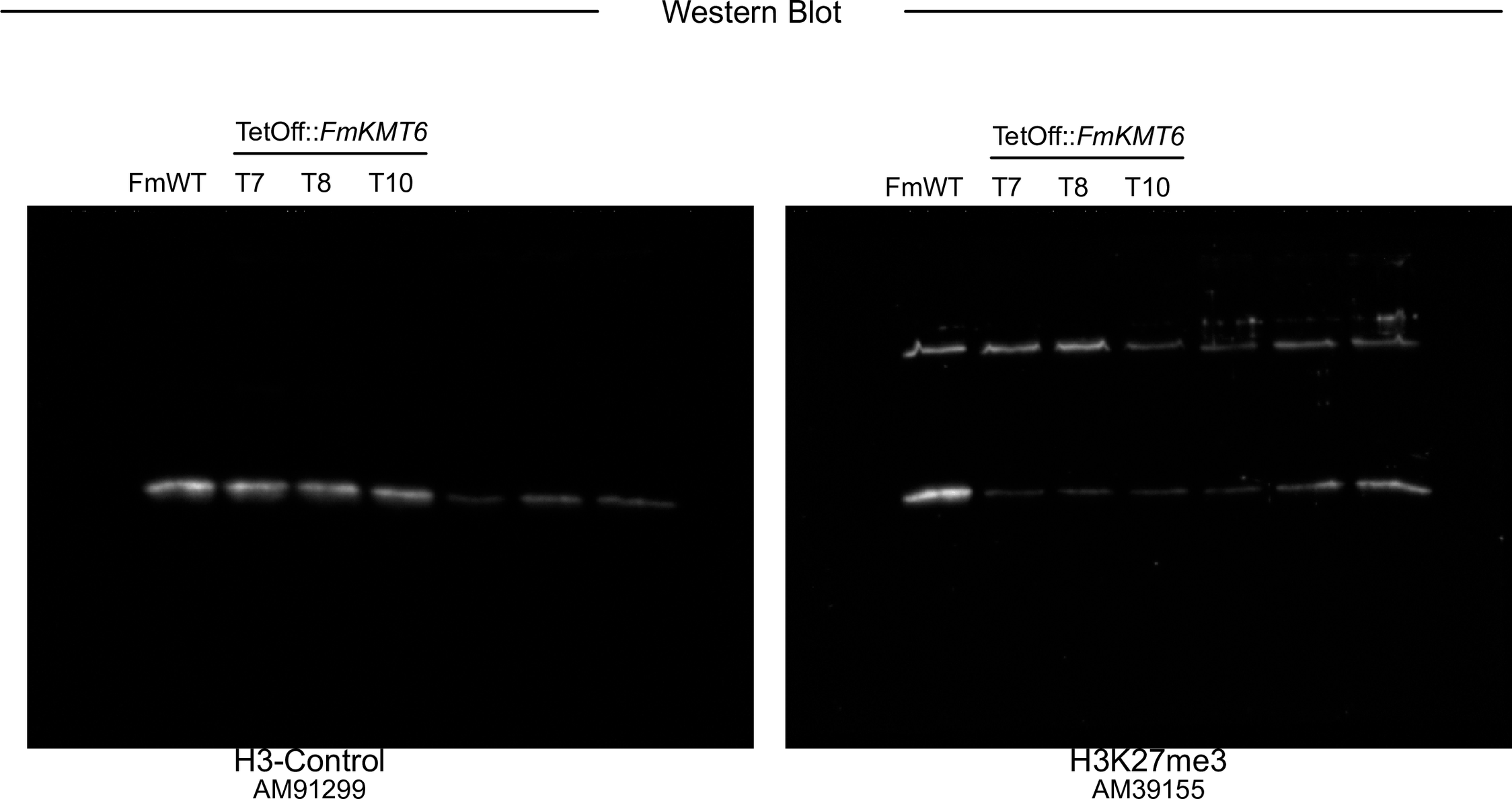

Supplement: S20 Fig — For analysis, the following antibodies were used: H3K27me3-specific antibody (AM39155) and a H3 control (AM91299). (TIF) [file pgen.1011075.s020.tif]
