# Supplementary material for: H3K27me3 is vital for fungal development and secondary metabolite gene silencing, and substitutes for the loss of H3K9me3 in the plant pathogen Fusarium proliferatum
Source: PLoS Genet. 2024 Jan 2;20(1):e1011075. doi: 10.1371/journal.pgen.1011075 (PMC10786395; doi:10.1371/journal.pgen.1011075)
Supplement: S3 Table — Introduced overhangs required for yeast recombinational cloning are written in small letters. (DOCX) [file pgen.1011075.s023.docx]

**S3 Table. Primers used during this study.** Introduced overhangs required for yeast recombinational cloning are written in small letters.

| Primer identifier | Primer sequence |
| --- | --- |
| **Generation and verification of deletion and complementation strains** | |
| KMT6_5F | gtaacgccagggttttcccagtcacgacgGATCGCAGCTTATAAGCATCCG |
| KMT6_5R | atccacttaacgttactgaaatctccaacCTTTGCCTCAAACTCTAAGGC |
| KMT6_3F | ctccttcaatatcatcttctgtctccgacCTTCCAAGGTGGTTTGGCTTC |
| KMT6_3R | gcggataacaatttcacacaggaaacagcTGCCCACCTGCTATGTCGTGG |
| hph_R | GTTGGAGATTTCAGTAACGTTAAGTGGAT |
| hph_F | GTCGGAGACAGAAGATGATATTGAAGGAGC |
| Dia_KMT6_WT_F (**8**) | AGACAGTGATTCAGACTACG |
| Dia_KMT6_WT_R (**9**) | TACAGCCAGGTATTGAGAGC |
| dia_KMT6_5’ (**7**) | TTGCCTTACTCATTACGTCG |
| dia_KMT6_3’ (**2**) | AAAGGTACATGTTGCGCTGG |
| trpC-P2 (**5**) | GTGATCCGCCTGGACGACTAAACC |
| trpC-T (**6**) | GGAATAGAGTAGATGCCGACCGG |
| Fm_diakmt6_5F (**1**) | atgctgttaaactaccggagcc |
| kmt6_WT_diaF2 (**3**) | ctcatcatctcgtcgccatcg |
| kmt6_WT_diaR2 (**4**) | gtgatcgcatcgtcaggctct |
| KMT6_R1 | CTCTACTGGAGACATGGACACATTCAGC |
| KMT6_F1 | CTGAATGTGTCCATGTCTCC |
| FpKMT6_Cil_Tgluc_R2 | CATACATCTTATCTACATACGttattgactcccgcctaaccagtc |
| Tgluc_F2 | CGTATGTAGATAAGATGTATG |
| KMT1_5F | aacgccagggttttcccagtcacgacgTGATCCAGTTGAATCTCGGTCG |
| KMT1_5R | ccacttaacgttactgaaatctccaacCAGTGTTTACTAGAGAGGATGG |
| KMT1_3F | ccttcaatatcatcttctgtctccgacAGGGAGGAAGGTATCTGGACGC |
| KMT1_3R | ggataacaatttcacacaggaaacagcGCAAAGAGCTTCATGACCATGG |
| dia_kmt1_5’ | TGAATGGTTATAGACGGGAGCC |
| dia_kmt1_3’ | GAGGATGTATTCGTGAATGG |
| KMT1_Cil-Tgluc_R | CATACATCTTATCTACATACGtcaccacaagaacttcctgcac |
| KMT1_dia_WT_F | AACGCCATTTCTACTTCCACGG |
| KMT1_dia_WT_R | TGGGCAGTCTATACTACAGG |
| genR_R | CTGATAGCGGTCTGCCACAC |
| TtrpC_R | CCTCTAAACAAGTGTACCTG |
| Tet-off_R | GGTGTTTAAACGGTGATGTC |
| TetOff_KMT6_5R | ACAGGTACACTTGTTTAGAGGctttgcctcaaactctaaggc |
| TetOff_KMT6_3F | GACATCACCGTTTAAACACCatggcctctcatcatctcgt |
| **Primers used for RT-qPCR** | |
| cDNA_check_Actin_F | GTATGTGCAAGGCCGGTTTCG |
| cDNA_check_Actin_R | GAGACCAGGGTACATGGTGG |
| Actin_F | CCACCATGTACCCTGGTCTCTCC |
| Actin_R | AATGGAACCACCGATCCAGACGG |
| ß-TUB_F | GAGGCAGTACGATGGCATGCG |
| ß-TUB_R | GGTAATCTGCGTCTTCAGCAGCTTCG |
| GPD_F | GCCTCTGAGGGTGACCTCAAGG |
| GPD_R | CGTTGTCGTACCAGGAGACCAGC |
| FpKMT1_RTqPCR_F | CCATTCTTGCGACCCCAACATGC |
| FpKMT1_RTqPCR_R | CCTCTCCCTCGTGTGAAACCCC |
| aba1_qRT_F | AGAGCCCAACGACCACATCAGG |
| aba1_qRT_R | TCCTCGTCTTCGCTTCATTGGC |
| flb3_qRT_F | CATGATGGGCCAGTTCAGCTCC |
| flb3_qRT_R | TCCTCAGAGCCAGCCTCAGAGC |
| flb4_qRT_F | TACCACCTCTGCGTCACTCAGGC |
| flb4_qRT_R | AGATGCATCCTGGAATCTTGGGC |
| wet1_qRT_F | TCTCCTAATGACAGGTGTCGCGC |
| wet1_qRT_R | ATGCAAAGCCTTGTTCCATCAGC |
