# Supplementary material for: H3K27me3 is vital for fungal development and secondary metabolite gene silencing, and substitutes for the loss of H3K9me3 in the plant pathogen Fusarium proliferatum
Source: PLoS Genet. 2024 Jan 2;20(1):e1011075. doi: 10.1371/journal.pgen.1011075 (PMC10786395; doi:10.1371/journal.pgen.1011075)
Supplement: S4 Table — Retention time (tR) in minutes [min], electrospray ionization (ESI) mode, mass-to-charge-ratio ((m/z)), declustering potential (DP) and collision energy (CE) both in volt [V], protonated adduct (M+H), deprotonated adduct (M-H), ammonium adduct (M+NH4) quantifier m/z is marked with *. (DOCX) [file pgen.1011075.s024.docx]

**S4 Table. Applied mass spectrometer and source parameters.** Retention time (t_R_) in minutes [min], electrospray ionization (ESI) mode, mass-to-charge-ratio (m/z), declustering potential (DP) and collision energy (CE) both in volt [V], protonated adduct (M+H), deprotonated adduct (M-H), ammonium adduct (M+NH_4_) quantifier m/z is marked with *.

| **Parameters for LC** | | **Parameters for MS/MS** | | | | | |
| --- | --- | --- | --- | --- | --- | --- | --- |
| **Compound** | **t_R_ [min]** | **ESI mode** | **Adduct** | **Precursor**  ***m/z*** | **Product**  ***m/z*** | **DP [V]** | **CE [V]** |
| GA3 | 6.23 | + | M+H | 347.4 | 111.0* 165.1 | 30.0 | 40.0  70.0 |
|  |  | - | M-H | 345.3 | 227.0* 221.1 | -243.0 | -30.0  -30.0 |
| GA1 | 6.30 | + | M+H | 349.0 | 303.3* 241.1 | 62.0 | 10.0  22.0 |
|  |  | - | M-H | 347.0 | 273.0* 229.1 | -130.0 | -30.0  -40.5 |
| FB1 | 7.44 | + | M+H | 722.4 | 352.2* 334.4 | 170.0 | 49.0  54.0 |
|  |  | - | M-H | 720.2 | 156.9* 562.3 | -90.0 | -47.0  -38.0 |
| FB2 | 8.28 | + | M+H | 706.4 | 336.3* 318.2 | 120.0 | 48.0  50.0 |
|  |  | - | M-H | 704.4 | 156.9* 546.3 | -290.0 | -42.0  -36.0 |
| GA7 | 9.22 | + | M+H | 331.5 | 239.1* 181.1 | 30.0 | 27.0  33.0 |
|  |  | - | M-H | 329.1 | 223.0* 211.0 | -207.0 | -23.0  -32.0 |
| GA4 | 9.32 | + | M+H | 333.5 | 269.0* 224.9 | 30.0 | 20.0  23.0 |
|  |  | - | M-H | 331.0 | 257.0* 213.0 | -250.0 | -29.0  -40.0 |
| Fusarin C | 10.15 | + | M+H | 432.0 | 115.0* 141.0 | 60.0 | 129.0 89.0 |
|  |  | - | M-H | 430.1 | 138.9* 357.3 | -120.0 | -22.0  -12.0 |
| BIK | 11.07 | + | M+H | 383.0 | 340.0* 355.0 | 263.0 | 45.0  38.0 |
| BEA | 14.62 | + | M+NH_4_ | 801.4 | 244.1* 134.0 | 120.0 | 41.0  90.0 |
|  |  |  | M+H | 784.4 | 244.0 262.0 | 259.0 | 33.0  31.0 |
